# Antagonistic and synergistic epigenetic modulation using orthologous CRISPR/dCas9-based modular system

**DOI:** 10.1093/nar/gkz709

**Published:** 2019-08-14

**Authors:** Goran Josipović, Vanja Tadić, Marija Klasić, Vladimir Zanki, Ivona Bečeheli, Felicia Chung, Akram Ghantous, Toma Keser, Josip Madunić, Maria Bošković, Gordan Lauc, Zdenko Herceg, Aleksandar Vojta, Vlatka Zoldoš

**Affiliations:** 1 Department of Biology, Division of Molecular Biology, Faculty of Science, University of Zagreb, Horvatovac 102a, 10000 Zagreb, Croatia; 2 Department of Chemistry, Division of Biochemistry, Faculty of Science, University of Zagreb, Horvatovac 102a, 10000 Zagreb, Croatia; 3 Epigenetics group, International Agency for Research on Cancer (IARC), 150 Cours Albert Thomas, Lyon, France; 4 Faculty of Pharmacy and Biochemistry, University of Zagreb, A. Kovačića 1, 10000 Zagreb, Croatia; 5 Genos Glycoscience Research Laboratory, Borogajska cesta 83 h, 10000 Zagreb, Croatia

## Abstract

Establishing causal relationship between epigenetic marks and gene transcription requires molecular tools, which can precisely modify specific genomic regions. Here, we present a modular and extensible CRISPR/dCas9-based toolbox for epigenetic editing and direct gene regulation. It features a system for expression of orthogonal dCas9 proteins fused to various effector domains and includes a multi-gRNA system for simultaneous targeting dCas9 orthologs to up to six loci. The C- and N-terminal dCas9 fusions with DNMT3A and TET1 catalytic domains were thoroughly characterized. We demonstrated simultaneous use of the DNMT3A-dSpCas9 and TET1-dSaCas9 fusions within the same cells and showed that imposed cytosine hyper- and hypo-methylation altered level of gene transcription if targeted CpG sites were functionally relevant. Dual epigenetic manipulation of the *HNF1A* and *MGAT3* genes, involved in protein *N*-glycosylation, resulted in change of the glycan phenotype in BG1 cells. Furthermore, simultaneous targeting of the TET1-dSaCas9 and VPR-dSpCas9 fusions to the *HNF1A* regulatory region revealed strong and persistent synergistic effect on gene transcription, up to 30 days following cell transfection, suggesting involvement of epigenetic mechanisms in maintenance of the reactivated state. Also, modulation of dCas9 expression effectively reduced off-target effects while maintaining the desired effects on target regions.

## INTRODUCTION

The exceptional versatility of RNA-guided CRISPR/Cas9 system has led to a shift from its primary use as molecular scissors for precise genome editing to a new role of a moiety for easily programmable targeting and delivery of various functional domains for various purposes: manipulation of selected DNA loci/sequences, multicolor imaging of chromosomal loci in living cells, tracking endogenous mRNAs and/or study of chromatin conformation and dynamics (1–5). Various domains for locus-specific transcriptional activation (e.g. VP64, VPR, p300) or repression (e.g. KRAB) can been fused to nuclease-dead Cas9 (dCas9) and such CRISPR/dCas9 systems, guided to the target sequences by single guide RNA molecules (gRNA), have been used in order to manipulate gene transcription (6–11). Recent studies demonstrated that CRISPR/dCas9-based molecular tools could be repurposed for simultaneous activation and repression of different genes. The Krüppel-associated box (KRAB) and VPR domains were fused independently to two different orthologous dCas9 proteins, isolated from *Streptococcus pyogenes* (SpCas9) and *Staphylococcus aureus* (SaCas9), and such molecular tools were then used for inducible orthogonal gene regulation within the same cell (12). This and similar CRISPR activation systems were successfully used for simultaneous activation of multiple loci *in vivo* and high-throughput screening of driver and suppressor genes involved in cancer cell growth (13,14). Such technology is extremely useful for manipulation of multiple candidate genes and has a huge potential for applications in modeling of developmental processes and disease.

A growing amount of publications demonstrates how environment and lifestyle factors are associated with coordinated changes in gene transcription due to epigenetic aberrations, and how epigenetic changes are key drivers of pathology in many cancers (15) but also in other complex diseases, including immune-mediated and inflammatory diseases (16–19). Therefore, identification of epigenetic aberrations and targeting them with new drugs, which are more precise compared to epigenetic inhibitors, have been the focus of the latest research in the fields of epigenetics, biomedicine and regenerative medicine (20). The methodology to engineer the epigenome using CRISPR/dCas9 could greatly contribute to recent advancements (21). So far, it has been demonstrated for fragile X syndrome, the most common genetic disorder, that restoration of aberrant *FMR1* gene expression can be achieved by reversing aberrant methylation at microsatellite repeats in proximity of the promoter of this gene using CRISPR/dCas9-TET1 molecular tool (22).

While simple fusions of DNMT3A and TET1 with dCas9 have already been described and used in several works (23–28), there is no universal and robust approach for creating active fusions of DNMT3A and TET1 with different dCas9 orthologs, nor a study demonstrating a simultaneous action of writers or erasers of epigenetic marks with opposite significance (activation versus silencing) within the same cells. Also, CRISPR/dCas9-based tools for simultaneous epigenetic editing have a potential to revolutionize the field of epigenetics by enabling researchers to determine causal relationship between directly manipulated individual epigenetic marks and gene transcription activity, as well as to help understand the complex chromatin layer involved in transcriptional regulation. To date, there is no clear picture of the causative relationship between a gene promoter methylation and transcriptional status of a certain gene, nor of the number and positions of CpGs in individual promoters that regulate transcription of the corresponding gene.

Based on our successfully constructed C-terminal dSpCas9-DNMT3A fusion (27), we aimed to design a backbone with exchangeable modules, thus creating a molecular toolbox that can be reused, extended with new functional modules and easily reconfigured for desired experimental setup (e.g. backbones for alternative delivery using lentiviruses etc.). Here, we describe the development and validation of a versatile platform for easy and convenient assembly of different promoters, dCas9 orthologs, effector domains (epigenetic modifiers or direct activators/silencers of gene transcription) and selection markers, along with specific gRNAs to target multiple candidate loci. We added the capability for assembling up to six gRNA-expression cassettes for both dSpCas9 and dSaCas9. Furthermore, we thoroughly characterized our new CRISPR/dCas9-based tools for epigenetic editing by examining activity profiles and time-course of their activity and demonstrating the capability of two different dCas9 fusions to perform antagonistic and synergistic activities within same cells at the same time. The candidate loci chosen for manipulation were those we study in the context of IgG glycosylation and its involvement in chronic inflammatory diseases (CID) (29). We demonstrated that dual epigenetic manipulation (i.e. DNA methylation and demethylation) of different pairs of individual loci within same cells resulted in changes in gene transcription level and consequently changes in the glycan phenotype, exemplifying the power of our molecular toolbox in testing the functional importance of modulating epigenetic states.

## MATERIALS AND METHODS

### Construction of the modular molecular toolbox by Golden Gate cloning

Details of the cloning steps, as well as the source plasmids, primers and oligonucleotides, plasmids for Cas9 fusions and the multi-guide system are given in the Supplementary Data. If applicable, plasmids are referenced by the number at the Addgene repository, where full sequence information is available. Assembly was done with 50 ng of the backbone plasmid pBackBone-BZ and each selected module vector, using molar ratio of 3:1 (module:backbone). Reaction was done in 1× CutSmart Buffer (New England Biolabs, Ipswich, MA, USA, NEB) with additional 1 mM ATP (NEB), 10 U of BsaI (NEB) and 350 U of T4 DNA Ligase (TaKaRa, Kusatsu, Shiga, Japan) with 30 cycles of 5 min at 37°C followed by 10 min at 16°C, after which ligase was inactivated at 50°C for 10 min. Next, BsaI was inactivated at 80°C for 10 min. Finally, 10 U of exonuclease V (RecBCD) (NEB) was added directly to the reaction along with additional 0.5 mM ATP and incubated at 37°C for 30 min to remove any remaining linear DNA. Following bacterial transformation and blue–white selection, white colonies were selected for further verification.

### Construction of multi-guide system

We used two strategies for assembly of up to six gRNA modules, each carrying a different gRNA molecule into the final construct for epigenetic modulation. One is to first assemble all functional modules with BsaI (described above) and to use an empty pSg-x1-6 plasmid (depending on the number of gRNA modules to be assembled). Next, individual gRNA molecules are cloned into plasmids pSgMxA or pSgMxG (pSgMxA represent plasmids for SaCas9 gRNA molecules while pSgMxG represent plasmids for SpCas9 gRNA molecules, x = 1, 2, 3, 4, 5, 6, depending on the order of gRNA modules to be assembled) as described (Supplementary Figure S3). Note that the pSgMxA or pSgMxG plasmids carry spectinomycin resistance, allowing counter-selection with both the backbone (ampicillin) and module (kanamycin) vectors. Finally, Esp3I assembly of plasmids for cloning of individual gRNA molecules into the finished BsaI assembled construct is done in 1 × Tango Buffer (Thermo Fisher Scientific, Waltham, MA, USA) with additional 1 mM ATP, 1 mM dithiothreitol (DTT) (Thermo Fisher Scientific), 10 U of restriction enzyme Esp3I (Thermo Fisher Scientific) and 350 U of T4 DNA Ligase with 10 cycles of 5 min at 37°C and 10 min at 16°C, followed with 15 min at 37°C and enzymes inactivation on 80°C for 10 min. The second strategy is to first assemble up to six gRNA ‘individual’ modules, each with inserted variable parts of gRNA molecule (as described, Supplementary Figure S4) into the empty pSg-x1-6 plasmid using the Esp3I restriction enzyme (described above). The resulting plasmid is a module vector containing multiple gRNA expression cassettes, which can be used as a standard gRNA module in the BsaI assembly.

### Cell culture and transfections

Human embryonic kidney cell line HEK293 (ATCC, CRL-1573) was maintained in Dulbecco’s Modified Eagle Medium (Sigma Aldrich, St. Louis, MO, USA) supplemented with 10% fetal bovine serum (Sigma Aldrich), 4 mM L-glutamine (Sigma Aldrich), 100 U/ml penicillin and 100 μg/ml streptomycin (Sigma Aldrich). BG1, the ovarian cancer cell line, were cultivated in RPMI 1640 (Sigma-Aldrich, Missouri, USA) supplemented with 10% heat-inactivated fetal bovine serum (Sigma-Aldrich), 4 mM L-glutamine (Sigma-Aldrich), 100 U/ml penicillin and 100 μg/ml streptomycin (Sigma-Aldrich). All cells were incubated at 37°C in a humidified 5% CO_2_-containing atmosphere.

Transfections of HEK293 cells were done using Lipofectamine 3000 Reagent (Invitrogen, Carlsbad, CA, USA) according to the manufacturer’s protocol, while PEI MAX 40K (Polysciences, Warrington, PA, USA) was used for transfection of BG1 cells. Briefly, HEK293 cells were seeded in 24- or 6-well plates day before transfection and transfected next day at around 80% of confluency with up to 1 μg of plasmid DNA. BG1 cells were seeded in 100-mm culture dishes and were transfected next day with 5 μg of individual plasmid DNA or total 10 μg of plasmid mixtures in antibiotic- and serum-free RPMI medium. The used ratio of PEI to DNA was 3:1. Cells were screened 24h post transfection for expression of fluorescent proteins (mClover3 and/or mRuby3), translated in the same reading frame as the dCas9 fusions and were selected with puromycin (Gibco Life Technologies, Grand Island, NY, USA) for 48 h. Cells were then collected at each time point depending on experiment (described below) for subsequent DNA, RNA and/or protein isolation.

### Total DNA, RNA and protein isolation

In all experiments, DNeasy Blood & Tissue Kit (Qiagen, Hilden, Germany) was used for total DNA isolation, while RNA was isolated using RNeasy Mini Kit (Qiagen). In experiments of simultaneous cytosine methylation and demethylation of two different gene loci in BG1 cells, total proteins were precipitated using methanol and chloroform standard protocol after cell disruption in lysis buffer (50 mM Tris pH 7.4, 0.1% Triton X, 1 mM EDTA, 135 mM sodium chloride), supplemented with Protease Inhibitor Cocktail (cOmplete™ ULTRA Tablets, EDTA-free, glass vials Protease Inhibitor Cocktail, Roche, Basel, Switzerland).

### Bisulfite conversion and pyrosequencing

Bisulfite conversion of 500 ng of DNA was done with EZ DNA Methylation-Gold Kit (Zymo Research, Irvine, CA, USA) according to the manufacturer’s protocol, after which the genomic regions of the *BACH2, MGAT3, IL6ST, LAMB1* and *HNF1A* genes were amplified using a PyroMark PCR kit (Qiagen). Amplified genomic regions were then sequenced using the PyroMark Q24 Advanced pyrosequencing system (Qiagen) and PyroMark Q24 Advanced CpG Reagents (Qiagen) to quantify methylation level at individual CpG dinucleotides. Assay sequences are listed in Supplementary Table S1, and assay maps are shown in Figures 4, 5 and 6. Sequences of PCR and pyrosequencing primers are listed in Supplementary Table S2.

### Quantitative real-time PCR

Reverse transcription was done on 500 ng of total isolated RNA (prior treated with TURBO DNase (Invitrogen)) using the PrimeScript RTase (TaKaRa) and random hexamer primers (Invitrogen). RT-qPCR was performed according to the manufacturer’s protocol using the 7500 Fast Real-Time PCR System, using TaqMan Gene Expression Master Mix and the following TaqMan Gene Expression Assays (Applied Biosystems, Foster City, CA, USA): Hs00167041_m1 (*HNF1A*), Hs02800695_m1 (*HPRT1*), Hs02379589_s1 (*MGAT3*), Hs00174360_m1 (*IL6ST1*) and Hs00222364_m1 (*BACH2*), according to manufacturer’s protocol. For detection of the longer *MGAT3* transcript (targeting of TIS region), the Hs013692440_m1 TaqMan probe was used. Gene expression was normalized to the *HPRT1* gene and analyzed using the comparative *C*_t_ method (30). Expression was shown in all experiments as fold change (FC) relative to mock-transfected cells.

### Time-course evaluation of C-terminal and N-terminal fusions of TET1 and DNMT3A catalytic domains to dSpCas9 and/or dSaCas9

Time-course evaluation of C-terminal fusion dSpCas9-TET1 was done using dSpCas9-TET1-PuroR plasmids encoding MGAT3-sg03 or LAMB1-sg01 (Supplementary Table S3). Transfections were done in biological triplicates, where control groups were cells transfected with either dSpCas9-TET1-PuroR_NCTRL (inactive fusion) co-expressing targeting gRNA or mock-transfected cells. Transfected cells were collected daily until the 8th day and later on 10th, 15th, 20th, 24th and 30th day for DNA isolation. In addition, time-course evaluation of N-terminal fusions DNMT3A-dSpCas9 and TET1-dSaCas9 was performed. DNMT3A-dSpCas9 construct was targeted to *IL6ST* using multi-guide system with four gRNA molecules, while TET1-dSaCas9 construct was targeted to *HNF1A* locus using multi-guide system with two gRNA molecules (Supplementary Table S3). For both experiments, cells were collected on 5th, 8th, 11th, 20th and 30th day after the transfection, while non-targeting gRNA plasmids (NT-gRNA; gRNA with no target sequence in the human genome) and mock-transfected cells were used as controls.

### Analyses of activity profiles for C- and N-terminal fusions of DNMT3A and TET1 catalytic domains to dCas9 from *Streptococcus pyogenes* and *Staphylococcus aureus*

All experiments were done with plasmids co-expressing certain dCas9 fusion and chimeric gRNA in biological duplicates, where control groups were catalytically active constructs co-expressing non-targeting gRNA and mock-transfected cells. For obtaining the activity profile of dSpCas9-TET1 C-terminal fusion, the *MGAT3* promoter was targeted using eight gRNAs: MGAT3-sg01 to MGAT3-sg08 and *LAMB1* promoter was targeted using two gRNAs (Supplementary Table S3), while the activity profile of dSpCas9-DNMT3A C-terminal fusion was described previously (27). In experiments with N-terminal fusions of dCas9 (from either *Streptococcus pyogenes*, dSpCas9 or *Staphylococcus aureus*, dSaCas9) with DNMT3A or TET1 catalytic domains, specific gRNAs targeting the *BACH2* and *MGAT3* promoters were used. The DNMT3A-dSpCas9 was targeted with seven gRNAs and DNMT3A-dSaCas9 with six gRNAs to unmethylated region of the *BACH2* promoter. The TET1-dSpCas9 and TET1-dSaCas9 fusions were targeted with five gRNAs to highly methylated region of the *MGAT3* promoter (Supplementary Table S3).

### Simultaneous manipulations of two different gene loci using dCas9-based tools with antagonistic functionalities in HEK293 and BG1 cells

In experiments of simultaneous methylation and demethylation in HEK293 cells, we targeted gene pairs *BACH2–HNF1A* and *IL6ST–MGAT3*. The *MGAT3* and *HNF1A* genes were targeted with TET1-dSaCas9 N-terminal fusions containing mClover3 as fluorescence marker, while *IL6ST* and *BACH2* were targeted with DNMT3A-dSpCas9 N-terminal fusions with mRuby3. All gene loci were targeted using the multi-guide module: the *MGAT3* was targeted with six gRNAs, *HNF1A* with two gRNAs, while *IL6ST* and *BACH2* were targeted each with four gRNAs (Figures 4 and 5; Supplementary Table S3). Cells were collected on 8th day following transfection for subsequent DNA and RNA isolation. We also performed time-course experiment for monitoring CpG methylation and gene expression with the same setup as described above and collected total DNA and RNA on 5th, 8th, 9th and 12th day after cell transfection (Supplementary Figure S6). In BG1 cells, we targeted *HNF1A*–*MGAT3* gene pair for simultaneous methylation and demethylation. The TET1-dSaCas9 along with mClover3 was targeted to highly methylated region of the *HNF1A* promoter using the multi-guide module containing two gRNAs, while the DNMT3A-dSpCas9 fusion along with mRuby3 was targeted to unmethylated TIS region of the *MGAT3* promoter with one gRNA (Figure 6A and Supplementary Table S3). BG1 cells were collected on 7th day following transfection for subsequent DNA, RNA and protein isolation. All dCas9 fusions had puromycin resistance, which was added using the dual-marker system (Figure 3C). Three types of negative controls were used in experiments: (i) plasmids with inactive DNMT3A and TET1 catalytic domains; (ii) plasmids with non-targeting gRNAs (NT-gRNA); and (iii) mock-transfected cells. Transfections were done in nine and three biological replicates for experiments performed on HEK293 and BG1 cells, respectively.

### Analysis of total *N*-glycome in BG1 cells

The dried protein pellets were resuspended in 30 μl of 1.33% (wt./vol.) SDS (Invitrogen) and incubated at 65°C for 10 min. Subsequently, 10 μl of 4% Igepal-CA630 (Sigma Aldrich) and 1.2 U of PNGase F (Promega, Madison, WI, USA) in 10 μl 5 × PBS were added to each sample and incubated overnight at 37°C to allow release of N-glycans. The released N-glycans were labeled with procainamide (Sigma Aldrich), followed by hydrophilic interaction liquid chromatography solid-phase extraction (HILIC-SPE) clean-up. Fluorescently labeled and purified N-glycans were separated by HILIC on Waters Acuity ultra-performance liquid chromatography (UPLC) instrument (Waters, Milford, MA, USA) consisting of a quaternary solvent manager, sample manager and a fluorescence detector set with excitation and emission wavelengths of 310 and 370 nm, respectively. The instrument was under the control of Empower 2 software, build 2145 (Waters). Plasma N-glycans were separated on a Waters bridged ethylene hybrid (BEH) Glycan column, 150 × 2.1 mm, 1.7 μm BEH particles, with 100 mmol/l ammonium formate, pH 4.4, as solvent A and acetonitrile as solvent B. The separation method used a linear gradient of 70–53% acetonitrile (vol./vol.) at flow rate of 0.561 ml/min in a 25 min analytical run. Data were processed using an automated integration method and the chromatograms were all separated in the same manner into 31 peaks (GP1–GP31), where the content of glycans in each peak was expressed as a percentage of the total integrated area. All glycan structures were annotated with MS/MS analysis by HILIC-UPLC coupled with a Synapt G2-Si ESI-QTOF-MS system (Waters). The instrument was under the control of MassLynx v.4.1 software (Waters). MS conditions were set as follows: positive ion mode, capillary voltage 3 kV, sampling cone voltage 30 V, source temperature 120°C, desolvation temperature 350°C, desolvation gas flow 800 l/h. Mass spectra were recorded from 500 to 3000 *m*/*z* at a frequency of 1 Hz. MS/MS experiments were performed in a data-dependent acquisition (DAD) mode. Spectra were first acquired from 500 to 3000 *m*/*z* and then two precursors with the highest intensities were selected for CID fragmentation (*m*/*z* 100 to 3000 was recorded). A collision energy ramp was used for the fragmentation (LM CE Ramp Start 7 V, LM CE Ramp End 13 V, HM CE Ramp Start 97 V, HM CE Ramp End 108 V).

### Time-course experiment using VPR-dSpCas9 and TET1-dSaCas9 in HEK293 cells

Highly methylated regulatory region of *HNF1A* was simultaneously targeted with TET1-dSaCas9 and the VPR-dSpCas9 N-terminal fusions in HEK293 cells. Two gRNAs using multi-guide system were used to target TET1-dSaCas9 (as in Figure 4B), while VPR-dSpCas9 was targeted with one gRNA (Supplementary Figure S8 and Supplementary Table S3). The VPR-dSpCas9 construct had mRuby3, while TET1-dSaCas9 had mClover3 as fluorescent marker and both constructs had puromycin resistance, which was added using the dual-marker system. One transfection was done with the mixture of VPR-dSpCas9 and TET1-dSaCas9 fusions, while the other two transfections were done with individual VPR-dSpCas9 and TET1-dSaCas9 fusion to assess the contribution of each domain separately. Cells were collected on 5th, 8th, 11th, 20th and 30th day after the transfection for subsequent DNA, RNA and protein isolation. Transfections were done in seven biological replicates with NT-gRNA and mock as negative controls.

### Time-course evaluation of the TET1-dSaCas9 presence in HEK293 cells using quantitative real-time PCR

To determine presence of TET1-dSaCas9 fusion in cells at level of DNA (presence of plasmid DNA), RNA (presence of mRNA transcript) and protein (dCas9), we performed qPCR analysis and western blotting. HEK293 cells were transfected with the TET1-dSaCas9 fusion and targeted to the *HNF1A* regulatory region using the multi-guide module containing two gRNAs. Cells were collected on 5th, 8th, 11th, 20th and 30th day after transfection. The amount of the TET1-dSaCas9 plasmid was determined relative to the genomic *RAG1* sequence (31), while the amount of mRNA transcript was normalized to the *GAPDH* gene expression using the comparative *C*_t_ method (30). Reactions were done using Power SYBR Green PCR Master Mix (Applied Biosystems), 50 nM of primers and 4 ng of total isolated DNA or 10 ng of cDNA. Reaction conditions were as following: 10 min at 95°C followed by 40 cycles at 95°C for 15 s and 60°C for 1 min (for detection of TET1-dSaCas9 plasmid) or 54°C for 1 min (for detection of TET1-dSaCas9 cDNA), followed by melt curve analysis of amplified targets. All primers used for real-time qPCR are listed in Supplementary Table S5.

### Detection of dCas9 protein by western blotting

We also checked the presence of TET1-dSaCas9 protein at each time point: 5^th^, 8^th^, 11^th^, 20^th^ and 30^th^ day after HEK293 cell transfection. To prove that the weak EFS promoter, in fusions containing a ‘secondary cassette’ (for separate expression of antibiotic resistance marker under the stronger SV40 promoter, see Supplementary Figure S5), directly limits the amount of fusion proteins (DNMT3A-dSpCas9 and TET1-dSpCas9) compared to dCas9 fusions where the strong CBh promoter drives expression of both the fusion protein and the puromycin resistance, we compared the amount of the fusion proteins at 4th and 8th day after cell transfection. In both experiments, cells were lysed in RIPA buffer supplemented with Protease Inhibitor Cocktail (cOmplete™ ULTRA Tablets, Roche) and sonicated in Sonorex Super Ultrasonic bath (Bandelin, Berlin, Germany). Total protein concentration was determined with BCA Protein Assay Kit (Santa Cruz Biotechnology, Dallas, TX, USA) according to the manufacturer’s protocol and 40 μg of protein extract was used for standard immunoblotting with β-tubulin as a loading control. Rabbit anti-SaCas9 1:5000 (ab203943, Abcam, Cambridge, UK), mouse anti-SpCas9 1:1000 (ab191468, Abcam), rabbit anti- β-Tubulin 1:2000 (ab6046, Abcam), goat anti-Rabbit IgG H&L (HRP) 1:200 000 (ab6721, Abcam) and goat Anti-Mouse IgG H&L (HRP) 1:200 000 (ab205719, Abcam) antibodies were used. The antibody-tagged protein bands were visualized by Immobilon Western Chemiluminescent HRP Substrate (Milipore, Burlington, MA, USA) according to the manufacturer’s protocol. ECL signals captured on Alliance Q9 Advanced Chemiluminescence Imager (Clever Scientific, Warwickshire, UK) were quantified using the ImageJ software (32).

### Whole-genome methylation analysis by Infinium MethylationEPIC (850K) array

To compare the effect of two types of dCas9 fusions (one with weak EFS promoter and another with strong CBh promoter, driving the expression of both dCas9 fusion protein and puromycin resistance gene) on global off-target activity, the whole-genome methylation analysis was performed. The DNMT3A-dSpCas9 was targeted to the *IL6ST* promoter using the multi-guide module containing four gRNAs and the TET1-dSpCas9 was targeted to *MGAT3* using the multi-guide module consisting of five gRNAs (Figure 8 and Supplementary Table S3). For each locus, two types of dCas9 fusion constructs were used: (i) the ‘primary cassette’ construct, whereby both the fusion protein and the selection marker (PuroR) were under the strong constitutive CBh promoter; (ii) the ‘secondary cassette’, whereby the dCas9 fusion construct was expressed under the weak EFS promoter, while maintaining efficient selection of transfected cells with PuroR under the strong SV40 promoter. Transfection of HEK293 cells was done in biological and technical duplicates and cells were harvested 8th day after transfection for DNA isolation. DNA (600 ng) was subjected to bisulfite conversion and then methylome profiling was done using the Infinium MethylationEPIC (850K) array. Of the array containing total of 866 091 probes, 793 038 probes remained for further analysis after removing cross-reactive probes, probes overlapping genetic variants at targeted CpG sites, probes with genetic variants overlapping the body of the probe and XY chromosome probes, as well as probes reporting missing values in more than 5% of all samples.

### Statistical analysis and data representation

Changes in CpG methylation and expression levels, as well as changes in glycan structure ratios, were analyzed using the Mann–Whitney test. All data were visualized using the R Language and Environment for Statistical Computing (R Foundation for Statistical Computing, Vienna, Austria). Images from western blotting experiments were processed and quantitated using the ImageJ program (32). Pre-processing and normalization of whole methylome data were done using the ‘minfi’ Bioconductor package (33). PCA analysis followed by Wilcoxon Rank Sum Tests was conducted to identify possible confounding variables. SVA was performed to correct for potential batch effects. We converted β-values converted to *M*-values, and a linear regression model with adjustments for Sentrix ID was used to identify differentially methylated probes (DMPs) using the Bioconductor ‘limma’ package (34). Statistically significant DMPs were defined as those with false discovery rate (FDR)-adjusted *P*-values of < 0.05, and delta-beta values of > 0.05 or < −0.05.

The ‘IlluminaHumanMethylationEPICanno.ilm10b2.hg19’ annotation package was used to map DMPs to CpG annotations, i.e. CpG islands, CpG shores, CpG shelves or open sea regions. The ‘annotatr’ Bioconductor package was used to map the DMP regions to chromatin states based on annotations defined by chromHMM in Hmec cells. Fold enrichment of each category based on relationship to CpG islands and regulatory regions were calculated by dividing the fraction of DMPs in each annotation category with the fraction of all filtered probes mapped to the respective category, correcting for differences in representation of each annotation category on the Infinium MethylationEPIC array.

## RESULTS

### Active fusions of DNMT3A and TET1 catalytic domains with the dCas9 orthologs

**Figure 1.**
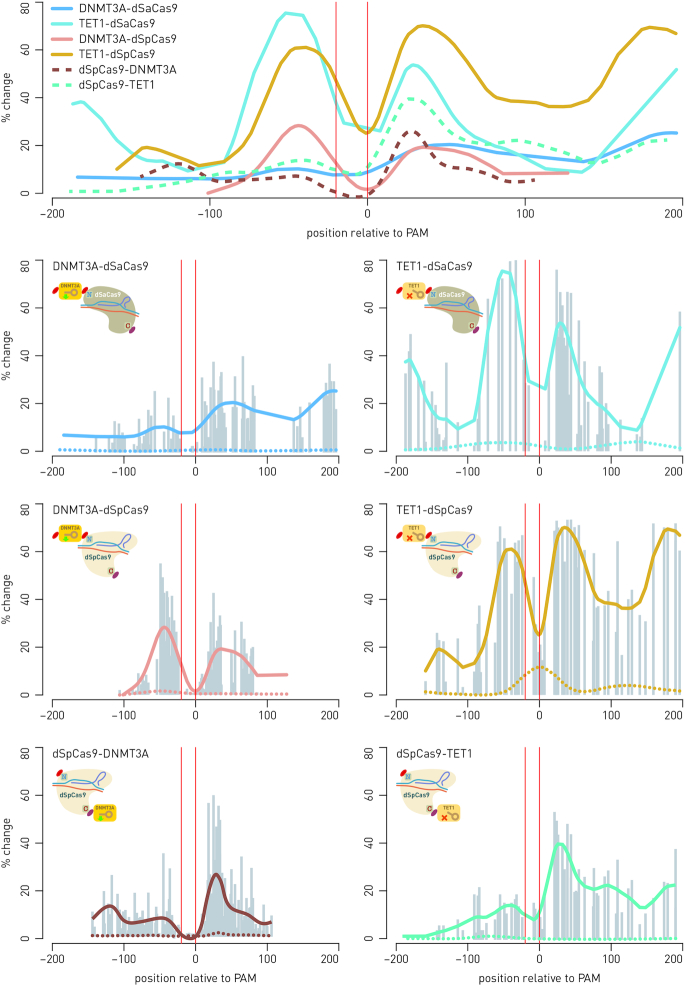
N- and C-terminal fusions of DNMT3A and TET1 catalytic domains to dCas9 orthologs from *Streptococcus pyogenes* and *Staphylococcus aureus* show similar activity profiles. Top panel shows summary of similar activity profiles for all combinations of effector domains (catalytic domains of DNMT3A or TET1) to C- or N-terminus of either dSpCas9 or dSaCas9 orthologs. The dSpCas9 C-terminal fusions show activity concentrated mainly downstream from the targeted sequence, while all N-terminal fusions had activity distributed equally up- and downstream from the gRNA binding site. The peak of change in CpG methylation appears at around 30 bp on either side of the dCas9-binding site (indicated by vertical red lines). The percentage of change is defined as absolute increase (for fusions with DNMT3A) or decrease (for fusions with TET1) in CpG methylation level (averaged from multiple independent experiments). Individual figures represent profiles of individual dCas9-fusions and summarize multiple experiments as a result of targeting certain loci using specific gRNA molecules: fusions DNMT3A-dSpCas9 and DNMT3A-dSaCas9 were targeted to *BACH2* using six gRNAs specific for dSpCas9, or five gRNAs specific for dSaCas9, and one dual gRNA for both orthologs; fusions TET1-dSpCas9 and TET1-dSaCas9 were targeted to *MGAT3* using four gRNAs specific for dSpCas9 and four gRNAs specific for dSaCas9, and one dual gRNA for both orthologs; fusion dSpCas9-TET1 was targeted to *MGAT3* using eight gRNAs, and fusion dSpCas9-DNMT3A was targeted to *BACH2* and *IL6ST* using eight gRNAs and four gRNAs, respectively (27). Gray bars show data points from individual experiments and represent methylation change at a certain distance from the gRNA binding site. Dotted lines in individual figures of the activity profiles show that catalytically inactive fusions elicit no changes of methylation level.

**Figure 2.**
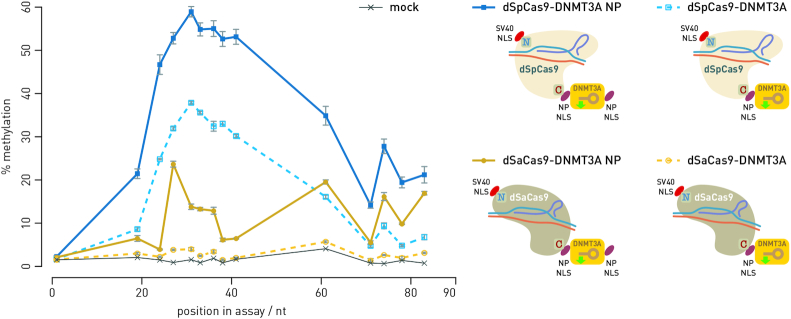
Nuclear localization signal (NLS) is critical for activity of an effector domain fused to C-terminus of dCas9. An additional nucleoplasmin NP-NLS appended to the C-terminus of the catalytic domain of DNMT3A clearly showed an increase in activity of the dCas9 fusion (solid lines) compared to activity obtained using the dCas9 fusions without additional NP-NLS (dotted lines). Each data point represents one of the 14 CpG sites analyzed for methylation level in the BACH2-A2 assay.

Based on our successfully constructed C-terminal dSpCas9-DNMT3A fusion (27), we aimed to create a modular toolbox with exchangeable dCas9 orthologs and fused various effector domains (DNMT3A, TET1, VPR and KRAB). To this end, we needed to test each configuration for activity in cell lines, i.e. verify that the fusions show the intended activity. First, we fused the TET1 catalytic domain to the C-terminus of dSpCas9 (analogous fusion to dSpCas9-DNMT3A; (27)) and targeted the *MGAT3* promoter with eight gRNAs and the *LAMB1* promoter with two gRNAs. The dSpCas9-TET1 fusion showed very similar activity profile to the analogous dSpCas9-DNMT3A fusion (Figure 1 and (27)). Then, we tested C-terminal fusions of the DNMT3A and TET1 catalytic domains to the dCas9 from *S. aureus*. Both C-terminal fusions revealed a poor activity in HEK293 transfected cells (data not shown). We speculated that it had been caused by steric hindrances or incorrect orientation of the catalytic domain, so we went on to test the effect of the tryptophan zipper motif (35) or the Inntag (36) as linkers, none of which restored catalytic activity (data not shown). Next, we tested whether a C-terminal nuclear localization signal (NLS) on dSaCas9 in the fusion protein was rendered inaccessible after appending an effector domain (DNMT3A) to the C-terminus, thus interfering with import into the nucleus and consequently lowering the measured activity of the effector domain. Indeed, the dSaCas9-DNMT3A fusion acquired activity when an additional nucleoplasmin NLS was appended to the C-terminus of the catalytic domain (Figure 2). Although the dSpCas9-DNMT3A fusion has already shown appreciable methylation activity (27), its activity was further enhanced after addition of another nucleoplasmin NLS on the C-terminus of the catalytic domain (Figure 2).

The fusion of DNMT3A with dCas9 from *S. aureus*, with an additional nucleoplasmin NLS added to the catalytic domain, still achieved only 2% to maximally 24% of methylation level increase, depending on CpG site (Figure 2). In comparison with fusion of DNMT3A with dCas9 from *S. pyogenes*, which achieved up to 60% increase of methylation level (27), the activity of the dSaCas9-DNMT3A fusion seemed unsatisfactory. Therefore, the next step was to fuse the catalytic domain to N-terminus of dCas9 in search for a universal approach compatible with all effector domains. Indeed, more robust activity of both DNMT3A and TET1 catalytic domains in N-terminal fusions with dCas9 from *S. aureus* (as well as with *S. pyogenes*) was achieved. Therefore, this configuration was selected for the modular approach. Comparing the two orthologs, the dSpCas9 N-terminal fusions consistently showed stronger activity than dSaCas9 N-terminal fusions (Figure 1). For clarity, in further text we designated C-terminal fusions as dSpCas9-DNMT3A and dSaCas9-TET1, and N-terminal fusions as DNMT3A-dSpCas9 and TET1-dSaCas9.

### Activity profiles and time course of activity for the C- and N-terminal fusions of DNMT3A and TET1 catalytic domains to dCas9 from *S. pyogenes* and *S. aureus*

The individual profiles showing activity of N-terminal fusions of DNMT3A and TET1 catalytic domains with dCas9 from *S. pyogenes* and *S. aureus*, as well as C-terminal fusion of TET1 and DNMT3A catalytic domains to dCas9 from *S. pyogenes* are presented in Figure 1 along with the summary activity profile. The profiles show average activity of DNMT3A or TET1 (seen as the level of CpG methylation or demethylation, and named % of change) with respect to the gRNA-binding site. The activity profiles were created by collecting data from many experiments, where each data point represents absolute increase or decrease of CpG methylation percentage, positioned relative to the gRNA-binding site; finally, a smoothing curve was drawn to represent the summary activity profile. The activity profiles of all N- and C-terminal fusions were similar (Figure 1), except for C-terminal fusion of DNMT3A and TET1 to dCas9 from *S. aureus*, which showed very poor activity (data not shown). In addition, C-terminal fusions with dCas9 from *S. pyogenes* had their activity concentrated mainly downstream from the targeted sequence, while the N-terminal fusions had activity distributed equally up- and downstream from the gRNA-binding site. The activity profiles of all dCas9 fusions, along a linear DNA molecule, revealed a peak of methylation change about 30 bp downstream from the gRNA-binding site, with some additional activity peaks detected around 180–200 bp upstream or downstream from the gRNA-binding site, indicating possible involvement of adjacent nucleosomes. Similarity among all six profiles demonstrates that effector domains fused to either end of dCas9 contact DNA in a similar manner.

A time-course experiment for dSpCas9-TET1 revealed maximum of the demethylation change within 7–8 days after transfection, with the effect slowly declining afterwards, but remaining substantial at the main activity site (30 bp downstream from gRNA-binding site) even after 30 days (up to 19% for *MGAT3* and up to 30% for *LAMB1*); meanwhile, the distal site at 120 bp downstream from gRNA reverted to its initial methylation level after 20–24 days (Supplementary Figure S1). A time course for the N-terminal fusions of DNMT3A-dSpCas9 and TET1-dSaCas9 targeting the *IL6ST* and *HNF1A* genes, respectively, is given in Supplementary Figure S2. The maximal effect of DNMT3A-dSpCas9 and TET1-dSaCas9 fusions was detected 8th and 11th day following transfection, while the effect of methylation/demethylation remained altered from 30% to 40% even 30 days following transfection of HEK293 cells.

### Modular CRISPR/dCas9-based toolbox for epigenetic modulation and gene regulation

**Figure 3.**
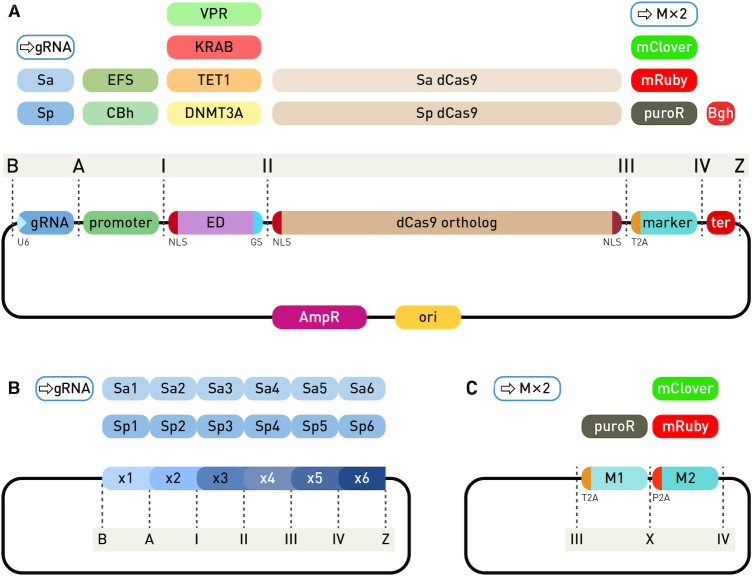
Modular system for CRISPR/dCas9-based epigenetic editing and direct gene regulation. (**A**) Individual modules of an expression cassette for N-terminal dCas9 fusions are assembled into the backbone vector using Golden Gate cloning with the BsaI type IIS restriction enzyme. The backbone plasmid has ends compatible with ‘B’ and ‘Z’ type module ends. The first position (‘B’ to ‘A’) receives a gRNA expression module with SaCas9 or SpCas9 scaffold, either containing a pre-inserted variable gRNA region, an empty module for gRNA cloning with red-white selection or a multi-guide module for a second step insertion of up to six gRNAs. Next position (‘A’ to ‘I’) is for insertion of a eukaryotic promoter, followed by the effector domain (‘I’ to ‘II’) containing an N-terminal NLS and a short G_4_S linker to a dCas9 ortholog (‘II’ to ‘III’), followed by a selection marker (fluorescence or antibiotic resistance, ‘III’ to ‘IV’) linked via the self-cleaving T2A peptide, which can be substituted with the dual-marker system. Finally, a module for eukaryotic transcription terminator is inserted between ends ‘IV’ and ‘Z’. (**B**) The multi-guide system accepts up to six gRNA modules for either dSpCas9 or dSaCas9 protein. Individual modules require the variable part of gRNA pre-cloned for the second step of assembly, facilitated by the type IIS restriction enzyme Esp3I and red–white selection. (**C**) The dual marker system enables addition of both antibiotic resistance and a fluorescent protein. The dual modules have T2A and P2A self-cleaving peptides for equimolar expression with dCas9.

Based on the activity profiles of C- and N-terminal fusions of effector domains to orthologous dCas9 proteins, we have developed a modular CRISPR/dCas9-based system for rapid assembly of desired constructs based on Golden Gate cloning (Figure 3A). A minimal bacterial backbone carrying pUC19 origin of replication and ampicillin resistance received six or seven modules with functional units released using the BsaI enzyme in a single Golden Gate reaction (Supplementary Figure S3). Non-palindromic four nucleotide (nt) overhangs linking functional units ensured accurate assembly, while blue–white selection enabled rapid identification of correctly assembled clones. The overnight reaction had nearly 100% efficiency, while a shortened reaction time (2 h) assembly was sufficient when used in conjunction with blue–white selection. The final constructs comprised a gRNA unit (either a single gRNA under the U6 promoter or a multi-guide system module), the eukaryotic promoter (CBh or EFS), an effector domain fused N-terminally to dCas9 ortholog of choice, a marker (or the dual marker system) linked using a self-cleaving 2A peptide and a eukaryotic transcriptional terminator.

For targeting, a 20 nt long gRNA fragment complementary to the genomic target was cloned into an appropriate gRNA module containing a U6 promoter and gRNA scaffold for either *S. pyogenes* or *S. aureus* using the BpiI Golden Gate enzyme and red–white selection for rapid identification of positive clones (Supplementary Figure S4). A gRNA could be cloned into the module either before or after assembly. For inclusion of up to six gRNA expression cassettes (comprising U6 promoter, cloning site, gRNA scaffold and U6 terminator) in a single construct, the multi-guide system was developed (Figure 3B). A special multi-gRNA module allowing for red–white screening and insertion of a fixed number of gRNA cassettes (1–6) can be included into the assembly. Target specificity was given to individual gRNA modules for positions 1–6 by inserting the 20 nt complementary sequence using the same BpiI strategy as for single gRNAs. Individual gRNA-preloaded modules were linked together in a secondary Golden Gate assembly with the Esp3I enzyme. The Esp3I assembly step was successfully used both for creation of a multi-guide module and for direct cloning into the fusion construct following a BsaI assembly of functional units. Both *S. pyogenes* and *S. aureus* scaffolds have independent modules for all six positions; they can be mixed and matched in a single multi-guide assembly. Each gRNA is specific for its cognate ortholog and can guide dCas9 when expressed *in trans*. For concurrent antibiotic selection and fluorescent protein (FP) tracking, we developed a dual marker system (Figure 3C). In addition to individual marker modules for antibiotic resistance (PuroR) and fluorescent proteins mClover3 (a GFP variant) and mRuby3 (an RFP variant), we added another optional non-palindromic 4 nt overhang for joining a T2A-linked antibiotic resistance gene to a P2A-linked fluorescent protein. The two 2A self-cleaving peptides enabled co-expression of both markers with the selected dCas9 fusion (Figure 3C). Although the system was initially designed for construction of a special dual marker module, we found the Golden Gate cloning approach to be sufficiently robust for direct assembly of all seven modules (instead of six ‘standard’ modules) in a single BsaI reaction.

### Simultaneous epigenetic manipulation of two genes using dCas9 fusions with antagonistic activities in HEK293 cells

**Figure 4.**
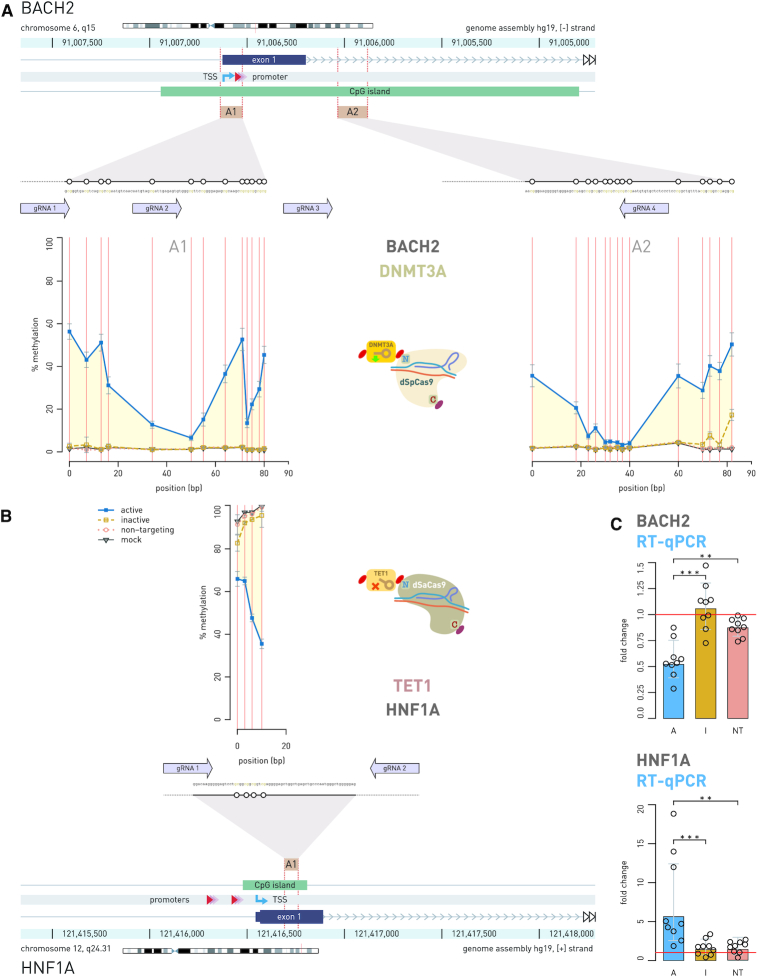
Simultaneous epigenetic manipulation of the *BACH2* and *HNF1A* loci using DNMT3A-dSpCas9 and TET1-dSaCas9 in HEK293 cell line. (**A**) Twenty seven CpG sites in CpG island of the *BACH2* promoter which was targeted with DNMT3A-dSpCas9 guided by co-expression of four different gRNA molecules using the multi-guide system, led to increase of methylation level ranging from 2% to 55% depending on the CpG site. (**B**) At the same time in the same cells, two gRNAs targeted TET1-dSaCas9 to four CpG sites located in the first exon of *HNF1A*, which led to decrease in methylation level of 27%, 32%, 49% and 65% for CpG1, CpG2, CpG3 and CpG4, respectively. (**C**) Induced changes in CpG methylation level were followed by changes in gene transcript level of both genes: *HNF1A* showed 5.614-fold change (*P* = 0.0005 to inactive TET1) and *BACH2* showed 0.568-fold change (*P* = 0.0003 to inactive DNMT3A). The human *BACH2* locus (**A**) and *HNF1A* locus (**B**) are shown with positions of the pyrosequencing assays (A1, A2) indicated by yellow rectangles. Magnified insets show individual CpG sites (white circles) targeted by dCas9 fusions and subsequently analyzed for methylation level. Arrows aligned to the magnified regions indicate 20 bp binding sites of gRNAs used to guide the dCas9 fusions. Arrows point toward the PAM sequence. Annotated promoter is represented by shaded red triangles; TSS, transcription start site (kinked arrow); A, dCas9 fusion with active catalytic domain; I, dCas9 fusions with inactive catalytic domain; NT, active dCas9 fusion with non-targeting gRNA. ** *P* < 0.01, *** *P* < 0.001.

**Figure 5.**
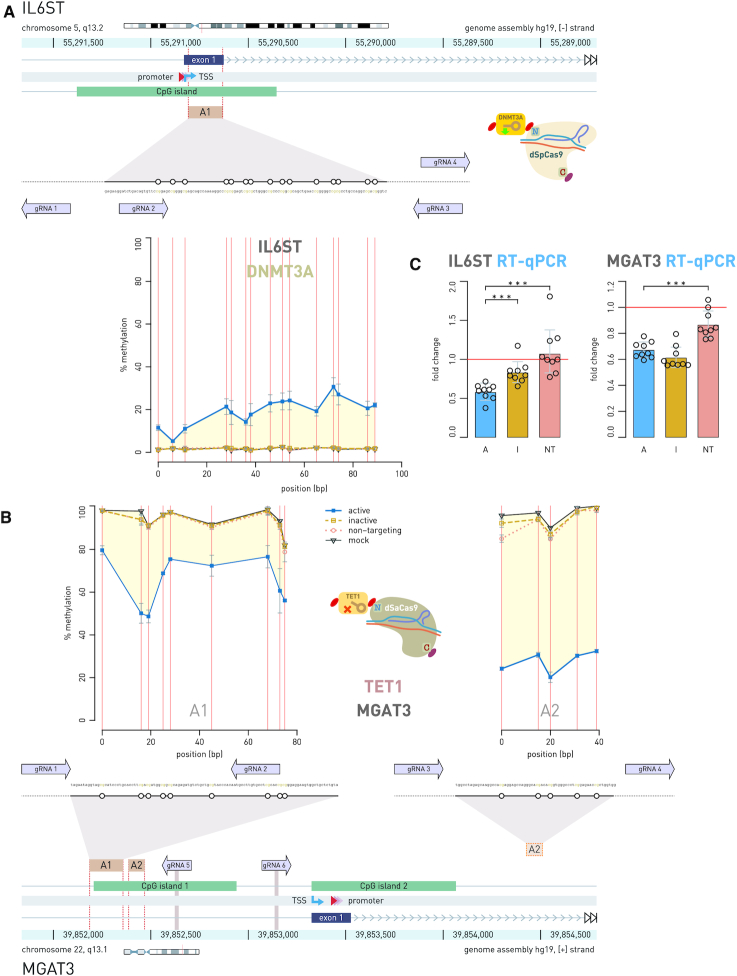
Simultaneous epigenetic manipulation of the *IL6ST* and *MGAT3* loci using DNMT3A-dSpCas9 and TET1-dSaCas9 in HEK293 cell line. (**A**) Fifteen CpG sites within CpG island of the *IL6ST* promoter were targeted with DNMT3A-dSpCas9, guided by the multi-guide system including four gRNAs, while 14 CpG sites located in CpG island 1 of the *MGAT3* promoter were targeted with TET1-dSaCas9, guided by the multi-guide system consisting of six gRNAs. (**B**) Doubly transfected cells with the two dCas9 fusions showed concurrent increase and decrease of methylation level at the targeted CpG sites: 3–29% methylation change for *IL6ST*, and 19–72% methylation change for *MGAT3*, depending on the CpG site. (**C**) While change of methylation level in *IL6ST* promoter was followed with 0.583-fold change in transcript level (*P* = 0.0002 to inactive DNMT3A), the hypomethylation of 14 CpG sites in CpG island 1 of *MGAT3* induced no increase of gene transcription. The human *IL6ST* locus (A) and *MGAT3* locus (B) are shown with positions of the pyrosequencing assays (A1, A2) indicated by yellow rectangles. Magnified insets show individual CpG sites (white circles) targeted by dCas9 fusions and subsequently analyzed for methylation level. Arrows, aligned to the magnified regions, indicate 20 bp binding sites of gRNAs used to guide the dCas9-fusions. Arrows point toward the PAM sequence. Annotated promoter is represented by shaded red triangles; TSS, transcription start site (kinked arrow); A, dCas9 fusions with active catalytic domain; I, dCas9 fusions with inactive catalytic domain; NT, active dCas9 fusions with non-targeting gRNA. *** *P* < 0.001.

To demonstrate that two different dCas9 fusions with antagonistic activities can operate simultaneously within the same cell, we chose candidate genes in the context of studies of IgG glycosylation and chronic inflammatory diseases (29): *BACH2*, the key transcription factor important for B-lymphocyte maturation and differentiation (37); *IL6ST*, signal transducer for many cytokines; *MGAT3*, a mannosyl(β-1,4-)-glycoprotein β-1,4-N-acetylglucosaminyltransferase, responsible for addition of bisecting GlcNAc to the three-mannose core of *N*-glycan structures and involved in inflammation (38); and *HNF1A*, a transcription factor, a master regulator of protein fucosylation (39). We used our new CRISPR/dCas9 modular toolbox to manipulate gene pairs *BACH2*–*HNF1A* and *IL6ST* –*MGAT3*, where we targeted *BACH2* and *IL6ST* with DNMT3A-dSpCas9 for hypermethylation, and *HNF1A* and *MGAT3* with TET1-dSaCas9 for demethylation. The candidate loci were either almost completely unmethylated (*BACH2, IL6ST*) or highly methylated (*HNF1A, MGAT3*) in untransfected HEK293 cells. By using two fluorescent markers, we confirmed that the bulk of the cells were co-transfected with both dCas9 fusions. Of the 257 cells analyzed, 59.1% were positive for both fluorescent signals, while 30% were positive only for red mRuby3 and 10.9% only for green mClover3 fluorescence signal. Since fluorescent signals originated from the same transcripts that directed synthesis of dCas9 orthologues with effector domains (fluorescent proteins were linked by 2A-type self-cleaving peptides in frame with dCas9), the yellow fluorescent signal indicated that both dCas9 fusions entered the same cell. In addition, this showed that the bulk of antagonistic activities (methylation versus demethylation) co-occurred in doubly transfected cells and that there is no interference of the two different effector domains that were directed by dSpCas9 and dSaCas9 orthologs and their cognate gRNAs (Figures 4 and 5). Changes in methylation level ranged from 20% to 80% compared to mock transfected cells, in the opposite direction for each locus within a pair (methylation versus demethylation). The level of methylation change was specific for each CpG site, but the methylation profiles for all analyzed CpG sites were consistent across experiments. We were interested if imposed CpG methylation and demethylation have an effect on transcript level of the targeted genes. Therefore, gene activity was always measured 8th day following transfection, based on the results of the time-course experiment, which had showed that gene expression profiles (fold change) closely followed CpG methylation profiles (% methylation change) for both dCas9-fusion with DNMT3A (27) and TET1 (Supplementary Figure S6).

The most pronounced effect of altered CpG methylation on RNA transcripts level was observed for the *HNF1A* gene where we targeted four CpG dinucleotides (Figure 4B) for which we have had previous indication to had putative regulatory role from a correlation study (40). The TET1-dSaCas9 fusion decreased methylation level 27%, 32%, 49% and 65% at CpG1, CpG2, CpG3 and CpG4, respectively, which was associated with 5.614-fold change (*P* = 0.0005 to inactive control, I; *P* = 0.0012 to non-targeting control, NT) of RNA transcript level (Figure 4C). Its gene partner in pair, *BACH2*, was at the same time targeted with DNMT3A-dSpCas9 using a multi-guide module comprising four different gRNAs (Figure 4A); randomly chosen 27 CpG sites located within CpG island (some of them positioned near transcription start site, TSS) were targeted. The methylation level change varied from 2–5% to maximally 55%, depending on the CpG site. However, despite the wider region of the *BACH2* promoter was methylated using DNMT3A-dSpCas9 the associated fold change of the gene transcription was modest (0.568-fold change, *P* = 0.0003 to I; *P* = 0.0012 to NT). In case of the *MGAT3*–*IL6ST* gene pair, randomly chosen CpG sites positioned within CpG islands of these gene promoters were manipulated using the multi-guide system containing four gRNAs for *IL6ST* targeting and six gRNAs for *MGAT3* targeting (Figure 5). The *IL6ST* region of choice, comprising 15 randomly chosen CpG sites, was targeted using DNMT3A-dSpCas9 and methylation change varied from 3% to 29% depending on an individual CpG site. The TET1-dSaCas9 fusion was used in the same experiment for targeting 14 randomly chosen CpG sites dispersed within CpG island 1 of the *MGAT3* gene. Corresponding decrease in methylation level varied between 19% and 72% depending on an individual CpG site (Figure 5). The externally introduced hypermethylation in the *IL6ST* promoter was followed by modest decrease (0.583-fold change; *P* = 0.0002 to I, *P* < 0.0001 to NT) of transcriptional activity, while induced hypomethylation of *MGAT3* did not associate with increased gene transcript level (0.671-fold change; *P* = 0.06 to I). Furthermore, construct with an inactive TET1 catalytic domain (negative control) also decreased *MGAT3* gene expression level, possibly due to CRISPR interference.

### Simultaneous epigenetic manipulation of the *MGAT3* and *HNF1A* genes using DNMT3A-dSpCas9 and TET1-dSaCas9 induced changes in the *N*-glycome of BG1 cells

**Figure 6.**
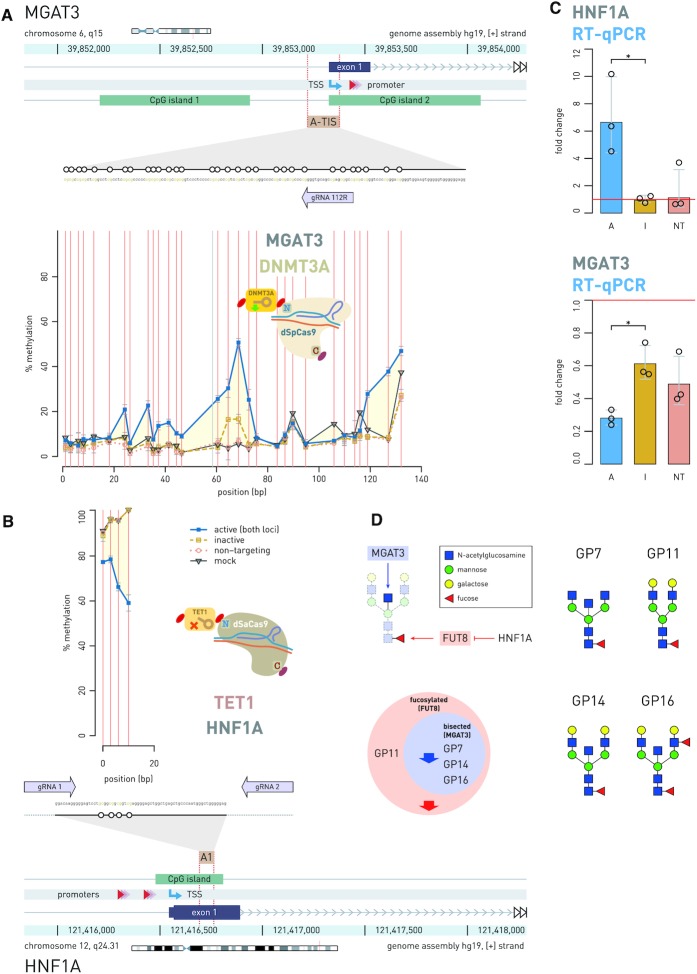
Simultaneous epigenetic manipulation of the *MGAT3* and *HNF1A* loci using DNMT3A-dSpCas9 and TET1-dSaCas9 showed an effect on total N-glycome in BG1 cells. (**A**) Thirty CpG sites within 200 bp wide putative regulatory region, residing in CpG island 2 of the *MGAT3* promoter and centered about 100 bp upstream from the TSS, were targeted with DNMT3A-dSpCas9 using a single gRNA. (**B**) At the same time, four regulatory CpG sites in the first exon of the *HNF1A* promoter was targeted with TET1-dSaCas9 using two gRNAs. (**C**) Induced hyper- and hypo-methylation resulted in significant change in gene transcript level: *MGAT3* showed 0.28-fold change (*P* = 0.004) and *HNF1A* showed 6.64-fold change (*P* = 0.004). A, dCas9 fusions with active catalytic domain; I, dCas9 fusions with inactive catalytic domain; NT, active dCas9 fusions with non-targeting gRNA. (**D**) The *MGAT3* gene downregulation led to a statistically significant decrease (*P* = 0.005 compared to inactive DNMT3A-dSpCas9; *P* = 0.032 compared to mock-transfected cells) of the glycan structures with bisecting GlcNAc (GP7, GP14 and GP16). The *HNF1A* gene upregulation resulted in decrease (*P* = 0.005 compared to inactive TET1-dSaCas9, *P* = 0.028 to mock-transfected cells) of core fucose in the glycan structures with and without bisecting *N*-GlcNAc, probably through a known effect of *HNF1A* on the fucosyltransferase FUT8. Effects of *HNF1A* on fucosylation were represented by the sum of abundance of all complex core-fucosylated structures that are products of FUT8: GP7, GP11, GP14, GP16. Analogously, the effect of GNT-III enzyme, encoded by *MGAT3*, was represented by the sum of abundance of all structures containing bisecting *N*-GlcNAc: GP7, GP14, GP16. Thick blue and red arrows on the Venn diagram show the direction of the glycan change (a decrease of certain glycan structures in the total *N*-glycome of BG1 cells). * *P* < 0.05.

The regulatory impact of DNA methylation on *MGAT3* gene expression was previously shown for several ovarian cancer cell lines, including the BG1 cell line. Furthermore, *MGAT3* gene expression in these cell lines correlates with the presence of N-glycan structures with bisecting GlcNAc (41). Because we had previous knowledge about putative regulatory CpG sites within the *MGAT3* promoter and the effect of DNA methylation and expression of this gene on the glycan phenotype of BG1 cells, we went for epigenetic manipulation of the *MGAT3* promoter in this cell line. Since the TSS region of the *MGAT3* promoter is unmethylated and the gene is expressed in BG1 cells, we targeted 30 CpG sites, located within 200 bp wide putative regulatory region (41) in CpG island 2, and centered about 100 bp upstream from the TSS, with DNMT3A-dSpCas9 using a single gRNA (Figure 6A). At the same time, we targeted four regulatory CpG sites in the *HNF1A* promoter with TET1-dSaCas9 using two gRNAs, because we had confirmed in HEK293 cells that epigenetic manipulation of these CpGs affected gene transcription. We checked the efficacy of double transfection by detection of fluorescent markers—out of 173 cells analyzed, 84.4% were positive for yellow color (showing of co-localization of two fluorescent proteins), while 8.1% were positive only for red mRuby3 and 7.5% only for green mClover3 fluorescence signal. Altered methylation level led to changes in expression, with *HNF1A* upregulated 6.64-fold and *MGAT3* downregulated to 0.28-fold expression level of mock-transfected cells (Figure 6C). To investigate the effect of simultaneous *MGAT3* downregulation and *HNF1A* upregulation on the glycan phenotype, we analyzed total *N*-glycome of BG1 cells (Supplementary Figure S7 and Supplementary Table S6). We detected changes in glycan structures with core fucose and bisecting GlcNAc (Figure 6D). Direct effect of downregulated GNT-III enzyme (encoded by *MGAT3*), responsible for the addition of *N*-acetylglucosamine to the three-mannose core of glycan structures, led to a statistically significant decrease (*P* = 0.0050 compared to inactive DNMT3A-dSpCas9; *P* = 0.0324 compared to mock-transfected cells) in structures with bisecting *N*-GlcNAc (glycan peaks GP7, GP14 and GP16; Figure 6D, and Supplementary Figure S7). Upregulation of *HNF1A*, probably via its known regulatory role on the FUT8 enzyme (39), led to a statistically significant decrease in complex glycan structures with core-fucose (GP7, GP11, GP14 and GP16; *P* = 0.0053 compared to inactive TET1-dSaCas9, *P* = 0.0281 compared to mock-transfected cells) where GP11 is a fucosylated glycan structure without a bisecting GlcNAc (Figure 6D and Supplementary Figure S7). The decrease in GP11 alone was statistically significant (*P* = 0.0122 compared to inactive TET1-dSaCas9, *P* = 0.0161 compared to mock-transfected cells).

### Time course of simultaneous manipulation of the *HNF1A* regulatory region using the VPR-dSpCas9 and TET1-dSaCas9 fusions

**Figure 7.**
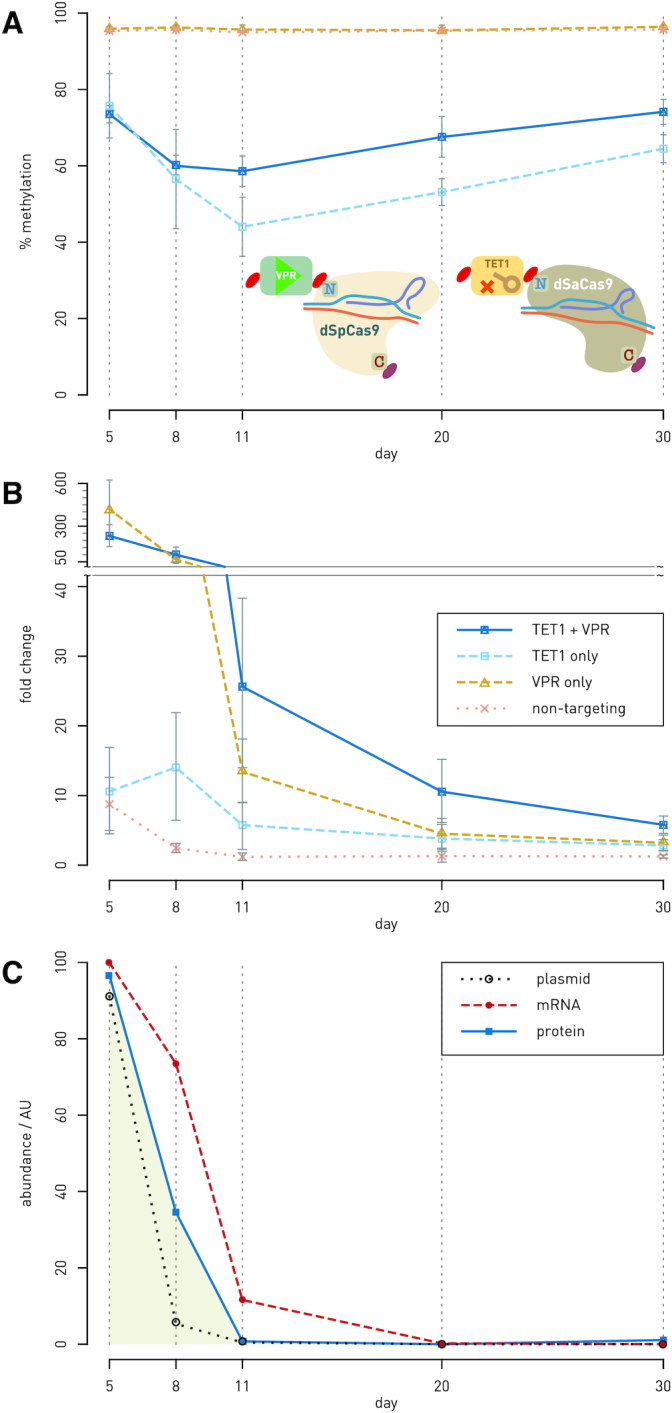
Time-course experiment of simultaneous manipulation of the *HNF1A* regulatory region using VPR-dSpCas9 and TET1-dSaCas9. (**A** and **B**) The *HNF1A* regulatory region was targeted with VPR-dSpCas9 for gene activation and TET1-dSaCas9 for targeted CpG demethylation, either using individual dCas9-fusions or both dCas9 fusions at the same time in HEK293 cells. Methylation level at four regulatory CpG sites (**A**) as well as gene transcript level (**B**) were monitored for 30 days following transfection. Synergistic effect of the VPR-dSpCas9 and TET1-dSaCas9 fusions on gene transcription level was achieved already on 11th day following transfection: the increase in gene transcript level was 26 ± 12-fold change when compared to 13 ± 4-fold change using only VPR-dSpCas9 (*P* = 0.017). The synergistic effect persisted until 20th and 30th day of gene expression monitoring (*P* = 0.004; *P* = 0.007). Percentage of methylation was shown as average methylation level on four regulatory CpG sites. (**C**) Time course of the level of plasmid encoding TET1-dSaCas9, transcribed mRNA and the expressed dCas9 fusion protein in HEK293 cells after transfection. Plasmid and RNA were quantitated by RT-qPCR, with their abundance calculated from delta-*C*_t_ relative to internal controls (*RAG1* for plasmid DNA and *GAPDH* for transcript level). Level of the fusion protein TET1-dSaCas9 was determined by quantitation of signals obtained by western blotting (pictures of membranes are given as Supplementary Figure S9A). Abundance of the TET1-dSaCas9 was normalized to β-tubulin for each lane. No protein could be detected by western blotting at day 11 and later.

Results of the previous experiments of targeted demethylation of four CpG sites, located in the first exon near TSS in *HNF1A* gene, using the TET1-dSaCas9 fusion in HEK293 and BG1 cells, confirmed that these CpGs were regulatory, since the gene transcript level significantly changed following the epigenetic manipulation. Next, we compared the effects of using TET1-dSaCas9, VPR-dSpCas9 (for direct gene activation) and simultaneous transfection of both fusions in modulating *HNF1A* transcriptional expression. We conducted single and double transfections in HEK293 cells using these two dCas9 fusions and monitored CpG methylation and gene transcript levels over a period of 30 days. If we observe the effect of the dCas9-fusions used either together or individually on CpG methylation only, the TET1-dSaCas9 fusion used alone induced a greater decrease of methylation level than if the fusions were used together (Figure 7A), and this could be explained by possible interference between the two effector domains (i.e. VPR and TET1). In terms of *HNF1A* transcriptional expression, direct activation by VPR-dSpCas9 alone induced high level of expression on the 5th day following transfection (411 ± 209-fold change). This effect however was reduced by the 8th day (62 ± 35-fold change) post-transfection. However, simultaneous targeting of the VPR-dSpCas9 and TET1-dSaCas9 fusions to the four CpG sites induced persistent upregulation of *HNF1A*, exceeding the effect of VPR only from 11th day post-transfection, and persisting until the 30th day following transfection (Figure 7B). On the 11th, 20th and 30th days, respectively, the VPR-dSpCas9 and TET1-dSaCas9 fusions together induced 26 ± 12, 11 ± 5 and 6 ± 1-fold change, while the VPR-dSpCas9 alone induced 13 ± 4, 5 ± 2 and 3 ± 1-fold change (*P* = 0.017, *P* = 0.004 and *P* = 0.007, respectively). Furthermore, to determine whether the stability of demethylation through time is due to the inheritance of induced epigenetic change or due to persistent expression and activity of the fusion proteins, we checked for the presence of TET1-dSaCas9 at DNA (plasmid DNA), RNA (transcript) and protein levels. The amount of TET1-dSaCas9 at DNA and RNA level decreased sharply on 11th day after the transfection while the presence of TET1-dSaCas9 protein was undetectable from 11th day after transfection (Figure 7C and Supplementary Figure S9A).

### Whole-genome methylation analysis reveals reduced off-target effect when DNMT3A- and TET1-dCas9 fusions are expressed under a weak promoter

Recent reports indicate that exogenous expression of dCas9 fusions with different DNA methyltransferases often induces extensive off-target DNA methylation (for a review, see Tadić *et al.* (42) and references therein), underscoring the need for further improvements. Therefore, we aimed to assess the specificity of our modular dCas9-based system and to investigate the effect of modulating fusion protein expression relative to selection marker expression on off-target dCas9 activity. The DNMT3A-dSpCas9 fusions were targeted to *IL6ST*, and TET1-dSpCas9 fusions were targeted to *MGAT3*. Two versions of each DNMT3A-dSpCas9 and TET1-dSpCas9 fusions were designed: (i) a version in which the effector proteins (dCas9 in fusion with either DNMT3A or TET1) and the puromycin-resistance selection marker were driven under the same strong, constitutive CBh promoter (denoted as the ‘primary cassette’) and (ii) a version in which the effector proteins were expressed under the weak EFS promoter, with independent SV40-driven expression of puromycin resistance selection marker (denoted as the ‘secondary cassette’). Stronger expression of dCas9 fusions in the ‘primary cassette’ compared to much weaker expression in the ‘secondary cassette’ was confirmed by western blotting (Supplementary Figure S9B).

**Figure 8.**
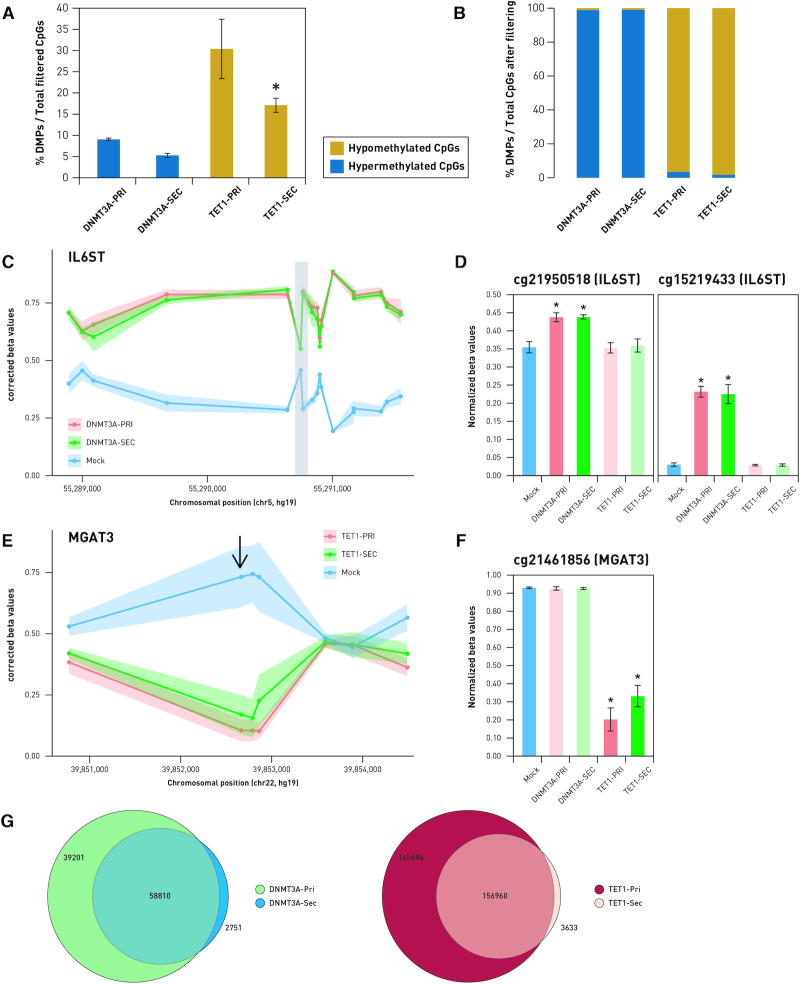
Analysis of whole-genome methylation following epigenetic modulation of the *IL6ST*/*MGAT3* promoters using DNMT3A-dSpCas9 and TET1-dSpCas9 fusion constructs. (**A**) Fraction of differentially methylated probes compared to mock controls against total filtered probes analyzed on the Infinium MethylationEPIC array. Labels DNMT3A-PRI/TET1-PRI and DNMT3A-SEC/TET1-SEC indicate ‘primary’ and ‘secondary cassette’, respectively. (**B**) Fraction of differentially methylated probes, which were hypermethylated or hypomethylated, compared to mock controls in cells transfected with DNMT3A-dSpCas9 and TET1-dSpCas9 fusions. (**C** and **E**) Dots represent CpG sites targeted by DNMT3A-dSpCas9 and TET1-dSpCas9 fusion constructs. Lines show pattern of methylation (C) and demethylation (E) change at CpG island of *IL6ST* and *MGAT3* genes. Comparison of CpG methylation patterns induced by DNMT3A-dSpCas9 expressed under strong (‘primary cassette’) or weak (‘secondary cassette’) promoter on probes located 2500 bp up- and downstream of the targeted region (shaded in gray) of *IL6ST* is shown on (C). Changes to β-values in the two probes lying within this region are illustrated on (**D**). (**E**) Comparison of DNA methylation patterns induced by TET1-dSpCas9 primary and secondary cassettes 2500 bp up- and downstream of the targeted region of the *MGAT3* promoter region. The probe cg21461856 is the only one lying within this region (indicated with the arrow). Changes to β-values in cg21461856 are illustrated in the graph (**F**). (**G**) Venn diagrams illustrating overlap between DMPs induced by the primary and secondary cassettes of DNMT3A-dSpCas9 and TET1-dSpCas9 fusions. * *P* < 0.05.

To assess DNA methylome-wide on- and off-target effects, each transfection condition was analyzed using Illumina Infinium MethylationEPIC BeadChip arrays (see Supplementary Figure S10, Supplementary Tables S7 and S8 for quality control). We observed that expressing dSpCas9-fused effector proteins under the ‘secondary cassette’ reduced off-target DNA methylation without a significant reduction in on-target effects. The introduction of the DNMT3A-dSpCas9 expressed in the ‘primary cassette’ resulted in 71 910 ± 2367 differentially methylated probes (DMPs) (with the cut-off for significance set as FDR-adjusted *P-*values < 0.05 and effect size > 5%), accounting for 9.1% ± 0.3 of all filtered probes (Figure 8A and G). The DNMT3A-dSpCas9 fusion expressed in the ‘secondary cassette’ reduced the average number of DMPs to 41 894 ± 3939 probes, accounting for 5.3% ± 0.5 of all filtered probes, though the difference between the ‘primary’ and ‘secondary cassettes’ was not significant when tested by Student–Newman–Keuls (SNK) post-ANOVA multiple comparison tests. The TET1-dSpCas9 fusion expressed under the ‘primary cassette’ induced differential methylation in 240 991 ± 55 452 probes accounting for 30.4% ± 6.99 of filtered probes, while the TET1-dSpCas9 expressed under the secondary cassette significantly reduced differential methylation in 135 697 ± 13 079 probes, accounting for 17.1% ± 1.65 of the filtered probes (*P* = 0.022 by SNK post-ANOVA multiple comparison test). As expected, 98.7% of the DMPs induced using the DNMT3A-dSpCas9 fusions with either ‘primary’ or ‘secondary cassettes’ were hypermethylated, whereas 97.2% and 93.2% of the reported DMPs were hypomethylated using TET1-dSpCas9 with either ‘primary’ or ‘secondary cassettes’, respectively (Figure 8B). Interestingly, the DMPs induced by the ‘primary’ and ‘secondary’ cassettes of the DNMT3A and TET1 fusion proteins were largely overlapping (Figure 8G), suggesting that off-target fusion protein activity occurs in a non-stochastic manner.

To confirm the on-target activity of the dCas9-based modular system, methylation in the targeted regions of the *IL6ST* and *MGAT3* loci was analyzed in DNMT3A-dSpCas9 and TET1-dSpCas9 samples (Figure 8C and E). The Infinium MethylationEPIC array contains two probes in the targeted *IL6ST* region (Figure 8D) and one probe in the targeted *MGAT3* region (Figure 8F). Expression of DNMT3A-dSpCas9 from both ‘primary’ and ‘secondary cassettes’ induced significant hypermethylation in the probes cg21950518 and cg15219433, whereas TET1-dSpCas9 expressed from either ‘primary’ or ‘secondary cassettes’ induced significant hypomethylation in the probe cg21461856. The targeted probes of DNMT3A-dSpCas9 were not affected by TET1-dSpCas9 and vice versa (Figure 8D and F). Interestingly, we observed that hypermethylation is induced to a greater extent at CpG sites flanking the targeted region (±2500 bp) in DNMT3A-dSpCas9 samples (Figure 8C). In contrast, hypomethylation effect by TET1-dSpCas9 was strongest at the target sequences and *cis*-sites, with less pronounced effects observed at the relatively distal flanking regions (±2500 bp) (Figure 8E). It should be noted that to allow clearer visualization, Figure 8C and E was plotted using methylation (β) values that were adjusted by Surrogate Variable Analysis (SVA) and log-transformed into *M*-values, which do not directly reflect the real biological % DNA methylation. Similar graphs depicting actual methylation values (unadjusted betas normalized by the R package ‘funnorm’) are included in Supplementary Figure S11.

## DISCUSSION

Various platforms and protein scaffold systems such as SunTag, SAM and MoonTag have been developed for direct transcriptional modulation (14,22,43–46). Also, individual fusions of dCas9 to various epigenetic writers and erasers are available for targeted changes of epigenetic marks. Here, we have created and validated a CRISPR/dCas9-based modular system that can be efficiently assembled and easily reconfigured for a specific experimental setup. This system represents a complete cloning platform, to which various functional modules can be added easily. It has the possibility to exchange Cas9 orthologs, effector domains and selection markers as well as to be combined with a multi-guide system, which ensures simultaneous delivery of up to six gRNAs into the same cell. We validated the modular CRISPR/dCas9-based toolbox in multiple configurations regarding fusions to either C- or N-terminus of a dCas9 protein as well as use of different orthologous dCas9 (from either *S. pyogenes* or *S. aureus*). The system based on direct fusions between a dCas9 protein and an effector domain has the minimal number of components that need to be delivered on separate vectors, making experimental design simpler and more robust compared to other systems where dCas9 and effector domains are expressed separately, and the proteins need to form an active complex within the cell (for an overview, see Tadić *et al.* (42) and references therein). Further, a multi-guide system can be fully integrated, facilitating multiple targeting with up to six gRNAs. Finally, different dCas9 orthologs show no interference when multiple functionalities are independently targeted to different loci.

Recently, Gao *et al.* designed a flexible dCas9-based platform for inducible orthogonal gene regulation (12). Their platform uses the dCas9 protein fused with KRAB and VPR effector domains and shows robust function in these simple fusions in direct gene repression/activation within mammalian cells (12). On the other hand, activity of the DNMT3A and TET1 catalytic domains critically depends on the configuration of the fusion (N-terminal versus C-terminal), the type of the linker and/or on NLS availability, which we have demonstrated in this work. While fusions of DNMT3A and TET1 with dCas9 have already been described (23–28), there was no universal and robust approach for creating active fusions of DNMT3A and TET1 with different dCas9 orthologs, which would work within the same cell simultaneously and perform antagonistic activities (i.e. DNA methylation and demethylation). In order to create a modular system capable of antagonistic and/or synergistic activities, we tested both C- and N-terminal configuration between dCas9 orthologs (*S. pyogenes* and *S. aureus*) and DNMT3A or TET1 catalytic domains for activity in two human cell lines. We consistently observed more robust activity of both DNMT3A and TET1 catalytic domains when fused N-terminally to either dSpCas9 or dSaCas9. Although C-terminal fusions to dCas9 from *S. pyogenes* performed satisfactorily when proper targeting to the nucleus was assured by an additional NLS nucleoplasmin (NLS-NP) at the C-terminus of the catalytic domain, we chose N-terminal fusions as a streamlined and universal approach. On the other hand, C-terminal fusions of catalytic domains with dCas9 from *S. aureus* suffered poor performance even when properly targeted to the nucleus by addition of NLS-NP to the both sides of the construct (Figure 2) or when different linkers were used in fusions (Supplementary Table S4). Though the Cas9 from *S. aureus* is smaller and has improved level of specificity in comparison with Cas9 from *S. pyogenes*, and both SpCas9 and SaCas9 show similar kinetics and activity as nucleases (47), in our hands dSaCas9 C-terminal fusions did not work either for DNMT3A or TET1 catalytic domains. This lack of activity may be due to steric hindrances between dSaCas9 and the effector domains or to misfolding and degradation of the fusion proteins.

The activity profiles of C- and N-terminal fusions of dSpCas9 with both DNMT3A and TET1 appeared quite similar, with similar position of peak activity relative to the gRNA-binding site. Also, N-terminal fusions of DNMT3A and TET1 to dSaCas9 were similar in activity profile to all dSpCas9 C- and N-terminal fusions with DNMT3A and TET1 effector domains. This shows that, at the scale of the effector domain interaction with DNA, configuration of the fusion construct (C- or N-terminal) does not significantly contribute to the activity profile, although the effect of fusions with C-terminal effector domains was mainly concentrated downstream from the binding site, while N-terminal domains demonstrated a strong upstream activity as well (Figure 1). The activity of all dCas9 N-terminal fusions could be detected on both sides of the gRNA-binding site. The additional activity peaks, reproducibly detected further upstream or downstream (180–200 bp) from the gRNA-binding site, were consistent with possible contact of effector domains with DNA on adjacent nucleosomes. When comparing the activity of N-terminally fused effector domains to either dSaCas9 or dSpCas9, both orthologs performed satisfactorily, though dSpCas9 fusions consistently showed stronger activity (Figure 1; top panel).

We also demonstrated the application of this molecular toolbox to simultaneous targeting of several pairs of gene loci using fusions of dCas9 with effector domains conferring antagonistic or synergistic activities, at the same time within the same cells. We epigenetically manipulated *BACH2*–*HNF1A* and *IL6ST*–*MGAT3* gene pairs in HEK293 cells, where we targeted *BACH2* and *IL6ST* with DNMT3A-dSpCas9 fusion and *HNF1A* and *MGAT3* with TET1-dSaCas9 fusion. As expected, changes in cytosine methylation went in the opposite direction for each locus within a pair (methylation versus demethylation) as a result of manipulation of the two dCas9 fusions with antagonistic activities. Imposed change of cytosine methylation was shown to change transcription level of the manipulated genes at different scale, depending on the region within the promoter that we targeted with a dCas9 fusion. The effect on the transcript level of the *BACH2* and *IL6ST* genes was moderate (0.568-fold change, *P* = 0.0003 and 0.583; *P* = 0.002, respectively), although wide regions spanning about 90 bp encompassing 27 CpGs (for *BACH2*) to 15 CpGs (for *IL6ST*) were targeted using DNMT3A-dSpCas9 (Figures 4 and 5). On the other hand, TET1-dSaCas9 targeted demethylation of only four CpG sites, located in the first exon of the *HNF1A* gene strongly influenced the expression level of this gene (5.614-fold change; *P* = 0.0012). In contrast, TET1-dSaCas9 induced demethylation at 14 CpG sites located in the first CpG island 1 of *MGAT3* promoter did not increase transcription level of this gene in HEK293 cells. However, when we targeted the DNMT3A-dSpCas9 fusion to 30 CpG sites positioned near TIS region in the CpG island 2 of the *MGAT3* promoter, which was previously suggested as putative regulatory region in several ovarian cell lines including BG1 (41), we achieved considerable effect on *MGAT3* transcription (0.28-fold change, *P* = 0.004) (Figure 6C). Although the same CpG sites are unmethylated in the HEK293 cell line, expression level of the *MGAT3* gene is low. Therefore, we reasoned that we could not observe any effect of externally imposed hypomethylation on *MGAT3* expression level in HEK293 cells, which suggests that other factors besides CpG methylation are involved in regulation of *MGAT3* expression and that relevant regulatory CpG sites are probably cell line specific.

Further, we manipulated the same CpG sites in another cell line, BG1, using TET1-dSaCas9, and again achieved an increase of the transcript level (6.64-fold change, *P* = 0.004). At the same time *MGAT3* gene was manipulated in same cells, since we had information about putative regulatory region within the *MGAT3* promoter, too (41). Both genes are involved in protein *N*-glycosylation: *MGAT3* is a classical glyco-gene coding for glycosyltransferase, which produces *N*-glycan structures with bisecting *N*-GlcNAc, while *HNF1A* is a master regulator of both core and antennary fucosylation of *N*-glycan structures (39). In addition, the relationship between cytosine methylation in CpG island 2 of the *MGAT3* gene promoter, its expression and the presence of bisecting GlcNAc on N-glycans in ovarian cancer cell lines were already demonstrated, although the methylation change was obtained using 5-aza-deoxycitidine treatment (41). Here, we targeted 30 putative regulatory CpG sites, centered about 100 bp upstream from the TSS, with DNMT3A-dSpCas9 and downregulated *MGAT3* to 0.28-fold change following epigenetic manipulation. In addition, simultaneous epigenetic manipulation of the *MGAT3* and *HNF1A* genes resulted in a decrease of *N*-glycan structures with bisecting GlcNAc (the result of *MGAT*3 downregulation) and a decrease of complex glycan structures with core-fucose (an indirect effect of *HNF1A* upregulation). The effect of *HNF1A* on several fucosyltransferases, such as FUT3, FUT5, FUT6, FUT7, FUT8, FUT9, FUT10 and FUT11, has been previously demonstrated by RNA interference, where *HNF1A* appeared to downregulate FUT8 (39), an enzyme that adds a fucose on the three-mannose core of N-glycan structures.

Our results demonstrate that proper targeting of functional CpG sites or regulatory region is necessary to obtain an effect on gene transcription. Therefore, the difference in the effect of externally induced hyper- and hypomethylation we achieved on transcript level of the candidate genes was due to the relevance of the targeted CpG sites for transcriptional regulation. In our earlier correlation study between CpG methylation and gene expression in different cell lines, we identified four putative regulatory CpG sites in the first exon of the *HNF1A* gene (40). In the present study, using dCas9 fusions we confirmed unambiguously that cytosine methylation at these sites plays a regulatory role, because it significantly altered *HNF1A* transcript level. Therefore, as few as four adjacent CpG sites spanning a 13 bp short region can regulate transcription. Similar effect was reported for the *Ascl1* locus when TALE fusions with TET1 or DNMT3A were used (48). However, although Lo *et al.* (48) searched for putative transcription factor binding sites that included the manipulated CpG dinucleotides, the achieved influence on *Ascl1* gene expression was relatively weak (1.58-fold increase in CpG methylation that was associated with a 1.75-fold decrease in gene expression) and the level of regulation was in line with the results observed when targeting random CpG sites scattered throughout a wider region within the *BACH2* and *IL6ST* promoters in our study. It is also worth noting that we deliberately selected almost fully methylated or unmethylated regions for targeted manipulation, which was done in order to make any induced changes in expression obvious. However, it is still not clear in terms of dose–response how DNA methylation affects gene expression. In our earlier work, we observed that multiple targeting by co-expression of several gRNAs can have a synergistic effect on site-specific CpG methylation (27). This knowledge proved valuable in case of *BACH2* and *IL6ST*, where DNMT3A-dSpCas9 in combination with multi-guide system was used for a greater increase of methylation levels at the targeted regions. This is particularly useful when we do not have previous knowledge of functional CpG sites, because methylation of a wide region tends to spread along a CpG island and eventually reaches the several CpG sites directly responsible for transcriptional control. In this case, CpG methylation change probably induces changes in chromatin conformation and DNA accessibility, triggering multiple processes such as histone modifications and nucleosome remodeling (49).

We were also interested in the effect on gene expression from simultaneous targeting of the VPR-dSpCas9 (for direct gene activation) and TET1-dSaCas9 fusions (for hypomethylation to demethylation) to the *HNF1A* regulatory region. Therefore, we double transfected HEK293 cells using these two dCas9 fusions and monitored CpG methylation and gene transcript levels during 30 days. Although direct gene activation showed a stronger effect on transcription than the combined VPR/TET1 activation on the 5th day after transfection, the effects of the VPR-dSpCas9 fusion was relatively transient, and was surpassed by the VPR/TET1 combination after 11th day post-transfection, and *HNF1A* upregulation persisted until the 30th day (Figure 7B). If we observe the effect of the dCas9 fusions used either together or individually on CpG methylation only, the TET1-dSaCas9 fusion used alone induced a greater reduction of CpG methylation level than if the fusions were used together, and this was expected because the binding of VPR-dSpCas9 possibly interfered with binding of the TET1-dSaCas9 construct. In our previous and present work, we demonstrated that externally induced hyper- and hypo-methylated state at targeted CpG sites, using simple C-terminal fusions dSpCas9-DNMT3A (27) and dSpCas9-TET1 (Supplementary Figure S1), persisted through mitotic divisions, reaching the maximum level 7–9 days after transfection and persisting for at least 15 days. While the initial change in methylation level was rapid, the return to the baseline was gradual, with a detectable change (around 20% in average) persisting for up to 30 days after transfection. In this study, we performed a time-course experiment targeting the *IL6ST* and *HNF1A* genes independently, using the N-terminal fusions DNMT3A-dSpCas9 and TET1-dSaCas9. These two fusions were constructed using the new modular approach. During 30 days of monitoring CpG methylation, we found that the effect of methylation and demethylation remained stable for a longer time than in case when we used C-terminal fusions were used. Methylation levels stay altered up to 30% to 40% even 30 days after transfection of HEK293 cells compared to initial level of methylation (mock) at the targeted CpG sites within the promoters of *IL6ST* and *HNF1A* (Supplementary Figure S8). Furthermore, we found that almost no plasmid was present on 8th day after the transfection, while the dCas9 protein was undetectable after 11th day following transfection and any lingering mRNA expression extinguished by day 20 after cell transfection (Figure 7C and Supplementary Figure S9A). Therefore, we can be fairly confident that around day 11 there was no significant new demethylation induced by TET1-dSaCas9 fusion construct. Yet, the effect on CpG methylation persisted until the day 30 after transfection (Figure 7A and Supplementary Figure S8). A possible explanation for the long-lasting effect on cytosine methylation (especially compared to the earlier experiments involving C-terminal fusion dSpCas9-DNMT3A) is the larger initial effect achieved by the new tools on DNA methylation levels, which might have activated other epigenetic mechanisms (such as histone modifications), which subsequently consolidated and reinforced the induced epigenetic changes. In addition, somewhat higher activity of N-terminal fusions might be explained by the additional NLS compared to C-terminal fusions: while the N-terminal fusion has exposed NLS at both termini, the C-terminal fusion has an exposed NLS only at the N-terminus of dCas9 portion of fusion construct (see schematics in Figure 1). Targeting a wider region using the multi-guide system may have contributed to an increased overall effect on methylation. Similarly, Liu *et al.* demonstrated that the effect of demethylation induced by dCas9-TET1 lasted for 14 days, and restored *FMR1* gene expression during that time in the absence of constitutive presence of dCas9-TET1, suggesting sustained gene reactivation through cell divisions (22). It would be interesting to analyze how forced demethylation and imposed methylation affect other epigenetic mechanisms, notably histone modifications, at the short region of *HNF1A* targeted by TET1-dSaCas9, in comparison with larger region of *IL6ST* promoter targeted by DNMT3A-dSpCas9, and whether those other modifications contribute to long-lasting change of CpG methylation that we observed in the time-course experiments.

In contrast to applications of Cas9 for generating knockouts, where every productive binding to genomic DNA has a potential to introduce a mutation, and where strategies for avoiding off-target effects focus on achieving high fidelity of Cas9/DNA interaction (50), the problem with epigenome editing tools is fundamentally different (for a review, see Tadić *et al.* (42) and references therein). Here, passing interactions will leave only a minimal mark; more importantly, the bulk of unspecific activity does not come from unspecific Cas9/DNA interactions, but from unguided activity of the linked catalytic domains, such as DNMT3A and TET1. Indeed, Galonska *et al.* (51) demonstrated that the dCas9-DNMT3A fusion induced global increase in CpG methylation without gRNA and that the off-target activity was mostly random and remained unchanged even when using single or multiple gRNAs. In an attempt to mitigate the off-target activity, we opted for down-regulating expression of the dCas9 fusion construct by placing it under a weaker promoter, with the aim to achieve a more favorable on-target to off-target ratio. The selection marker expression (puromycin resistance) was driven by a strong promoter in a separate expression cassette on the backbone plasmid, thus ensuring efficient selection. This ‘secondary cassette’ approach proved successful, as evidenced by whole-genome methylation analysis, which showed decreased off-target activity with practically unaffected on-target activity when dSpCas9 N-terminal fusions with DNMT3A and TET1 were put under a weaker promoter. Whole-genome methylation analysis using the Illumina 850K platform also revealed details about functioning of our new and improved dCas9-based tools for targeted CpG methylation and demethylation. We have demonstrated that reducing dCas9 fusion protein expression relative to the selection marker can substantially reduce off-target activity. This complements the findings of a separate study, which demonstrates that modulating DNMT3A expression relative to dCas9 results in a reduction in off-target DNA methylation while maintaining high on-target DNA methylation (52). However, it should be noted that while our approach has reduced off-target hypomethylation induced by TET1-dSpCas9, further improvements need to be made before the TET1-dSpCas9 fusion can be considered reliable and used as a specific tool for inducing site-specific hypomethylation. Interestingly, the DMPs induced by the ‘primary’ and ‘secondary’ cassettes of the DNMT3A-dSpCas9 and TET1-dSpCas9 fusion proteins were largely overlapping (Figure 8G), suggesting that off-target activity occur in a non-stochastic manner. Annotation of DMPs by their relationship to CpG islands indicated that hypermethylation induced by DNMT3A-dSpCas9 was primarily enriched in the CpG-rich CpG island-to-shore regions (Supplementary Figure S12A) while the hypomethylation induced by TET1-dSpCas9 occurred mostly at the less dense CpG shore-to-shelf regions (Supplementary Figure S12B). The reverse was observed in less CpG-rich shelves and open sea regions, which were depleted in hypermethylated DMPs of DNMT3A-dSpCas9 and TET1-dSpCas9 samples. This could be explained by the fact that CpG-density negatively correlates with DNA methylation levels, as reported in multiple studies (53–55). Thus, CpG islands will have low methylation levels, and hence, will be more susceptible to methylation by DNMT3A than to further demethylation by TET1. The reverse was observed in less CpG-rich shelves and open sea regions, which were depleted in hypermethylated DMPs of the dCas9-DNMT3A and dCas9-TET1 samples. Hypermethylation of these regions would be less dynamic, due to their tendency toward relative hypermethylation at baseline (56). Similarly, in terms of regulatory regions, DNMT3A-dSpCas9 induced hypermethylation preferentially targeted promoters (strong or weak), which are often CpG-rich (Supplementary Figure S13A), while the TET1-dSpCas9 induced hypomethylation was enriched at enhancers (strong or weak), repressed regions and transcription regulatory sites (Supplementary Figure S13B). Some hypermethylation effect by DNMT3A-dSpCas9 was also seen, though to a lesser extent, at strong enhancers and insulator regions.

This study provides a proof of concept that externally imposed CpG methylation in promoters of the candidate genes, relevant for protein glycosylation, can change their transcription level and consequently have an effect on the glycan phenotype. The newly developed CRISPR/dCas9-based modular toolbox is therefore very useful for studying gene regulatory networks that regulate IgG glycosylation involved in inflammation, which is essential for our effort within the frame of the H2020 flagship interdisciplinary consortium SYSCID (‘A systems medicine approach to chronic inflammatory disease’) with the goal of understanding molecular mechanism underlying chronic inflammatory disease. In general, such molecular toolbox could have a great potential in therapeutic strategies for disorders that involve epigenetic silencing. Finally, a molecular toolbox with exchangeable effector domains, Cas9 orthologs, selection markers and weak/strong promoters provides an extensible platform suited for resolving open questions in the field of targeted epigenome editing as well as fundamental questions related to the role of epigenetic marks in gene regulation. For instance, the use of xCas9 variants with broad PAM recognition and higher DNA specificity (28,42) could further reduce off-target activity. The use of some other epigenetic ‘writers’ and ‘erasers’ for simultaneous epigenetic editing could reveal causal relationship between directly manipulated individual epigenetic marks other than CpG methylation and gene transcription, thus helping in better understanding of the link between the complex chromatin layer, transcriptional regulation and cell function (21).

## Supplementary Material

gkz709_Supplemental_Files
