# Supplementary material for: Antagonistic and synergistic epigenetic modulation using orthologous CRISPR/dCas9-based modular system
Source: Nucleic Acids Res. 2019 Aug 14;47(18):9637–57. doi: 10.1093/nar/gkz709 (PMC6765142; doi:10.1093/nar/gkz709)
Supplement: gkz709_Supplemental_Files [file gkz709_supplemental_files.zip › SUPP_EpiToolBox_REVISION_submit.pdf]

SUPPLEMENTARY MATERIAL

Supplementary methods

Construction of plasmids for modular assembly

The backbone plasmid (pBackBone-BZ) was derived from pUC19 (Addgene plasmid #50005). First, the undesired BsaI restriction site in the beta-lactamase ORF was removed by site-directed mutagenesis. Next, an 1800 bp fragment containing the beta lactamase expression cassette and the origin of replication was amplified by PCR from pUC19 using primers pUC19-FW and pUC19-RE. The PCR product was joined, using Acc65I and AgeI restriction endonucleases, with a custom-synthesized gene fragment "Gblock-BB" amplified using primers C9seq1 and C9seq2 (Supplementary Table 4) and cut with type IIS restriction endonuclease Esp3I to generate compatible ends. SV40 origin of replication was removed by restriction with NgoMIV and re-ligation of the larger fragment. The final construct contained a DTS region (TF binding region from SV40) for efficient import of the plasmid into the nucleus (Dean, 1997) and a section with outward-facing BsaI restriction sites creating "B" and "Z" type ends for golden-gate assembly (Supplementary Figure S3). This section also contained an expression cassette for LacZα with lac promoter and the L3S1P13 synthetic terminator (Chen et al., 2013) for blue-white selection of undigested or re-ligated backbone vector during golden-gate assembly.

Modules were cloned into the pUK21 vector (Addgene plasmid #49788) or our derived vector pUK21gg (optimized for Golden Gate cloning using type IIS restriction endonucleases). Each module was amplified by PCR using primers with appropriate restriction sites compatible with pUK21 MCS (Supplementary Table 4). The primers also contained inward-facing BsaI recognition sequences that upon digestion leave appropriate four bp ends used for subsequent Golden Gate assembly (type dependent on module position; Supplementary Figure S3). All undesired BpiI, BsaI and Esp3I restriction sites were removed by site-directed mutagenesis (Supplementary Table 4).

Module for VPR domain was amplified from the plasmid SP-dCas9-VPR (Addgene plasmid #63798); catalytically inactive dSaCas9 was amplified from pX603-AAV-CMV::NLS-dSaCas9(D10A,N580A)-NLS-3xHA-bGHpA (Addgene plasmid #61594); EFS promoter was amplified from AAV:ITR-U6-sgRNA(backbone)-pEFS-Rluc-2A-Cre-WPRE-hGHpA-ITR (Addgene

plasmid #60226); fluorescence marker mRuby3 was amplified from the plasmid pKanCMV-mClover3-mRuby3 (Addgene plasmid #74252); puromycin resistance gene was amplified from lentiCRISPR v2 (Addgene plasmid #52961); TET1 domain was amplified from pJFA344C7 (Addgene plasmid #49236). Catalytically inactive TET1 (as negative control) was created by site-directed mutagenesis by introducing H1671Y and D1673A mutations in TET1 active site (see annotations in pFD-TET1_NCTRL plasmid sequence). All other modules were amplified from plasmids published in our earlier work (Vojta et al., 2016). Coding sequences for modules containing fluorescent and antibiotic resistance markers (PuroR, mRuby3, mClover3) were cloned using a streamlined strategy, with the NcoI restriction site around the start codon and the KasI site encoding the last two amino acids (Gly-Ala) of the coding sequence. All NcoI and KasI restriction sites were removed from the coding sequence of markers by introducing silent mutations by site-directed mutagenesis (Supplementary Table 4).

Destination plasmid for selection modules (fluorescent and antibiotic resistance markers) was pUK21_FP_entry that contains custom synthesized gene fragment pFP-Entry cloned between PstI and XhoI restriction sites in pUK21 vector lacking KasI restriction site. KasI restriction site was removed by inserting short oligonucleotide (pUK21_KasI-BamHI) between KasI and BamHI restriction sites in pUK21 vector. Fragment pFP-Entry contains eukaryotic promoter EFS, as well as prokaryotic Lac promoter for validation of fluorescence signal in animal and bacteria cells respectively. In addition, two BpiI restriction sites in fragment can later release fluorescence module under the Lac promoter for its insertion into gRNA modules and use in red-white selection. PCR amplified fluorescence modules mRuby3 and mClover3 were then cloned using NcoI and KasI strategy into pUK21_FP_entry, resulting with plasmids pUK21_FP_mClover3 and pUK21_FP_mRuby3, that can be used for the construction of single or dual marker module (described later).

For red-white selection, coding sequence for mRuby3 under the Lac promoter was cut from the finished mRuby3 module with BpiI restriction enzyme (described above) and inserted into the pSg-Sp module (gRNA module for SpCas9) within two BpiI restriction sites used for insertion of gRNA variable part. Module pSg-Sa (gRNA module for SaCas9) was amplified from the pX601-AAV-CMV::NLS-SaCas9-NLS-3xHA-bGHpA;U6::BsaI-sgRNA (Addgene plasmid #61591); original Esp3I sites for gRNA cloning were replaced with BpiI sites and mRuby3 cassette for red-white selection was added analogously to pSg-Sp.

Modules dSpCas9 and dSaCas9, initially made for C-terminal effector domain fusion, were repurposed for the better-performing N-terminal fusion. Specific Golden Gate ends “I” and “II” were changed into “II” and “III”. To do that, specific annealed oligonucleotide (N-C9), defining the new ends, was cloned between HindIII and XhoI restriction sites in the pUK21gg vector. Modules dSpCas9 and dSaCas9 were then cut out using BsaI and cloned into two BpiI restriction sites in pUK21gg_N-C9.

Functional modules for N-terminal fusion with dCas9 module were made analogously. Initially, ends of types “II” and “III” were changed into types “I” and “II” by cloning DNMT3A, TET1 and VPR modules into two BpiI restriction sites in pUK21gg_N-FD.

In C-terminal fusions with dCas9, the nucleoplasmin NLS might be covered by effector domain (DNMT3A). Thus, to test the effect of additional nucleoplasmin NLS on C-terminus of DNMT3A effector domain, specific annealed oligonucleotide FD_c-NLS-NP was cloned between KasI and XhoI restriction sites in the pUK21gg vector. The oligonucleotide contains two Esp3I restriction sites for DNMT3A cloning along with nucleoplasmin NLS downstream from cloning site. DNMT3A effector domain was then cut out from finished module vector, initially made for C-terminal fusion with dCas9, with BsaI restriction enzyme and cloned into pFD_c-NLS-NP vector.

Construction of the multi-guide system

First, the "individual" modules for golden-gate assembly of gRNAs at positions 1-6 in the multi-guide system were created as follows. Undesired NgoMIV and XhoI restriction sites were removed from the plasmid pFUS_A (Addgene plasmid #31028) and a new NgoMIV restriction site was introduced downstream from the multiple cloning site by site-directed mutagenesis (Supplementary Table 4). Multiple cloning site from the plasmid pUK21 (Addgene plasmid #49788) was cut out with SapI and NgoMIV and inserted into altered pFUS_A, yielding the plasmid pUS21gg. Six different pairs of annealed oligonucleotides (sgM1-6) were then cloned into the KasI and XhoI restriction sites, each carrying two Esp3I restriction sites that create different four nt 5' protruding ends labelled with roman numerals I to VII, corresponding to the ends B-Z in the system for assembly of fusion constructs. An adapter (XbaI_B_C9seq1_A_NcoI) was oligo-annealed and inserted between XbaI and NcoI sites, facilitating BsaI cloning of the finished gRNA cassette (including mRuby3 expression for red-white selection) from the modules pSg-Sa and pSg-Sp, previously created

for single gRNA cloning in the system for dCas9 fusions (described in previous section). Two sets of six different plasmids for cloning of individual gRNA molecules for SaCas9 and SpCas9 were made (pSgMxA represent plasmids for SaCas9 gRNA molecules while pSgMxG represent plasmids for SpCas9 gRNA molecules, x = (1, 2, 3, 4, 5, 6)), that enable assembly (using Esp3I type IIS enzyme) of up to six different gRNA modules, each carrying its gRNA expression cassette.

Next, "multi-guide" modules were created, which replace the gRNA module in the assembly of core fusion constructs and allow cloning of one to six "individual" gRNA modules of the multi-guide system. Kanamycin resistance gene and origin of replication were amplified from pUK21 (Addgene plasmid #49788) with primers that enabled ligation with the multiple cloning site cut out from the same plasmid with NgoMIV and SapI, which resulted in the removal of two undesired Esp3I restriction sites. The third Esp3I restriction site (located within kanamycin resistance gene) was removed by site-directed mutagenesis (Supplementary Table 4), yielding the plasmid pUK21gg. A pair of annealed oligonucleotides (SgMult) containing one restriction site for Esp3I that defines the cohesive end "I" needed for the assembly of the first gRNA module and BsaI restriction site that defines the sticky end named “B” needed for the assembly of functional construct for epigenetic modulation was cloned within KasI and NcoI restriction sites. Six different pairs of annealed oligonucleotides (SgMx1-6), each containing Esp3I restriction site defining the other sticky end named “2-7” that determines the capacity of accepting up to six different gRNA modules assembled and BsaI restriction site that defines the sticky end named “A” are then cloned within NcoI and XhoI sites. In the final step, mRuby3 fluorescence marker under the Lac promoter was cut out from the plasmid containing mRuby3 module (described in previous section) with BpiI and cloned between two BpiI restriction sites that were introduced with the cloning of first pair of annealed oligonucleotides (SgMult). The mRuby3 marker was then used for red-white selection of correct gRNA modules following assembly with Esp3I restriction enzyme. The final products of this step were plasmids (pSg-x1 to pSg-x6) that have capacity to receive up to six gRNA modules with Esp3I-mediated assembly.

Dual marker modules

Plasmids for the dual marker system (Figure 3C in the main text) at the first position with ends "III" to "X" were generated from the corresponding modules with ends of type "III" and

"IV" by amplifying the T2A region of the plasmid pUK21_FP_T2A (not deposited) using primers T2A_X-FW and T2A_X-RE (Supplementary Table 4), which added the non-complementary extensions needed to convert the end "IV" into "X". The PCR product was then cloned between HindIII and XhoI restriction sites into the plasmid pUK21_noKasI (lacking KasI restriction site; described in section: Construction of plasmids for modular assembly) and the intermediary plasmid was named pM2-FP_T2A-X.

For the second position, an "empty" plasmid pM2-FP_X-P2A was generated by amplifying custom-synthesized DNA fragment Gblock-BB (also used for backbone plasmid construction; described in section: Construction of plasmids for modular assembly) using primers C9seq3 and C9seq4 and cloning the PCR product into the pUK21_FP_T2A vector between the NcoI and HindIII restriction sites.

Marker coding sequences for antibiotic resistance or fluorescent proteins (from previously constructed modules) were subsequently inserted into pM2-FP_T2A-X and pM2-FP_X-P2A using the streamlined cloning strategy: coding sequences were flanked by NcoI and KasI restriction sites, which were used to seamlessly shift the existing coding sequences into new empty module vectors.

Oligo cloning of variable parts into gRNA modules

Variable parts of gRNA were cloned into appropriate modules essentially as described in (Cong et al., 2013). Briefly, oligonucleotides designed to form dsDNA with overhangs compatible with gRNA modules (Supplementary Figure S4) were custom-synthesized. They were phosphorylated at the 5' end and annealed in a reaction containing 100 pmol of each oligonucleotide, 1× T4 ligation buffer (TaKaRa) and 5 U of T4 polynucleotide kinase (NEB). Phosphorylation was done at 37°C for 30 min, followed by denaturation at 95°C for 5 min. Oligonucleotides were annealed in a thermocycler by gradual decrease of temperature by 5°C per minute, from 95°C down to 25°C. Phosphorylated and annealed oligonucleotides were then cloned into module plasmids in a single reaction containing 1× Buffer G (Thermo Fisher Scientific), 0.5 mM DTT (Thermo Fisher Scientific) 0.5 mM ATP, 350 U of T4 DNA Ligase and 10 U of type IIS restriction enzyme BpiI (Thermo Fisher Scientific). Reaction conditions consisted of six cycles at 37°C for 5 min and 23°C for 5 min. Exonuclease V treatment was performed by directly adding 10 U of enzyme into the reaction mix along with additional 0.5 mM ATP and incubating for 30 min at 37°C to remove any remaining linear DNA. After

bacterial transformation, white colonies were selected for further verification (red colonies represented uncut or empty backbone vector).

Testing of different linkers between dSaCas9 and effector domains

The length between effector domains linked to the C-terminus of *Staphylococcus aureus* Cas9 was varied to find the optimal spacing that would enable the fusion construct to access the target DNA. First, dSaCas9 along with C-terminal nucleoplasmin NLS was amplified from pX603-AAV-CMV:NLS-dSaCas9(D10A,N580A)-NLS-3xHA-bGHpA (Addgene plasmid #61594). Alternatively, dSaCas9 with three tandem HA epitope tags located downstream from the nucleoplasmin NLS, which extend the spacing between dSaCas9 and effector domain, was amplified from the same plasmid (Supplementary Table 4). Both versions of dSaCas9 were then cloned into pUK21 (Addgene plasmid #49788) between XbaI and XhoI restriction sites in a version of modules for fusions of effector domains at the C-terminus of dSaCas9.

To test trip-zip linker (TZ2) (Cochran, Skelton, & Starovasnik, 2001), it was added to the dSaCas9 module by oligo annealing of primers LNK_tripzip2_G4Sx2-S and LNK_tripzip2_G4Sx2-A and cloning into a dSaCas9 module (for assembly of effector domains fused to the C-terminus of dCas9) using BamHI and XhoI restriction enzymes, thus generating SaCas9_TZ2_2xG4S module (not deposited).

Confirming dual transfection using fluorescence markers

We monitored transfection of HEK293 and BG1 cells with plasmids for expression of fusion proteins by observing red mRuby3 and yellow-green mClover3 (Bajar et al., 2016) fluorescence of the fused fluorescent proteins, translated in the same reading frame as the dCas9 fusion construct and linked via self-cleaving 2A peptides. One day after transfection, images were acquired under the same conditions using an Olympus IX73 microscope. Cells positive for mClover3 and mRuby3 fluorescence were counted using Object Count tool in Olympus cellSens Standard software. Fluorescence was imaged using Olympus filter sets U-FRFP (mRuby3) and U-FYFP (mClover3), which ensured good separation of fluorescent signals.

Plasmids for whole-genome methylation analysis

The SV40 promoter and terminator were amplified from the plasmid pLVET-tTR-KRAB (Addgene Plasmid #11644) and cloned into the pUK21 (Addgene plasmid #49788) multiple cloning site. Both pairs of primers contained BsaI restriction sites that can release modules from pUK21 with specific four nt overhangs for joining with the puromycin resistance module. For that purpose, we changed the puromycin resistance module type "III" overhangs into type "I" to enable its BsaI assembly with SV40 promoter, while other overhang of type "IV" is necessary for assembly with terminator. A pair of annealed oligonucleotides (M14) that contained two BsaI restriction sites defining the overhangs type "I" and "IV" was cloned between KasI and XhoI restriction sites in pUK21. Puromycin resistance module was then cut out from the finished plasmid (as described in Construction of plasmids for modular assembly) with NcoI and KasI and cloned into the pUK21_M14.

To create a backbone with the secondary puromycin expression cassette, we inserted a pair of annealed oligonucleotides (BB_2nd_Cassette) into the plasmid pBackBone-BZ upstream of the DTS nuclear import sequence between AatII and SacI restriction sites. The insert contained two Esp3I sites needed for separate assembly of SV40 promoter, puromycin resistance gene and SV40 terminator. For that purpose, modules were cut out with BsaI, while the backbone was cut with Esp3I, which left compatible overhangs for assembly of the secondary expression cassette. The prepared backbone and module fragments were gel-purified and ligated.

Four different gRNA molecules for *IL6ST* and five for *MGAT3* locus (Supplementary Table 3) were cloned using the multi-guide system and the final BsaI assembly was done with both the standard backbone (pBackBone-BZ) and the backbone with she secondary puromycin expression cassette (described above).

Supplementary material describing Illumina 850k analysis

Pre-processing and quality control

Preliminary quality control indicated that all samples were of good quality (Supplementary Figure S10A), and that there were no great differences in red and blue signal intensities between the samples (Supplementary Figure S10B). After normalization, multidimensional scaling was used to visualize the similarity of individual cases within the dataset. Here, it was observed that there was a horizontal effect segregating biological replicates 1 and 2, as well

as a vertical effect segregating the TET1-dSpCas9 and TET1-dSpCas9 secondary cassette samples from the samples in the Mock, DNMT3A-dSpCas9 and DNMT3A-dSpCas9 secondary cassette groups. The variation between the biological replicates could be attributed to the fact that each set of technical (and hence biological) replicates were run on separate chips (Sentrix_IDs). Indeed, PCA analyses indicated that Sentrix_ID, Biological Replicate and Technical Replicate contributed significantly to principal component 2 (Supplementary Table 7). These effects were abrogated after SVA correction, as evidenced by the PCA analyses (Supplementary Table 8) and by the multidimensional clustering of samples by experimental condition rather than by biological/technical replication (Supplementary Figure S10 C and D, that is, the horizontal effect segregating biological replicates disappeared after SVA

Methylome-wide variations by DNMT3A-dSpCas9 and TET1-dSpCas9

Unsupervised multidimensional scaling of methylome-wide variations identified three distinct clusters, clearly distinguishing each of DNMT3A-dSpCas9, TET1-dSpCas9 and mock-transfected cells, respectively (Supplementary Figure S10D). Moreover, the constructs with DNMT3A-dSpCas9 expressed under a strong (primary cassette, marker expressed fused via 2A self-cleaving peptide) or a weak promoter (secondary cassette, marker expressed separately under a strong promoter) were clearly distinguishable within their relevant cluster, and this was also seen, though to a lesser extent, with the primary and secondary constructs of TET1-dSpCas9 (Supplementary Figure S10D). Biological and technical replicates clustered together, showing low experimental variation (Supplementary Figure S10D). Overall, the largest observed methylome variations were induced by each of DNMT3A-dSpCas9 and TET1-dSpCas9 relative to mock, with lesser but evident effects seen between the constructs expressed from strong vs. weak promoter. Those effects were stronger than the basal experimental variations between technical or biological replicates.

Supplementary figures

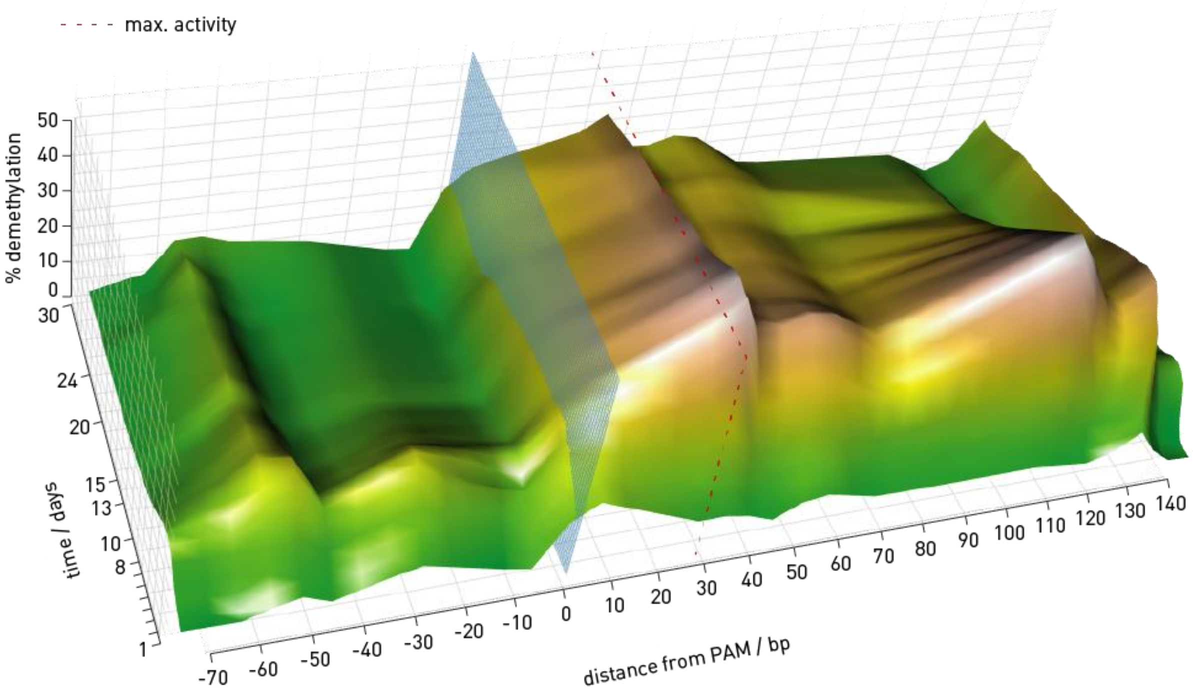

Supplementary Figure S1. Time course of dSpCas9-TET1 demethylation activity. Data from several experiments – loci *MGAT3* (2 assays: A1 and A2, 9 + 5 CpG sites; 8 gRNAs) and *LAMB1* (one assay, 6 CpG sites; 2 gRNAs) – provide a comprehensive picture of TET1 activity when fused to C-terminus of dSpCas9. The *x*-axis shows distance from gRNA binding site (oriented relative to PAM sequence, represented by the blue plane at position zero), while the *y*-axis shows time in days; the *z*-axis ("height") corresponds to CpG demethylation activity at a given position at selected time points. The activity peak is clearly distinguishable at about 30 bp from the PAM sequence, with minor other satellite peaks some distance apart, which might represent contact with adjacent nucleosomes. Thirty days following transfection, the main peak remained fairly stable, while the satellite peaks reverted almost to their original methylation level.

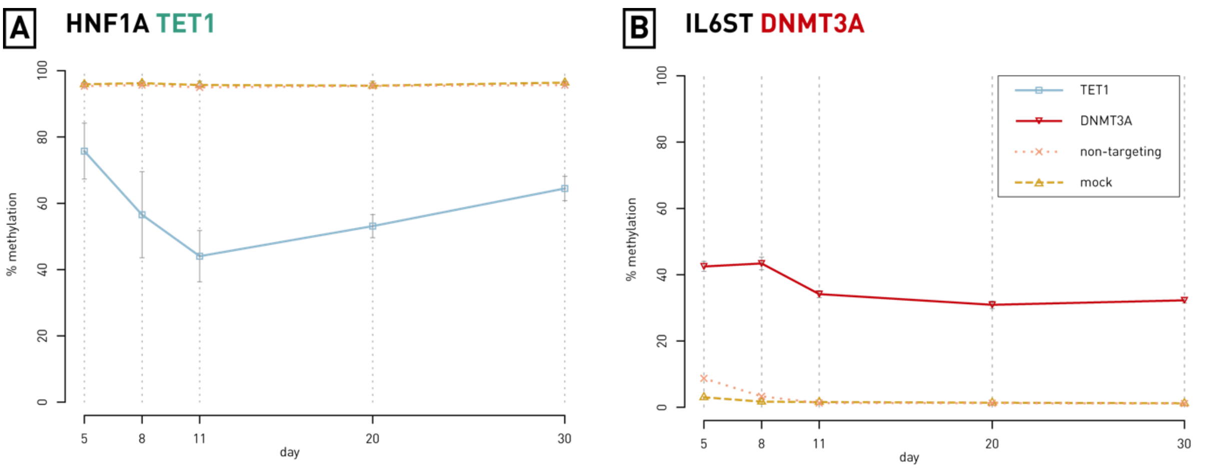

Supplementary Figure S2. Time course of targeted methylation and demethylation. Effect of demethylation of *HNF1A* by TET1-dSaCas9 (A) and methylation of *IL6ST* by DNMT3A-dSpCas9 (B) was followed during 30 days; most of the change in methylation persisted throughout the whole period.

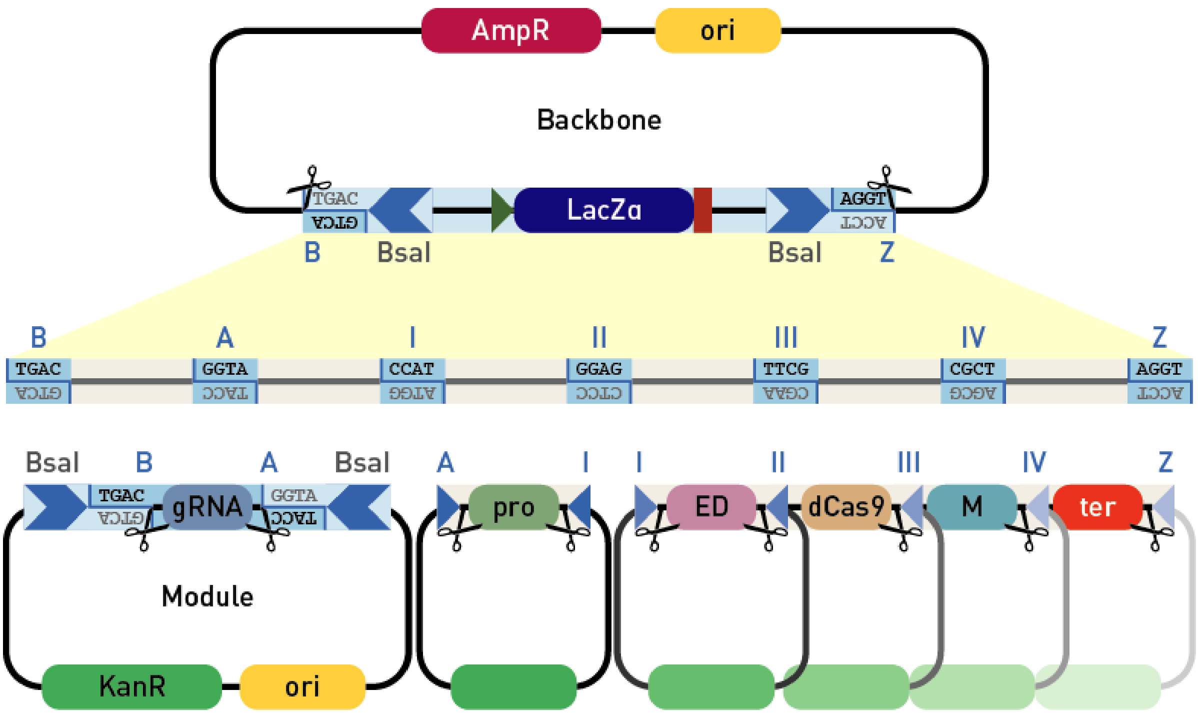

Supplementary Figure S3 Golden Gate assembly of individual modules into a cassette for eukaryotic expression. The components are joined via non-palindromic four nt cohesive ends generated by type IIS restriction enzymes (Golden Gate cloning), which enables efficient assembly of up to eight DNA fragments. The backbone plasmid contains a lacZα cassette for blue-white selection of bacterial clones with uncut/re-ligated backbone, while bacteria taking up only module plasmids are eliminated by counter-selection with ampicillin. White colonies contain the correctly assembled eukaryotic expression cassette for the selected dCas9 fusion construct. If the gRNA module has no pre-cloned variable part or accepts multiple modules for a second round of multi-guide assembly, the positive colonies are red and the gRNA (or multi-guide) cloning is facilitated by red-white selection.

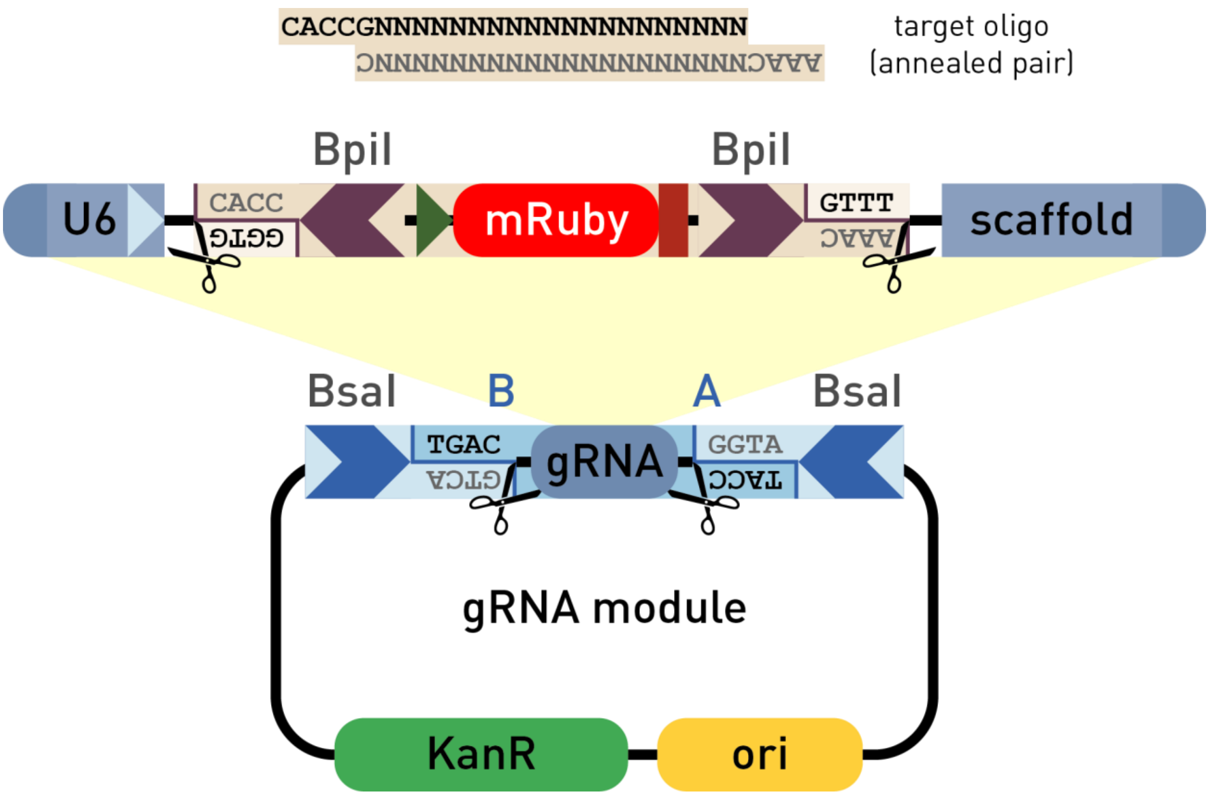

Supplementary Figure S4. The 20 bp gRNA variable region is cloned into the module vector by oligo annealing. Every individual gRNA module contains a U6 promoter, gRNA scaffold for either SaCas9 or SpCas9 and a fragment to be excised by the type IIS endonuclease BpiI at the 5' end of the gRNA scaffold, which prepares the vector for accepting the 20 bp variable gRNA fragment (inserted by oligo cloning) conferring specificity for a particular genomic region. The excised part encodes a bacterial cassette for expression of the mRuby3 fluorescent protein, which gives colonies a red color thus facilitating red-white selection. Alternatively, the gRNA module can be substituted for a multi-guide module accepting one to six gRNA modules for a second round of assembly.

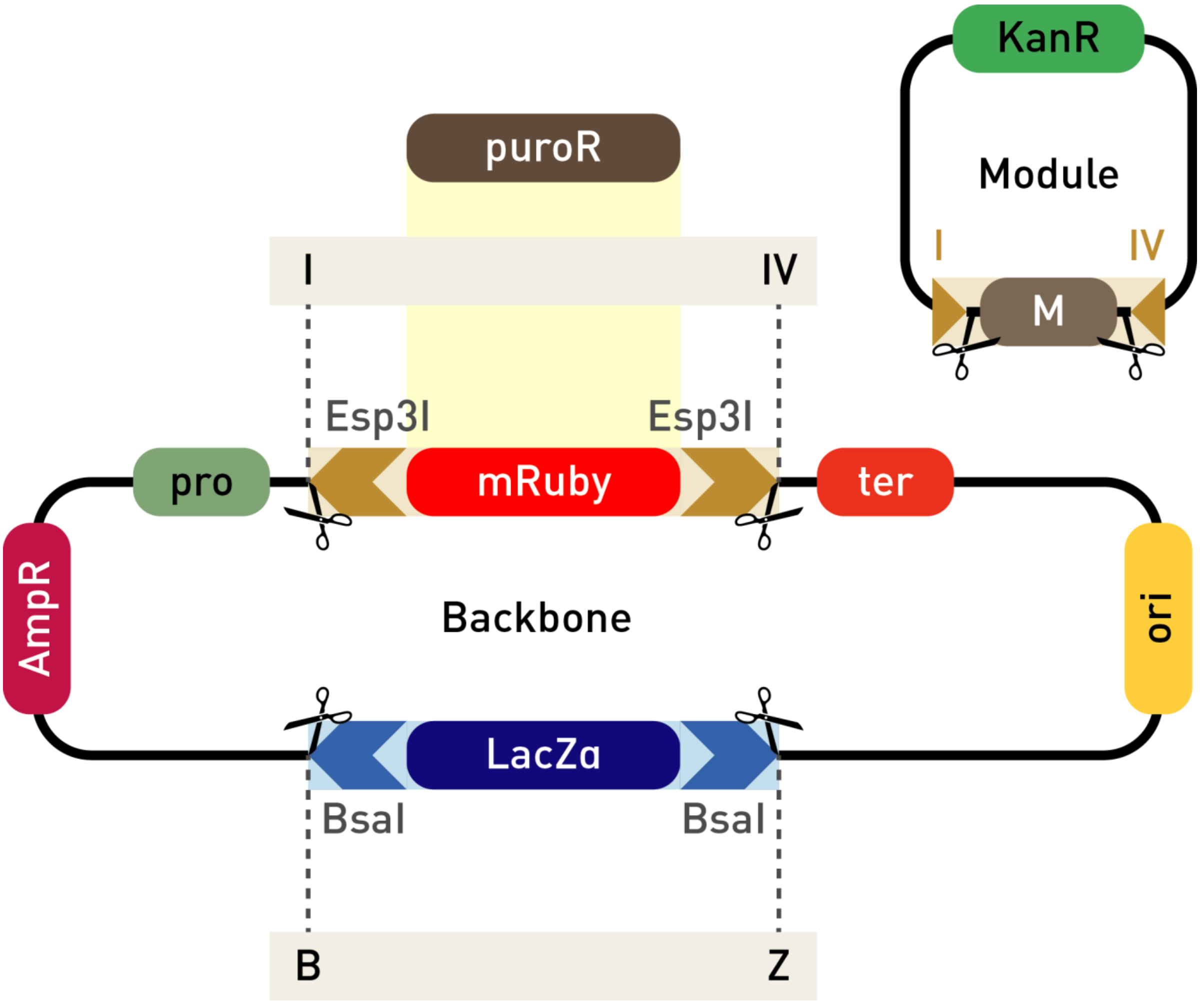

Supplementary Figure S5. Secondary cassette for expression of selection markers separately from the dCas9/ED fusion. Alternative backbone accepts an additional module for the marker protein via Esp3I Golden Gate cloning. Afterwards, the marker is expressed under a strong (SV40) promoter independently of the main fusion construct assembled using the standard protocol. This enables strong selection (especially by puromycin) while giving the flexibility to choose a weaker promoter for the main dCas9 fusion cassette, thereby enabling fine tuning needed for controlling the off-target effect.

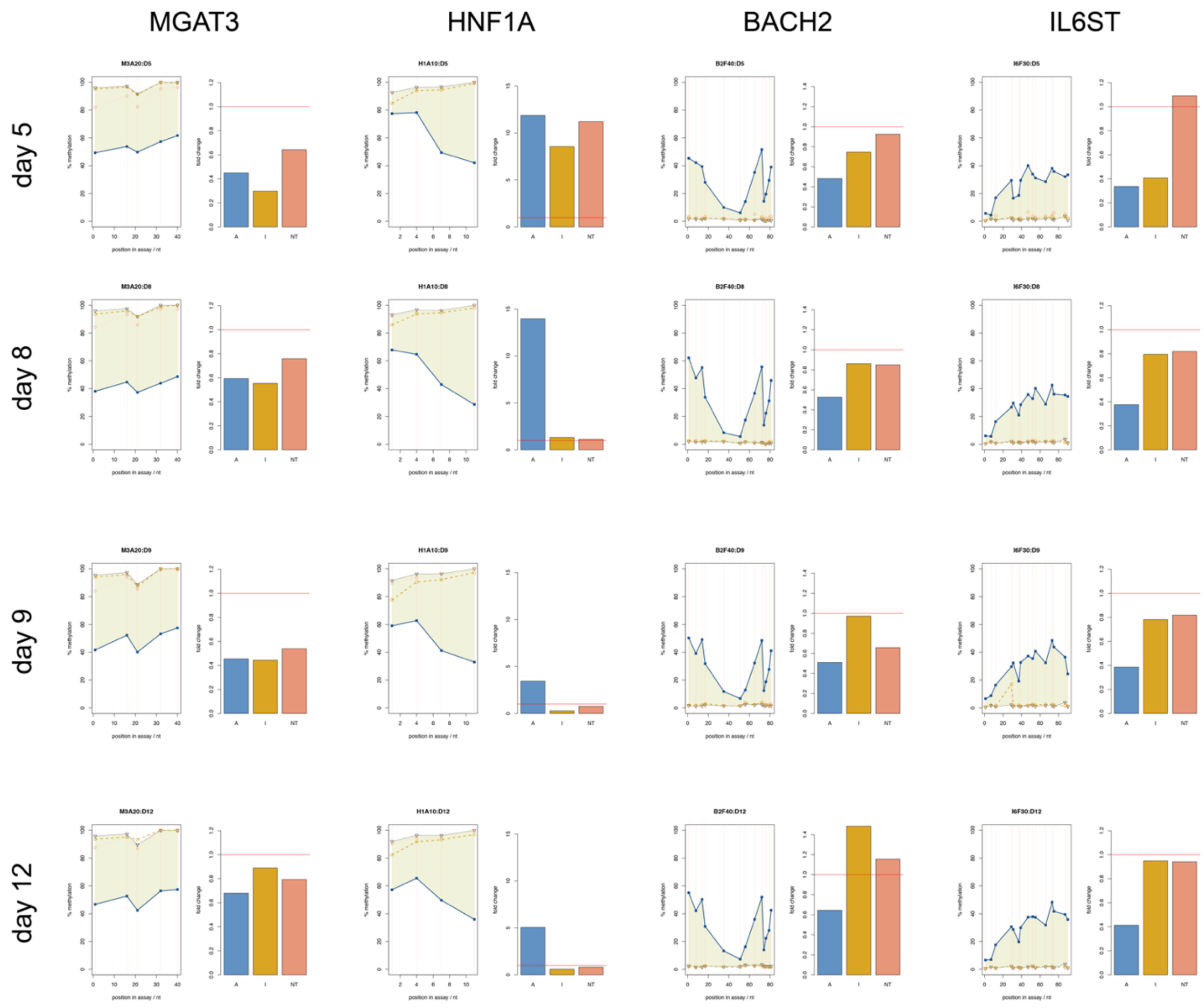

Supplementary Figure S6. Time course of simultaneous methylation and demethylation of two loci using dCas9-based tools with antagonistic activities in HEK293 cells.

Time course experiment shows how altered gene expression reflects changes in CpG methylation levels. We confirmed temporal activity profiles of both DNMT3A and TET1 fusions to dCas9 (both dSpCas9 and dSaCas9) to be similar to the profiles obtained for dSpCas9-DNMT3A alone (Vojta et al., 2016): a rapid rise of methylation activity after transfection up to day 8, with slowly diminishing effect afterwards. The expression profile closely followed CpG methylation profile. Thus, with this experiment we verified that the peak of methylation and consequent change in expression falls at the day 8 after transfection. A – dCas9 fusions with active catalytic domain; I – dCas9 fusions with inactive catalytic domain; NT – active dCas9 fusions with non-targeting gRNA.

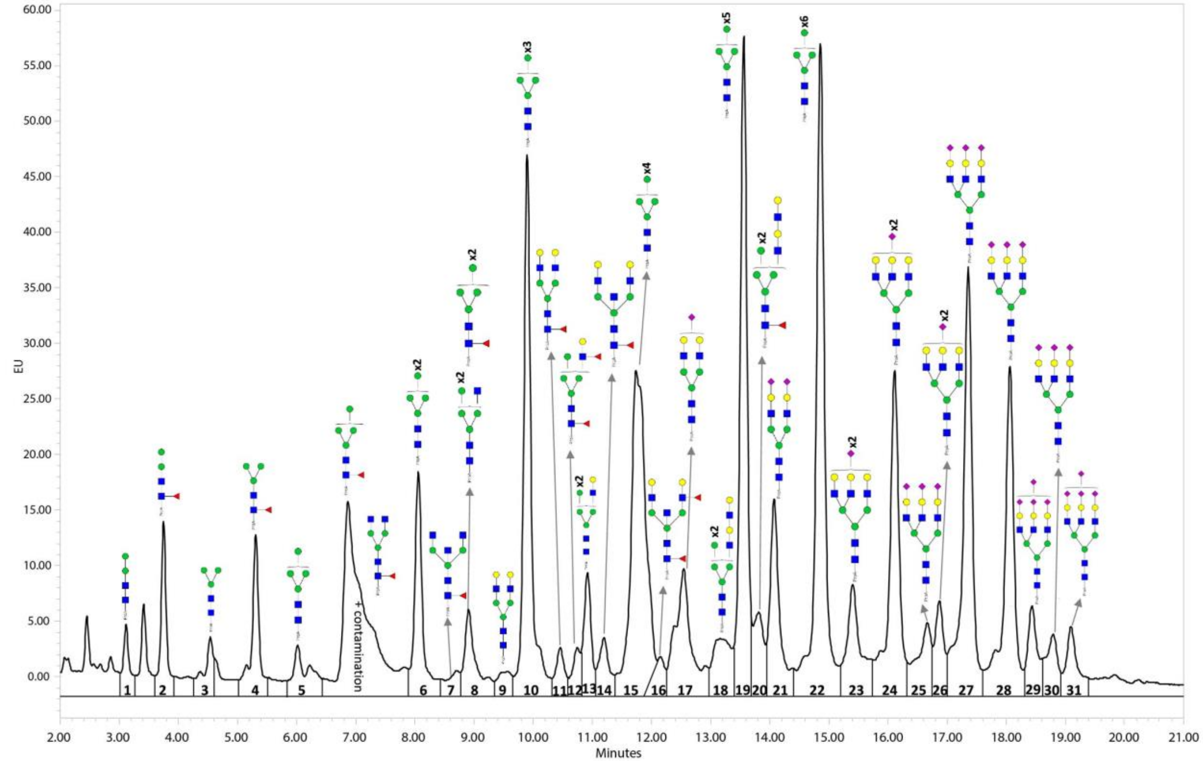

Supplementary Figure S7. Annotated glycan peaks. Each glycan peak in the chromatogram has been assigned corresponding structures based on mass spectrometry analysis. See Supplementary Table 6 for glycan peak annotations.

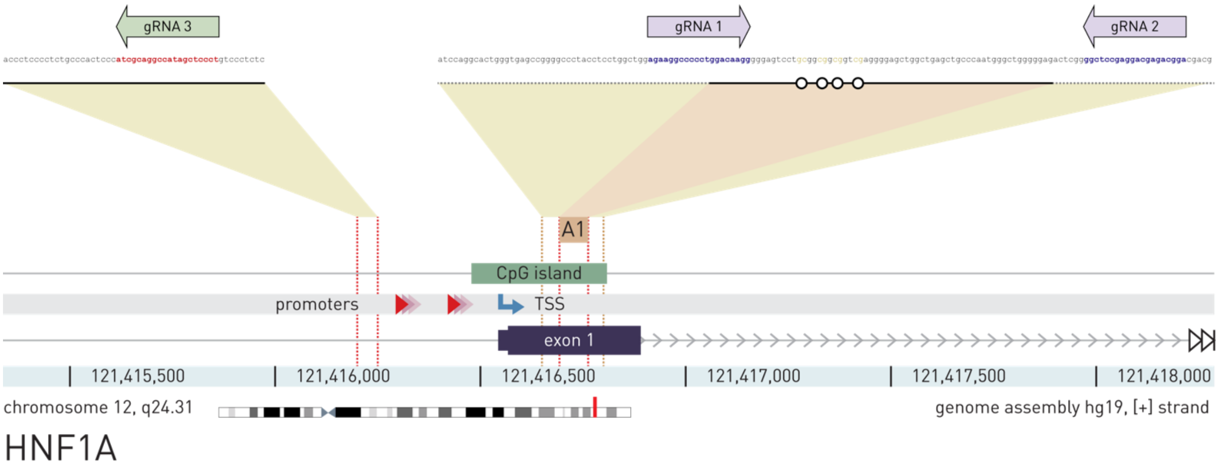

Supplementary Figure S8. Position of gRNAs used for synergistic activation of *HNF1A* by TET1-dSaCas9 and VPR-dSpCas9. The lavender colored arrows show positions of gRNA1 and gRNA2 for guidance of TET1-dSpCas9, while the green arrow shows the gRNA3 for guidance of VPR-dSpCas9. Arrows point in the direction of the PAM sequence. A1 is the pyrosequencing assay for *HNF1A*.

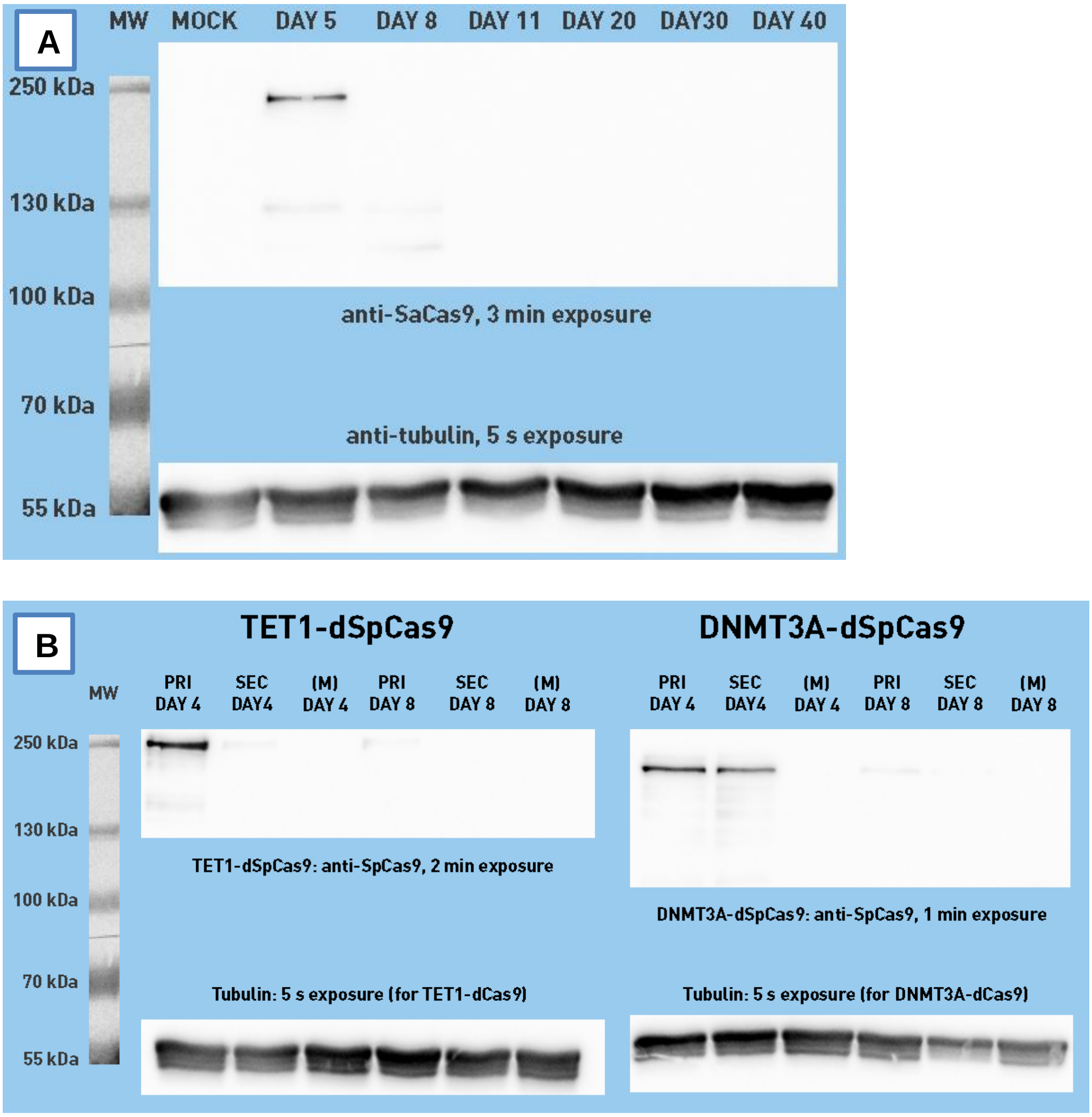

Supplementary Figure S9. Original western blots for protein presence (dCas9) in the time course (A) and "strong vs. weak promoter" (CBh vs. EFS) (B) experiments. The presented pictures were used for quantitation of protein presence; each lane was normalized to tubulin in order to control for variation in gel loading. (A) The time course shows that no protein was detectable by day 11 after transfection. (B) Stronger expression of the "primary cassette" construct (dCas9 fusions under CBh promoter, marker in frame joined by 2A peptides) compared to the "secondary cassette" construct (dCas9 fusions under the weak EFS promoter, marker under the stronger SV40 promoter) is apparent at both timepoints (days 4 and 8 after transfection). After quantitation, the ratio of expression from primary to secondary cassette is: for TET1-dSpCas9, 32.6 on day 4 and 6.8 on day 8; for DNMT3A-dSpCas9, 1.4 on day 4 and 1.2 on day 8.

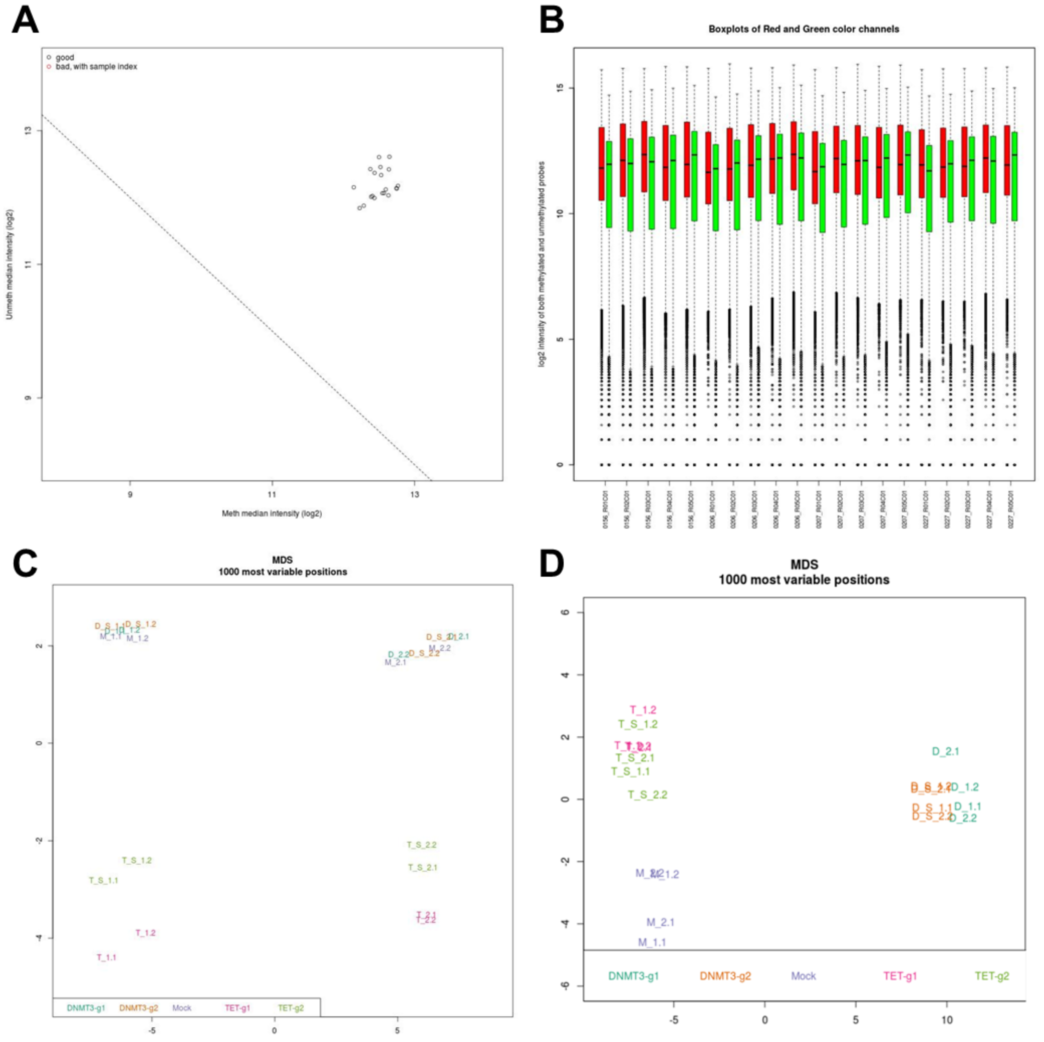

Supplementary Figure S10. Pre-processing and quality control plots. (A) QC plot indicating the log median intensity in both methylated and unmethylated channels. High median intensities and clustering of samples is an indication of good quality. (B) Boxplot showing the spread of log2 intensity in both methylated and unmethylated channels. (C and D) Multidimensional scaling (MDS) plots (C) before and (D) after SVA correction.

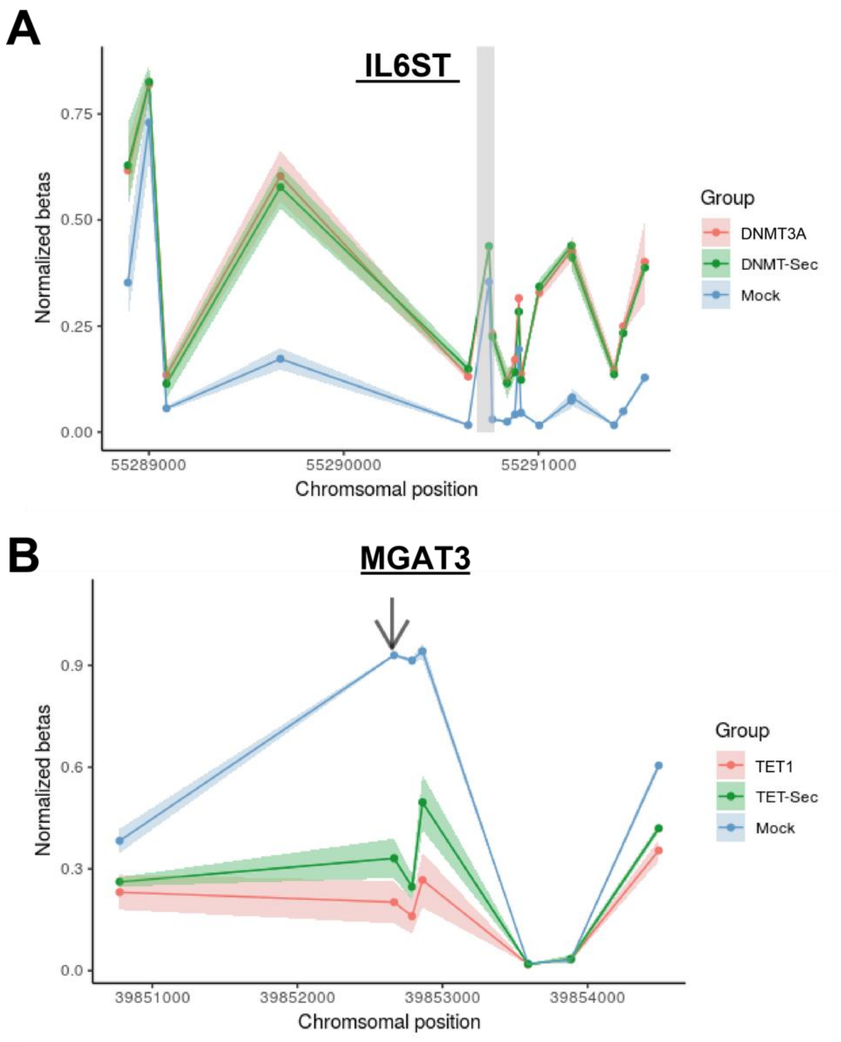

Supplementary Figure S11. Targeted modification to DNA methylation patterns can be induced by DNMT3A-dSpCas9 and TET1-dSpCas9 constructs. (A) Comparison of DNA methylation patterns induced by DNMT3A-dSpCas9 primary and secondary cassettes on probes 2500 bp up- and downstream of the targeted region (shaded in grey) of *IL6ST*. (B) Comparison of DNA methylation patterns induced by TET1-dSpCas9 primary and secondary cassettes 2500 bp up- and downstream of the targeted region of *MGAT3*. The probe cg21461856 is the only probe lying within this region (indicated with an arrow). Normalized betas were used to plot the above graphs, as opposed to SVA-corrected betas in Supplementary Figure S10.

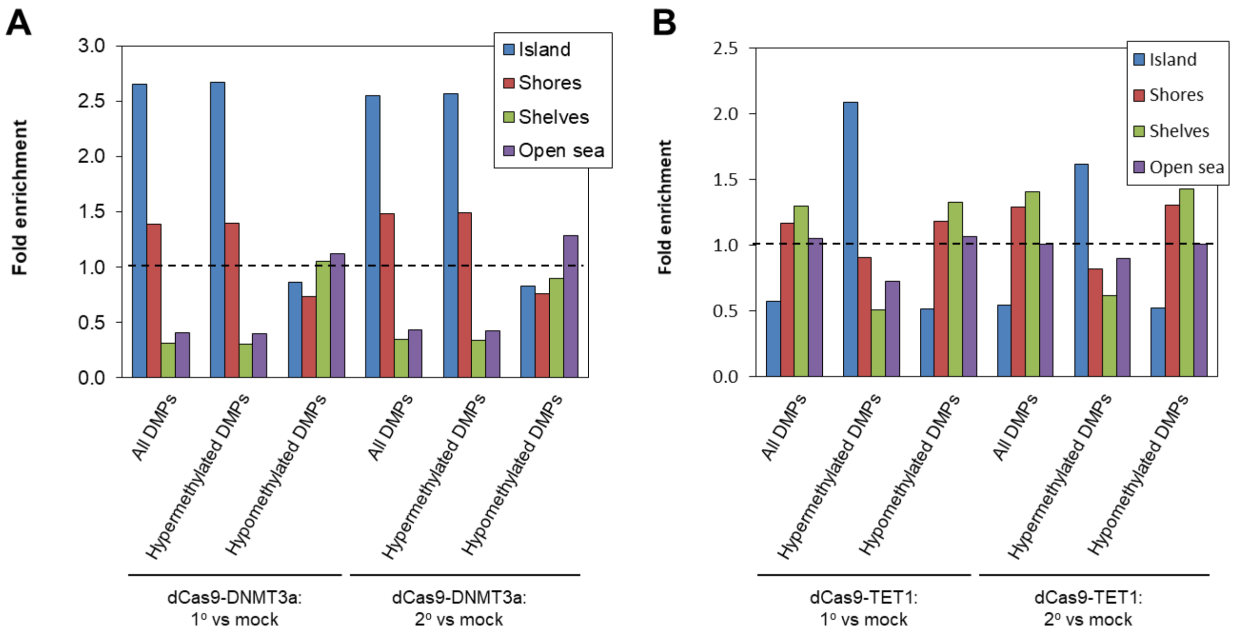

Supplementary Figure S12. Distribution of DMPs by relationship to CpG islands for (A) DNMT3A-dSpCas9 primary and secondary cassettes and (B) TET1-dSpCas9 primary and secondary cassettes. Fold enrichment was calculated by dividing the fraction of DMPs in each annotation category against the fraction of all filtered probes mapped to the same category, correcting for differences in representation of each annotation category on the Infinium MethylationEPIC array.

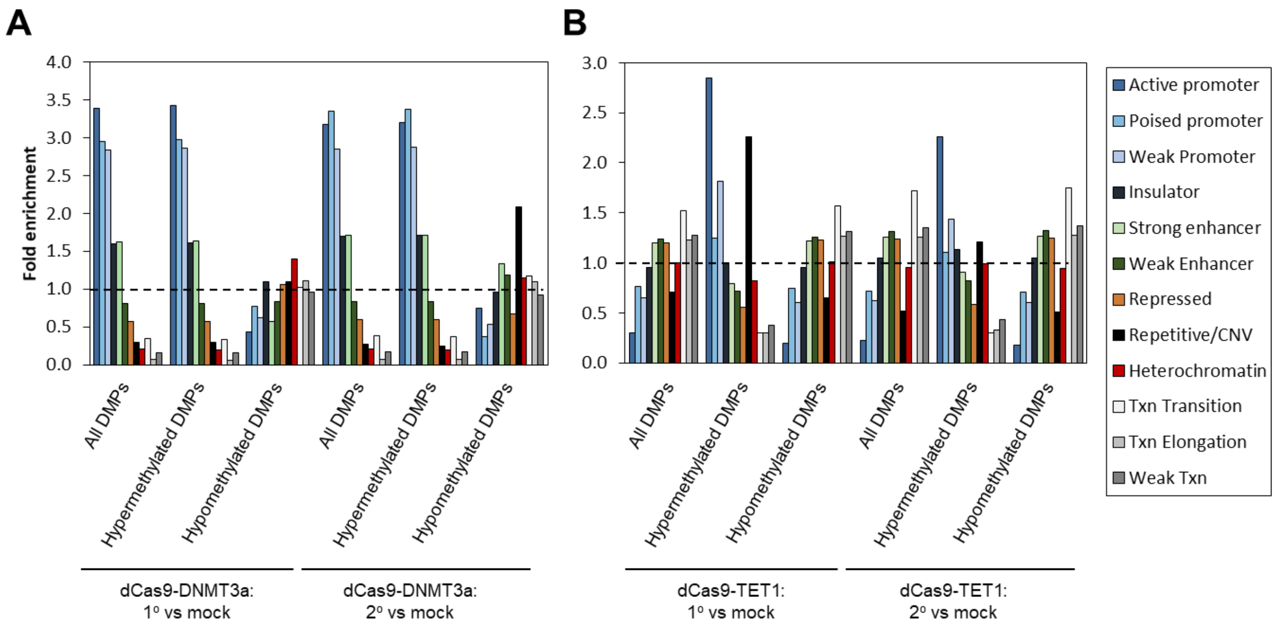

Supplementary Figure S13. Distribution of DMPs across various regulatory regions for (A) DNMT3A-dSpCas9 primary and secondary cassettes and (B) TET1-dSpCas9 primary and secondary cassettes. Fold enrichment was calculated by dividing the fraction of DMPs in each annotation category against the fraction of all filtered probes mapped to the same category, correcting for differences in representation of each annotation category on the Infinium MethylationEPIC array.

Supplementary Table 1. Sequences of pyrosequencing assays used for methylation analysis

Assay name	Assay sequence 5'→3' (analyzed CpG sites are underlined)
**MGAT3-A1**	CGCATCCCTGCACCTTCGACGATGGCGGCGCAGAGATGTCTGCTGCGTACCCACAATGCCTTGTGCCTCGCACCGCGGGA
**MGAT3-A2**	CCTAGAGCAAGGCCACGAGGAGCCAGGGCACGACACGGTGGGCCCTCGGAGAACCGCTG
**MGAT3 A-TIS**	CGCGCCGCGCTCGGCCTCGCCTCCGCGCCCCCCGCGCGCCCGCCGCGGTCCCTCCCCCGCGCCCGTCCGCTCGCCGGGCCCCCGCCGCCGCCCCGGGGTGCAGCCGAGCGGCCGCGCCGGGTCCCCGGGACGGGGTG
**BACH2-A1**	GCGGGTGACGTCAGCGCCGAATGTCAACAATGTAGCGATTGAGAGTGTGGGCGTTCCGGGGAGAGCGCAAGCCGCGCGGCGCG
**BACH2-A2**	AACGGGAAGGGGGTGGGAGCCGAGCCGGCGGCCGCGCCGCGCCGAATGTGTGCTCTCCCTCCCGGCTGTTTACGGCGGCCGAGGCG
**HNF1A-A1**	TGCGGCGGCGGTCGAGG
**IL6ST-A1**	CGGAGCCGGGGCGAGCAGCCAAAAGGCCCGCGGAGTCGCGCTGGGCCGCCCCGGCGCAGCTGAACCGGGGGCCGCGCCTGCCAGGCCGACGGG

Supplementary Table 2. Sequences of PCR primers and pyrosequencing primers

Primer name	Sequence (5' → 3')	Use
**MGAT3-A1-Fw**	GTTGGGATATAGAATAGGTAG	PCR amplification of MGAT3-A1 fragment, forward primer was used for pyrosequencing
**MGAT3-A1-Rev**	[Btn]ACCATTCCTCTCAAAACTCA	
**MGAT3-A2-Fw**	GTTTTTGAGTTTTGAGAGGAATGG	PCR amplification of MGAT3-A2 fragment, forward primer was used for pyrosequencing
**MGAT3-A2-Rev**	[Btn]ACCCTCTTAAACCTACTCTCCTAC	
**MGAT3-F1_BS-alt-v2**	[Btn]GGAGGGGGTTGYGGAGGGGG	PCR amplification of MGAT3 A-TIS fragment, with only one biotinylated primer (forward or reverse)
**MGAT3-R_BS**	[Btn]CCTCCCCCCACCCCCACTTC	
**MGAT3-F1_BS-v4**	GGAGYGGGTATTTTTG	Pyrosequencing of MGAT3 A-TIS
**MGAT3-R_BS**	CCTCCCCCCACCCCCACTTC	
**LAMB1-A1-Fw**	GAAGTGGAGGGTTTAT	PCR amplification of LAMB1-A1 fragment
**LAMB1-A1-Rev**	[Btn]ATCAAATCTATCCAACAA	
**LAMB1-A1-seq**	TTGATTAGGGTGGG	Pyrosequencing of LAMB1-A1
**HNF1A-A1-Fw**	GGATAAGGGGGAGTTTTG	PCR amplification of HNF1A-A1 fragment
**HNF1A-A1-Rev**	[Btn]CTCCCCCAACCCATTAAA	
**HNF1A-A1-seq**	AAGGGGGAGTTT	Pyrosequencing of HNF1A-A1
**BACH2-A1-Fw**	TTATTGTGAATGGGGA	PCR amplification of BACH2-A1, forward primer was used for pyrosequencing
**BACH2-A1-Rev**	[Btn]ACTACTACTACTAAAAC	
**BACH2-A2-Fw**	GTTTTTATGGTATTTTTTAGG	PCR amplification of BACH2-A2, forward primer was used for pyrosequencing
**BACH2-A2-Rev**	[Btn]TCCCTCTACTATTCCAAAA	
**IL6ST-A1_Fw**	GAGAAGGATTTGATAGTGT	PCR amplification of IL6ST-A1 fragment
**IL6ST-A1-Rev**	[Btn]CCTCCTCACCTCAAAC	
**IL6ST-A1-seq**	AAGGATTTGATAGTGTTT	Pyrosequencing of IL6ST-A1

Supplementary Table 3. Sequences of gRNA molecules for each experiment.

gRNA molecule	Target site sequence (5'→3') including PAM (underlined)	Experiment
**MGAT3-sg01**	CATTCGCTGGGATATAGAATAGG	Activity profile of C-terminal fusion dSpCas9-TET1
**MGAT3-sg02**	CCCTGCACCTTCGACGATGGCGG	
**MGAT3-sg03**	ATGCCTTGTGCCTCGCACCGCGG	
**MGAT3-sg04**	CTGCTCTGTAGGCCCCAGAACGG	
**MGAT3-sg05**	GGACGCCTCTGAGCCCTGAGAGG	
**MGAT3-sg06**	TGGCCTAGAGCAAGGCCACGAGG	
**MGAT3-sg07**	CGGCACCGTGCACACATCACAGG	
**MGAT3-sg08**	AATCCCGGCCCAGGTTCACGCGG	
**LAMB1-sg01**	ACACATCCACCCTTTGTTGGGGG	
**LAMB1-sg02**	AGCAGCGAGAGCCTCCCTCCCGG	
**NT-gRNA**	GTAGGCGCGCCGCTCTCTAC	
**BACH2-F3f**	GCATTTTCTAGGAACGGGAAGGG	Localization by NLS
**MGAT3-sg03**	ATGCCTTGTGCCTCGCACCGCGG	Time-course evaluation of C-terminal fusion dSpCas9-TET1
**LAMB1-sg01**	ACACATCCACCCTTTGTTGGGGG	
**IL6ST-gRNA1**	GCCACCCCAGTCCCGCGGCGGGG	Time-course evaluation of N-terminal fusion DNMT3A-dSpCas9
**IL6ST-gRNA2**	ATCTGACAGTGTTCCGGAGCCGG	
**IL6ST-gRNA3**	CGCACGAACCCCTTGGCGCCAGG	
**IL6ST-gRNA4**	GCCAAGGGGTTCGTGCGCTGTGG	
**NT-gRNA**	GTAGGCGCGCCGCTCTCTAC	
**HNF1A-gRNA1**	AGAAGGCCCCCTGGACAAGGGGGAGT	Time-course evaluation of N-terminal fusion TET1-dSaCas9
**HNF1A-gRNA2**	TCCGTCTCGTCCTCGGAGCCCCGAGT	
**NT-gRNA**	GTAGGCGCGCCGCTCTCTAC	
**BACH2-sg01 (Sp)**	TGTATTTTGCTGGCGTCGAAGGG	Activity profile of N-terminal fusions of DNMT3A and TET1 catalytic domains with dSpCas9 and dSaCas9
**BACH2-sg02 (Sp)**	CTCTCCCTCCCGGCTGTTTACGG	
**BACH2-sg03 (Sp)**	GGGAGAGCACACATTCGGCGCGG	
**BACH2-sg06 (Sp)**	CCCATTCACAATAACTTTACGGG	
**BACH2-sg07 (Sp)**	AGTTATTGTGAATGGGGAGCGGG	
**BACH2-sg08 (Sp)**	AATGTAGCGATTGAGAGTGTGGG	
**BACH2-sgF3f (Sp)**	GCATTTTCTAGGAACGGGAAGGG	
**BACH2-sg08 (Sa)**	AATGTCAACAATGTAGCGATTGAGAGT	
**BACH2-sgF3f (Sa)**	GCATTTTCTAGGAACGGGAAGGGGGT	
**BACH2-sg01 (Sa)**	AAAGTTATTGTGAATGGGGAGCGGGT	
**BACH2-sg06 (Sa)**	GCCGGGCCGGGGGCAGGGCCGCGGGT	
**BACH2-sg08 (Sa)**	ATTGTATTTTGCTGGCGTCGAAGGGT	
**BACH2-sg09 (Sa)**	CAGCAGAGGGAGGAGGAGCAGAGAGT	
**MGAT3-sg01 (Sp)**	CATTCGCTGGGATATAGAATAGG	
**MGAT3-sg02 (Sp)**	CCCTGCACCTTCGACGATGGCGG	
**MGAT3-sg03 (Sp)**	ATGCCTTGTGCCTCGCACCGCGG	
**MGAT3-sg04 (Sp)**	CTGCTCTGTAGGCCCCAGAACGG	
**MGAT3-sg05 (Sp)**	GGACGCCTCTGAGCCCTGAGAGG	
**MGAT3-sg01 (Sa)**	TGGAGCACATTCGCTGGGATATAGAAT	
**MGAT3-sg05 (Sa)**	GGACGCCTCTGAGCCCTGAGAGGAAT	
**MGAT3-sg01 (Sa)**	GTAGACCAGCCCTAGGCAGCCCGGAT	
**MGAT3-sg02 (Sa)**	GGTGCGAGGCACAAGGCATTGTGGGT	
**MGAT3-sg05 (Sa)**	TGGCAGGAGAGTAGGCTCAAGAGGGT	
**NT-gRNA**	GTAGGCGCGCCGCTCTCTAC	
**HNF1A-gRNA1**	AGAAGGCCCCCTGGACAAGGGGGAGT	Simultaneous methylation and

**HNF1A-gRNA2**	TCCGTCTCGTCCTCGGAGCCCCGAGT	demethylation in HEK293 cells
**MGAT3-gRNA1**	GGAGCACATTCGCTGGGATATAGAAT	
**MGAT3-gRNA2**	GGTGCGAGGCACAAGGCATTGTGGGT	
**MGAT3-gRNA3**	GGACGCCTCTGAGCCCTGAGAGGAAT	
**MGAT3-gRNA4**	TGGCAGGAGAGTAGGCTCAAGAGGGT	
**MGAT3-gRNA5**	TCTGTGTGTCTGCTTGGGGCGTGGGT	
**MGAT3-gRNA6**	CCGGCTGGCGGGGGAGGGGAGGGGGT	
**BACH2-gRNA1**	AGTTATTGTGAATGGGGAGCGGG	
**BACH2-gRNA2**	AATGTAGCGATTGAGAGTGTGGG	
**BACH2-gRNA3**	CCGCGCCCTGCCCGCTTTTATGG	
**BACH2-gRNA4**	GGGAGAGCACACATTCGGCGCGG	
**IL6ST-gRNA1**	GCCACCCCAGTCCCGCGGCGGGG	
**IL6ST-gRNA2**	ATCTGACAGTGTTCCGGAGCCGG	
**IL6ST-gRNA3**	CGCACGAACCCCTTGGCGCCAGG	
**IL6ST-gRNA4**	GCCAAGGGGTTCGTGCGCTGTGG	
**NT-gRNA**	GTAGGCGCGCCGCTCTCTAC	
**HNF1A-gRNA1**	AGAAGGCCCCCTGGACAAGGGGGAGT	Simultaneous methylation and demethylation in BG1 cells
**HNF1A-gRNA2**	TCCGTCTCGTCCTCGGAGCCCCGAGT	
**MGAT3_112R**	GGCCGCTCGGCTGCACCCCGGGG	
**NT-gRNA**	GTAGGCGCGCCGCTCTCTAC	
**HNF1A-gRNA1 (TET1)**	AGAAGGCCCCCTGGACAAGGGGGAGT	Synergistic epigenetic regulation of *HNF1A*
**HNF1A-gRNA2 (TET1)**	TCCGTCTCGTCCTCGGAGCCCCGAGT	
**HNF1A-gRNA3 (VPR)**	AGGGAGCTATGGCCTGCGATGGG	
**NT-gRNA**	GTAGGCGCGCCGCTCTCTAC	
**IL6ST-gRNA1**	GCCACCCCAGTCCCGCGGCGGGG	Whole-genome methylation analysis
**IL6ST-gRNA2**	ATCTGACAGTGTTCCGGAGCCGG	
**IL6ST-gRNA3**	CGCACGAACCCCTTGGCGCCAGG	
**IL6ST-gRNA4**	GCCAAGGGGTTCGTGCGCTGTGG	
**MGAT3-gRNA1**	CATTCGCTGGGATATAGAATAGG	
**MGAT3-gRNA2**	CCCTGCACCTTCGACGATGGCGG	
**MGAT3-gRNA3**	ATGCCTTGTGCCTCGCACCGCGG	
**MGAT3-gRNA4**	CTGCTCTGTAGGCCCCAGAACGG	
**MGAT3-gRNA5**	GGACGCCTCTGAGCCCTGAGAGG	

Supplementary Table 4. Sequences of primers and custom oligonucleotides used for construction of modular toolbox

Primer/custom oligonucleotide	Sequence (5'→3')	Use
	**sgRNA modules**	
**tNS-Sg_G_XbaI-FW**	TAATCTCTAGAGGTCTCATGACGAGGGCCTATTTCCCATGATTCCTTC	Amplification of gRNA module for SpCas9
**tNS-Sg_G_NcoI-RE**	TTACTCCATGGTCTCATACCTCTCGAATTCAAAAAAGCACCGACTCGG	
**tNS-Sg_G_XbaI-FW**	TAATCTCTAGAGGTCTCATGACGAGGGCCTATTTCCCATGATTCCTTC	Amplification of gRNA module for SaCas9
**tNS-Sg_A_NcoI-RE**	TTACTCCATGGTCTCATACCCAAAAATCTCGCCAACAAGTTG	
	**Eukaryotic promoters**	

**tNS-Pro-C_PstI-Fw**	TTAATCTGCAGGGTCTCAGGTACAGACAAATGGCTCTAGAGGTAC CCGTTACATAACTT	Amplification of CAG promoter
**tNS-Pro-C_XhoI-RE**	TTACTCTCGAGGGTCTCTATGGTGGCAGCGCTCTAGAACCGGTCTG AAAAAAAGTGATTTCAGGCAGGTGCTCCAGG	
**tNS-Pro_SV40_PstI-FW**	TAATCTGCAGGGTCTCAGGTAGCTGTGGAATGTGTGTCAGTTAGG GTG	Amplification of SV40 promoter
**tNS-Pro_SV40_XhoI-RE**	TAATCTCGAGGGTCTCTATGGTGGCAGCGCTCTAGAACCGGTGCTT TTTGCAAAAGCCTAGGCCTCC	
**tNS-Pro_E_PstI-FW**	TAATCTGCAGGGTCTCGGGTATAGGTCTTGAAAGGAGTGGGAATT GGC	Amplification of EFS promoter
**tNS-Pro_E_XhoI-RE**	TTACTCTCGAGGGTCTCAATGGTGGCAGCGCTCTAGAACCGGTCCT GTGTTCTGG	
	**Effector domains**	
**tNS-ED_D_Hind3-FW**	TTAAAAGCTTGGTCTCCGGAGGCGGGAGCGGATCCCCCTC	Amplification of DNMT3A effector domain
**tNS-ED_D_SacI-RE**	TTACGAGCTCGGTCTCTCGAATGGCCGGCCGGACACACACG	
**FD_c-NLS-NP-S**	GCGCGGTCTCAGGAGAGAGACGTTGCGTCTCATTCGGTTCCGGAA AGAGGCCAGCAGCTACAAAGAAAGCTGGACAGGCAAAAAAGAAA AAGTCAAGCTTCGAGAGACC	Construction of C-terminal NLS on effector domains
**FD_c-NLS-NP-A**	TCGAGGTCTCTCGAAGCTTGACTTTTTCTTTTTTGCCTGTCCAGCTTT CTTTGTAGCTGCTGGCCTCTTTCCGGAACCGAATGAGACGCAACGT CTCTCTCCTGAGACC	
**tNS-ED_T_XbaI-FW**	TTAATCTAGAGGTCTCCGGAGGCGGGAGCGGATCCCTGC	Amplification of TET1 effector domain
**tNS-ED_T_Acc65I-RE**	TTACGGTACCGGTCTCTCGAATGGCCGGCCGACCCAATGG	
**TET1_noBbsI-1_S**	CTTCTCCTGGTCCCCAAAGACTGCTTCAGCC	BbsI restriction sites mutagenesis in TET1
**TET1_noBbsI-1_A**	GGCTGAAGCAGTCTTTGGGGACCAGGAGAAG	
**TET1_noBbsI-2_S**	GATGCCTTCGGGAAGGCTCAGTGGTGCCAAT	
**TET1_noBbsI-2_A**	ATTGGCACCACTGAGCCTTCCCGAAGGCATC	
**TET1_noBsaI-S**	CCAACCTTAGGGAGTAACACTGAAACCGTGCAACCT	BsaI restriction site mutagenesis in TET1
**TET1_noBsaI-A**	AGGTTGCACGGTTTCAGTGTTACTCCCTAAGGTTGG	
**TET1_H1671Y_D1673A _S**	GACTTCTGTGCTCATCCCTACAGGGCCATTCACAACATGAATAA	Mutagenesis of active site in TET1 (H1671Y, D1673A)
**TET1_H1671Y_D1673A _A**	TTATTCATGTTGTGAATGGCCCTGTAGGGATGAGCACAGAAGTC	
**tNS-ED_V_Hind3-FW**	ATTAAAAGCTTGGTCTCAGGAGGCGGCAGCGAGGCCAGCGGTTCC GGACG	Amplification of VPR activation domain
**tNS-ED_V_XhoI-RE**	TTACTCTCGAGGGTCTCACGAAAAACAGAGATGTGTCGAAGATGG ACAGTCCTGTGC	
**VPR-noEsp3I- S**	ATCCCGATGAAGAGACAAGCCAGGCTGTCAAAG	Esp3I restriction site mutagenesis in VPR
**VPR_noEsp3I-A**	CTTTGACAGCCTGGCTTGTCTCTTCATCGGGAT	
**N-FD-S**	AGCTTGGTCTCACCATGGTGCCAAAAAAGAAGAGAAAGGTAGGC GGAGTAGTCTTCTATAGAAGACATTTCGGTGGAGCGAGACCC	Construction of effector domains for N-terminal fusion with dCas9
**N-FD-A**	TCGAGGGTCTCGCTCCACCGAAATGTCTTCTATAGAAGACTACTCC GCCTACCTTTCTCTTCTTTTTTGGCACCATGGTGAGACCA	
	**dCas9 modules**	
**tNS-Cas_N_Hind3-FW**	ATTACAAGCTTGGTCTCACCATGGACTATAAGGACCACGACGG	Amplification of dSpCas9 module
**tNS-Cas_N_XhoI-RE**	TTACTCTCGAGGGTCTCACTCCGCCCTTTTTCTTTTTGGCCTGTCCG G	
**tNS-Cas_A_XbaI-FW**	ATTACTCTAGAGGTCTCACCATGGCCCCAAAGAAG	Amplification of dSaCas9 module
**tNS-Cas_A_XhoI-RE**	TTACTCTCGAGGGTCTCACTCCGCCCTTTTTCTTTTTTGCCTGGCCG G	

**tNS-Cas_A_XbaI-FW**	ATTACTCTAGAGGTCTCACCATGGCCCCAAAGAAG	Amplification of dSaCas9 module with 3×HA-FLAG
**tNS-Cas_A_XhoI+FLAG-RE**	TTACTCTCGAGGGTCTCACTCCGCCGAGCTCTAGGAATTCTTCAGCGTA	
**LNK_trpzip2_G4Sx2-S**	GATCCAGCTGGACGTGGGAGAACGGGAAATGGACCTGGAAGGCTAGCGGCGGAGGTGGCAGCGGTGGAGTGAGACCC	Construction of Trip-zip linker (TZ2)
**LNK_trpzip2_G4Sx2-A**	TCGAGGGTCTCACTCCACCGCTGCCACCTCCGCCGCTAGCCTTCCAGGTCCATTTCCCGTTCTCCCACGTCCAGCTG	
**N-C9-S**	AGCTTGGTCTCTGGAGGTGGCTCCATGTGTCTTCGATGCAGAAGACGTGGAGATTTCGTGAGACCC	Construction of dCas9 module for N-terminal fusion
**N-C9-A**	TCGAGGGTCTCACGAAATCTCCACGTCTTCTGCATCGAAGACACATGGAGCCACCTCCAGAGACCA	
	**Selection markers and dual marker system**	
**AR_Puro_NcoI_Fw**	GAGAGAGCCATGGTGACCGAGTACAAGCCCACGG	Amplification of PuroR module
**AR_Puro_KasI_Re**	GAGAGAGGCGCCGGCACCGGGCTTGCG	
**PuroM_C210T_S**	GCGGACGACGGCGCTGCGGTGGCGGTCTGG	KasI restriction sites mutagenesis in PuroR module
**PuroM_C210T_A**	CCAGACCGCCACCGCAGCGCCGTCGTCCGC	
**PuroM_G339T_S**	GGAAGGCCTCCTGGCTCCGCACCGGCCCAAG	
**PuroM_G339T_A**	CTTGGGCCGGTGCGGAGCCAGGAGGCCTTCC	
**Puro_noBsaI-S**	CTTCCTGGAGACATCCGCGCCCCGC	BsaI restriction site mutagenesis in PuroR module
**Puro_noBsaI-A**	GCGGGGCGCGGATGTCTCCAGGAAG	
**FP_Ruby_NcoI_Fw**	GAGAGAGCCATGGTGTCTAAGGGCGAAGAGCTGA	Amplification of mRuby3 module
**FP_KasI_Rev**	GAGAGAGGGCGCCCTTGTACAGCTCGTCCATGCC	
**G414A_A**	CCAATGGTCCCGTGATGCAGAAAAAGACCAAGGGT	BpiI restriction site mutagenesis in mRuby3 module
**G414A_S**	ACCCTTGGTCTTTTTCTGCATCACGGGACCATTGG	
**FP_CC_NcoI_Fw**	GAGAGAGCCATGGTGAGCAAGGGCG	Amplification of mClover3 module
**FP_KasI_Rev**	GAGAGAGGGCGCCCTTGTACAGCTCGTCCATGCC	
**pFP-Entry_S**	ATCATACTGCAGTAGGTCTTGAAAGGAGTGGGAATTGGCTCCGGTGCCCGTCAGTGGGCAGAGCGCACATCGCCCACAGTCCCCGAGAAGTTGGGGGGAGGGGTCGGCAATTGATCCGGTGCCTAGAGAAGGTGGCGCGGGGTAAACTGGGAAAGTGATGTCGTGTACTGGCTCCGCCTTTTTCCCGAGGGTGGGGGAGAACCGTATATAAGTGCAGTAGTCGCCGTGAACGTTCTTTTTCGCAACGGGTTTGCCGCCAGAACACAGGCACCAGGTCTTCAGGAGGTAAAACGACGGCCAGTAATTAATGTGAGTTAGCTCACTCATTAGGCACCCCAGGCTTTACACTTTATGCTTCCGGCTCGTATGTTGTGTGGAATTGTGAGCGGATAACAATTTCACACAGGAGGCTGCCACCATGGGAGACCGATATCCGCTCTAGAACTAGTGGATCGGTCTCGGCGCCGGCTAGCTTGAGTAACTAGGACGAACAATAAGGCCTCCCTAACGGGGGGCCTTTTTTATTGATAACAAAAGTCATAGCTGTTTCCTGCCGTGGAAGACCTGTTTGCAGCCTCGACTGTGCCTTCTAGTTGCCAGCCATCTGTTGTTTGCCCCTCCCCCGTGCCTTCCTTGACCCTGGAAGGTGCCACTCCCACTGTCCTTTCCTAATAAAATGAGGAAATTGCATCGCATTGTCTGAGTAGGTGTCATTCTATTCTGGGGGGTGGGGTGGGGCAGGACAGCAAGGGGGAGGATTGGGAAGAGAATAGCAGGCATGCTCGAGACTAAC	Construction of pUK21_FP_entry plasmid for cloning of fluorescence and selection modules
**pFP-Entry_A**	GTTAGTCTCGAGCATGCCTGCTATTCTCTTCCCAATCCTCCCCCTTGCTGTCCTGCCCCACCCCACCCCCCAGAATAGAATGACACCTACTCAGACAATGCGATGCAATTTCCTCATTTTATTAGGAAAGGACAGTGG	

	GAGTGGCACCTTCCAGGGTCAAGGAAGGCACGGGGGAGGGGCA AACAACAGATGGCTGGCAACTAGAAGGCACAGTCGAGGCTGCAA ACAGGTCTTCCACGGCAGGAAACAGCTATGACTTTTGTTATCAATA AAAAAGGCCCCCCGTTAGGGAGGCCTTATTGTTCGTCCTAGTTACT CAAGCTAGCCGGCGCCGAGACCGATCCACTAGTTCTAGAGCGGAT ATCGGTCTCCCATGGTGGCAGCCTCCTGTGTGAAATTGTTATCCGC TCACAATTCCACACAACATACGAGCCGGAAGCATAAAGTGTAAAG CCTGGGGTGCCTAATGAGTGAGCTAACTCACATTAATTACTGGCCG TCGTTTTACCTCCTGAAGACCTGGTGCCTGTGTTCTGGCGGCAAAC CCGTTGCGAAAAAGAACGTTCACGGCGACTACTGCACTTATATACG GTTCTCCCCCACCCTCGGGAAAAAGGCGGAGCCAGTACACGACAT CACTTTCCCAGTTTACCCCGCGCCACCTTCTCTAGGCACCGGATCAA TTGCCGACCCCTCCCCCCAACTTCTCGGGGACTGTGGGCGATGTGC GCTCTGCCCACTGACGGGCACCGGAGCCAATTCCCACTCCTTTCAA GACCTACTGCAGTATGAT	
**pFP-T2A_S**	ATCTCATACGTCTCCAGCTCTGCAGAGGAGGACAAGCTTATGGTCT CATTCGGCAGTGGAGAGGGCAGAGGAAGTCTGCTAACATGCGGT GACGTCGAGGAGAATCCTGGCCCAGCCATGGTGTCTTCGATATCC GCTCTAGAACTAGTGGATCTGAAGACCGGCGCCGGCTAGCATCTC GAGTAACGCTAGAGACCGAGCTCTCGATGAGACGACTACTAC	Construction of pUK21_FP_T2A
**pFP-T2A_A**	GTAGTAGTCGTCTCATCGAGAGCTCGGTCTCTAGCGTTACTCGAGA TGCTAGCCGGCGCCGGTCTTCAGATCCACTAGTTCTAGAGCGGATA TCGAAGACACCATGGCTGGGCCAGGATTCTCCTCGACGTCACCGC ATGTTAGCAGACTTCCTCTGCCCTCTCCACTGCCGAATGAGACCAT AAGCTTGTCCTCCTCTGCAGAGCTGGAGACGTATGAGAT	
**T2A_FW**	ATCTCATACGTCTCCAGCTCTGC	Amplification of pFP-T2A
**T2A_RE**	GTAGTAGTCGTCTCATCGAGAGCTC	
**T2A_X-FW**	TTCAGTCTCGTCTCCAGCTCTGCAGAGGAGGACAAGC	Amplification of pFP-T2A for dual marker system
**T2A_X-RE**	ACTTGATACGTCTCATCGAGGTCTCTAGTACGGCGCCGGTCTTCAG ATCCAC	
**C9seq3**	GGACATCCAGAAAGCCCAGG	Amplification of X-P2A from Gblock-BB
**C9seq4**	GAAGTCCAAGCTGGTGTCCG	
**pUK21_KasI-BamHI-S**	GCGCATGTAAAACGACGGCCAGTTAATACGACTCACTATAGGTAG	KasI restriction site removal from pUK21gg
**pUK21_KasI-BamHI-A**	GATCCTACCTATAGTGAGTCGTATTAACTGGCCGTCGTTTTACAT	
	**Eukaryotic terminators**	
**tNS-Ter-H_Hind3-FW**	TTAATAAGCTTGGTCTCTCGCTAGCCTCGACTGTGCCTTCTAGTTGC C	Amplification of Bgh terminator
**tNS-Ter-H_NcoI-RE**	TTACTCCATGGTCTCAACCTCATGCCTGCTATTCTCTTCCCAATCCTC	
**tNS-Ter-SV40_PstI-FW**	TAATCTGCAGAAGCTTGGTCTCTCGCTGTTGTTAACTTGTTTATTGC AGCTTATAATGG	Amplification of SV40 terminator
**tNS-Ter-SV40_XhoI-RE**	TAATCTCGAGGTCTCAACCTCCAGTTGATCCAGACATGATAAGATA CATTG	
	**Backbone vector**	
**pUC19-FW**	ATCGGTACCGGAACCCCTATTTGTTTATTTTTCT	Construction of backbone plasmid (pBackBone-BZ)
**pUC19-RE**	TATACCGGTCATGTGAGCAAAAGGCCAGC	
**Gblock-BB_S**	TACATGGATCCTGAACCCCGACAACAGCGACCGTCTCAGTACACTG CAGGCCGGCATCCCGCCCCTAACTCCGCCCAGTTCCGCCCATTCTC CGCCTCATGGCTGACTAATTTTTTGCGTCTTCGGTACCGAAGACGC TTTTTTATTTATGCAGAGGCCGAGGCCGCCTCGGCCTCTGAGCTAT TCCAGAAGTAGTGAGGAGGCTTTTTTGGAGGCCGGCGACGTCTAC ATGAGCTCATTGAGATGCATGCTTTGCATACTTCTGCCTGCTGGGG AGCCTGGGGACTTTCCACACCTGGTTGCTGACTAATTGAGATCAGG AGGTAAAACGACGGCCAGTGGTACCTATGAACAGTTACTAAGAGT	

	CGACTGACTGAGACCAATTAATGTGAGTTAGCTCACTCATTAGGCA<br>CCCCAGGCTTTACACTTTATGCTTCCGGCTCGTATGTTGTGTGGAAT<br>TGTGAGCGGATAACAATTTCACACAGGAGGCTGCCACCATGGTGA<br>TGATTACGGATTCACTGGCAGTGGTCCTGCAACGTCGTGACTGGG<br>AAAACCCTGGCGTTACCCAACTTAATCGCCTTGCAGCACATCCGCC<br>TTTCGCCAGCTGGCGTAATAGCGAAGAGGCCCGCACCGATCGCCC<br>TTCCCAACAGCTGCGCAGCCTGAATGGCGCCTAAAGCTAGCTTGA<br>GTAACTAGGACGAACAATAAGGCCTCCCTAACGGGGGGCCTTTTT<br>TATTGATAACAAAAGGTCTCTAGGTTCTAGATATACTTCATGTAAG<br>ACACTCGAGGTCATAGCTGTTTCCTGCCGTGGTCCGGTGAGACGG<br>GGATGCGGAAGGTCAGGATGGACATCCAGAAAGCCCAGGGAAGA<br>CTAAGCTTATGGTCTCATACTTGGCAGTGGAGCCACGAACTTCTCT<br>CTGTTAAAGCAAGCTGGCGACGTGGAAGAAAACCCCGGTCCTGCC<br>ATGGTGTCTTCCGGACACCAGCTTGGACTTCAGATCTTACAT	
**Gblock-BB_A**	ATGTAAGATCTGAAGTCCAAGCTGGTGTCCGGAAGACACCATGGC<br>AGGACCGGGGTTTTCTTCCACGTCGCCAGCTTGCTTTAACAGAGAG<br>AAGTTCGTGGCTCCACTGCCAAGTATGAGACCATAAGCTTAGTCTT<br>CCCTGGGCTTTCTGGATGTCCATCCTGACCTTCCGCATCCCCGTCTC<br>ACCGGACCACGGCAGGAAACAGCTATGACCTCGAGTGTCTTACAT<br>GAAGTATATCTAGAACCTAGAGACCTTTTGTTATCAATAAAAAAGG<br>CCCCCCGTTAGGGAGGCCTTATTGTTCGTCCTAGTTACTCAAGCTA<br>GCTTTAGGCGCCATTCAGGCTGCGCAGCTGTTGGGAAGGGCGATC<br>GGTGCGGGCCTCTTCGCTATTACGCCAGCTGGCGAAAGGCGGATG<br>TGCTGCAAGGCGATTAAGTTGGGTAACGCCAGGGTTTTCCCAGTC<br>ACGACGTTGCAGGACCACTGCCAGTGAATCCGTAATCATCACCATG<br>GTGGCAGCCTCCTGTGTGAAATTGTTATCCGCTCACAATTCCACAC<br>AACATACGAGCCGGAAGCATAAAGTGTAAAGCCTGGGGTGCCTAA<br>TGAGTGAGCTAACTCACATTAATTGGTCTCAGTCAGTCGACTCTTA<br>GTAACTGTTCATAGGTACCACTGGCCGTCGTTTTACCTCCTGATCTC<br>AATTAGTCAGCAACCAGGTGTGGAAAGTCCCCAGGCTCCCCAGCA<br>GGCAGAAGTATGCAAAGCATGCATCTCAATGAGCTCATGTAGACG<br>TCGCCGGCCTCCAAAAAAGCCTCCTCACTACTTCTGGAATAGCTCA<br>GAGGCCGAGGCGGCCTCGGCCTCTGCATAAATAAAAAAGCGTCTT<br>CGGTACCGAAGACGCAAAAAATTAGTCAGCCATGAGGCGGAGAA<br>TGGGCGGAACTGGGCGGAGTTAGGGGCGGGATGCCGGCCTGCAG<br>TGTACTGAGACGGTCGCTGTTGTCGGGGTTCAGGATCCATGTA	
**C9seq1**	TGAACCCCGACAACAGCGAC	Amplification of Gblock-BB
**C9seq2**	ATCCTGACCTTCCGCATCCC	
**pUC19bla_noBsaI-S**	GGAGCCGGTGAGCGTGGAAGCCGCGGTATCATTGCAGC	BsaI restriction site mutagenesis in backbone plasmid (pBackBone-BZ)
**pUC19bla_noBsaI-A**	GCTGCAATGATACCGCGGCTTCCACGCTCACCGGCTCC	
	**Multi-guide system**	
**pFUS-newNgoMIV-A**	GTCGAGGCATTTCTGTGCCGGCTGGTCTAGACGTC	Construction of pUS21gg vector
**pFUS-newNgoMIV-S**	GACGTCTAGACCAGCCGGCACAGAAATGCCTCGAC	
**pFUS-noNgoMIV-A**	CTGATACTGGGCTGGCAGGCGCTCC	
**pFUS-noNgoMIV-S**	GGAGCGCCTGCCAGCCCAGTATCAG	
**pFUS-noXhoI-A**	GGTCATGGGTGGCTCTAGGGTTATTTGCCGA	
**pFUS-noXhoI-S**	TCGGCAAATAACCCTAGAGCCACCCATGACC	
**KAN_NgoMIV-RE**	ATATTGCCGGCTTCGAAAGGGCCTCGTGATACGC	Construction of pUK21gg vector
**KAN_SapI-FW**	CGTATTGGGCGCTCTTCCG	
**KanR_noEsp3I-A**	GATCGCGTATTTCGGCTCGCTCAGGCGCA	
**KanR_noEsp3I-S**	TGCGCCTGAGCGAGCCGAAATACGCGATC	
**sgM1-S**	GCGCCCGTCTCATGACTTCTAGATGACACATCCATGGAGGTATGAG<br>ACGC	Construction of pSgMxA or

**sgM1-A**	TCGAGCGTCTCATACCTCCATGGATGTGTCATCTAGAAGTCATGAGACGG	pSgMxG plasmids (x = 1, 2, 3, 4, 5, 6)
**sgM2-S**	GCGCCCGTCTCAGGTATTCTAGATGACACATCCATGGACCATTGAGACGC	
**sgM2-A**	TCGAGCGTCTCAATGGTCCATGGATGTGTCATCTAGAATACCTGAGACGG	
**sgM3-S**	GCGCCCGTCTCACCATTTCTAGATGACACATCCATGGAGGAGTGAGACGC	
**sgM3-A**	TCGAGCGTCTCACTCCTCCATGGATGTGTCATCTAGAAATGGTGAGACGG	
**sgM4-S**	GCGCCCGTCTCAGGAGTTCTAGATGACACATCCATGGATTCGTGAGACGC	
**sgM4-A**	TCGAGCGTCTCACGAATCCATGGATGTGTCATCTAGAACTCCTGAGACGG	
**sgM5-S**	GCGCCCGTCTCATTCGTTCTAGATGACACATCCATGGACGCTTGAGACGC	
**sgM5-A**	TCGAGCGTCTCAAGCGTCCATGGATGTGTCATCTAGAACGAATGAGACGG	
**sgM6-S**	GCGCCCGTCTCACGCTTTCTAGATGACACATCCATGGAAGGTTGAGACGC	
**sgM6-A**	TCGAGCGTCTCAACCTTCCATGGATGTGTCATCTAGAAAGCGTGAGACGG	
**XbaI_B_C9seq1_A_NcoI-S**	CTAGATGACTGAGACCTGAACCCCGACAACAGCGACGGTCTCTGGTACGC	
**XbaI_B_C9seq1_A_NcoI-A**	CATGGCGTACCAGAGACCGTCGCTGTTGTCGGGGTTCAGGTCTCAGTCAT	
**SgMult-S**	GCGCCGGTCTCATGACTGACTGAGACGTCTAGACACCAGGTCTTCTGACACATGAAGACCTGTTTC	Construction of pSgx1-pSgx6 plasmids
**SgMult-A**	CATGGAAACAGGTCTTCATGTGTCAGAAGACCTGGTGTCTAGACGTCTCAGTCAGTCATGAGACCG	
**SgMx1-S**	CATGGCGTCTCAGGTAGGTATGAGACCC	
**SgMx1-A**	TCGAGGGTCTCATACCTACCTGAGACGC	
**SgMx2-S**	CATGGCGTCTCACCATTGGTATGAGACCC	
**SgMx2-A**	TCGAGGGTCTCATACCAATGGTGAGACGC	
**SgMx3-S**	CATGGCGTCTCAGGAGGGTATGAGACCC	
**SgMx3-A**	TCGAGGGTCTCATACCCTCCTGAGACGC	
**SgMx4-S**	CATGGCGTCTCATTCGGGTATGAGACCC	
**SgMx4-A**	TCGAGGGTCTCATACCCGAATGAGACGC	
**SgMx5-S**	CATGGCGTCTCACGCTGGTATGAGACCC	
**SgMx5-A**	TCGAGGGTCTCATACCAGCGTGAGACGC	
**SgMx6-S**	CATGGCGTCTCAAGGTGGTATGAGACCC	
**SgMx6-A**	TCGAGGGTCTCATACCACCTTGAGACGC	
	**Secondary cassette**	
**M14-S**	GCGCAGGTCTCACCATGGCACCAGGTCTTCTGAACCCCGACAACAGCGACGAAGACCTGTTTGGCGCCGGCTAGCCGCTAGAGACC	Construction of secondary cassette
**M14-A**	TCGAGGTCTCTAGCGGCTAGCCGGCGCCAAACAGGTCTTCGTCGCTGTTGTCGGGGTTCAGAAGACCTGGTGCCATGGTGAGACCT	
**BB_2nd_Cassette-S**	CGGTACGAGACGTGAACCCCGACAACAGCGACCGTCTCCAGGTGAGCT	
**BB_2nd_Cassette-A**	CACCTGGAGACGGTCGCTGTTGTCGGGGTTCACGTCTCGTACCGACGT	

Supplementary Table 5. Quantitative real-time PCR primers used for evaluation of TET1-dSaCas9 presence through time

Primer name	Sequence (5' $\rightarrow$ 3')	Use
**SaCas9_Fw**	CCGCCCGGAAAGAGATTATT	Detection of SaCas9
**SaCas9_Rev**	CGGAGTTCAGATTGGTCAGTT	
**SpCas9_Fw**	TGCCCCAAGTGAATATCGTG	Detection of SpCas9
**SpCas9_Rev**	GACTTGCCCTTTTCCACTTTG	
**RAG1_Fw**	TGTTGACTCGATCCACCCCA	Endogenous control for Cas9 plasmid DNA normalization
**RAG1_Rev**	TGAGCTGCAAGTTTGGCTGAA	
**GAPDH_Fw**	AGGGCTGCTTTTAACTCTGGT	Endogenous control for Cas9 expression normalization
**GAPDH_Rev**	CCCCACTTGATTTTGGAGGGA	

Supplementary Table 6. Annotation of glycan peaks with corresponding glycan structures according to the results of mass spectrometry. A typical chromatogram is given in Supplementary Figure S7. In the first column labelled GP (glycan peak), "CONT" denotes contamination between peaks GP5 and GP6, resulting in the inability to quantitate the affected glycan structures (see also: Supplementary Figure S7).

**GP**	**Structure**	**RT (min)**	**Theoretical mass [MH]$^+$**	**Measured mass [MH]$^+$**	**MS/ MS**	**major structure in peak**
1	(Hex)$_2$ (HexNAc)$_2$	3.11	968.456	968.461	yes	yes
2	(Hex)$_2$ (HexNAc)$_2$ (Deoxyhexose)$_1$	3.75	1114.514	1114.527	yes	yes
3	(Hex)3 (HexNAc)$_2$	4.54	1130.509	1130.523	yes	yes
4	(Hex)3 (HexNAc)$_2$ (Deoxyhexose)$_1$	5.31	1276.567	1276.581	yes	yes
5	(Hex)$_4$ (HexNAc)$_2$	6.02	1292.562	1292.576	yes	yes
CONT	(Hex)$_4$ (HexNAc)$_2$ (Deoxyhexose)$_1$	6.85	1438.620	1438.627	yes	yes
	(Hex)$_1$ (HexNAc)$_1$ + (Man)$_3$(GlcNAc)$_2$	7.3	1495.641	1495.646	no	no
	(HexNAc)$_2$ (Deoxyhexose)$_1$ + (Man)$_3$(GlcNAc)$_2$		1682.726	1682.732	yes	yes
6	(Hex)$_2$ + (Man)$_3$(GlcNAc)$_2$	8.06	1454.615	1454.620	yes	yes
7	(HexNAc)$_3$ (Deoxyhexose)$_1$ + (Man)$_3$(GlcNAc)$_2$	8.71	1885.805	1885.819	yes	yes
8	(Hex)$_2$ (Deoxyhexose)$_1$ + (Man)$_3$(GlcNAc)$_2$	8.91	1600.673	1600.697	yes	yes
	(Hex)$_2$ (HexNAc)$_1$ + (Man)$_3$(GlcNAc)$_2$		1657.694	1657.699	yes	yes
9	(HexNAc)$_4$ (Deoxyhexose)$_1$ + (Man)$_3$(GlcNAc)$_2$	9.52	2088.885	2088.915	no	no
	(Hex)$_1$ (HexNAc)$_3$ (Deoxyhexose)$_1$ + (Man)$_3$(GlcNAc)$_2$		2047.858	2047.867	no	no
	(Hex)$_2$ (HexNAc)$_2$ + (Man)$_3$(GlcNAc)$_2$		1860.774	1860.783	yes	yes
10	(Hex)$_3$ + (Man)$_3$(GlcNAc)$_2$	9.9	1616.668	1616.678	yes	yes

11	(Hex)$_2$ (HexNAc)$_2$ (Deoxyhexose)$_1$ + (Man)$_3$(GlcNAc)$_2$	10.45	2006.832	2006.841	yes	yes
	(Hex)$_2$ (HexNAc)$_3$ + (Man)$_3$(GlcNAc)$_2$		2063.853	2063.879	yes	no
12	(Hex)$_2$ (HexNAc)$_1$ (Deoxyhexose)$_2$ + (Man)$_3$(GlcNAc)$_2$	10.74	1949.810	1949.821	yes	yes
13	(Hex)$_3$ (HexNAc)$_1$ + (Man)$_3$(GlcNAc)$_2$	10.92	1819.747	1819.759	yes	yes
	(Hex)$_2$ (HexNAc)$_2$ (Deoxyhexose)1 + (Man)3(GlcNAc)2		2006.832	2006.841	no	no
14	(Hex)2 (HexNAc)3 (Deoxyhexose)$_1$ + (Man)$_3$(GlcNAc)$_2$	11.2	2209.911	2209.935	yes	yes
	(Hex)$_3$ (HexNAc)$_2$ + (Man)$_3$(GlcNAc)$_2$		2022.826	2022.851	yes	no
15	(Hex)$_4$ + (Man)$_3$(GlcNAc)$_2$	11.8	1778.720	1778.737	yes	yes
	(Hex)$_3$ (HexNAc)$_1$ (Deoxyhexose)$_1$ + (Man)$_3$(GlcNAc)$_2$		1965.805	1965.831	no	no
	(Hex)$_3$ (HexNAc)$_2$ (Deoxyhexose)$_1$ + (Man)$_3$(GlcNAc)$_2$		2168.884	2168.911	yes	no
16	(Hex)$_2$ (HexNAc)$_3$ (Deoxyhexose)$_2$ + (Man)$_3$(GlcNAc)$_2$	12.16	2355.969	2356.000	yes	yes
17	(Hex)$_3$ (HexNAc)$_2$ (Deoxyhexose)$_1$ + (Man)$_3$(GlcNAc)$_2$	12.55	2110.842	2110.867	yes	no
	(Hex)$_2$ (HexNAc)$_2$ (NeuAc)$_1$ + (Man)$_3$(GlcNAc)$_2$		2151.869	2151.905	yes	yes
18	(Hex)$_4$ (HexNAc)$_2$ + (Man)$_3$(GlcNAc)$_2$	13.18	2184.879	2184.915	yes	yes
	(Hex)$_3$ (HexNAc)$_2$ (Deoxyhexose)$_2$ + (Man)$_3$(GlcNAc)$_2$		2314.942	2314.977	yes	yes
	(Hex)$_5$ + (Man)$_3$(GlcNAc)$_2$		1940.773	1940.803	yes	no
	(Hex)$_2$ (HexNAc)$_2$ (NeuAc)$_2$ + (Man)$_3$(GlcNAc)$_2$		2442.964	2442.999	no	no
19	(Hex)$_5$ + (Man)$_3$(GlcNAc)$_2$	13.56	1940.773	1940.803	yes	yes
20	(Hex)$_4$ (HexNAc)$_2$ + (Man)$_3$(GlcNAc)$_2$	13.82	2184.879	2184.905	no	no
	(Hex)$_4$ (HexNAc)$_2$ (Deoxyhexose)$_1$ + (Man)$_3$(GlcNAc)$_2$		2330.937	2330.975	yes	yes
21	(Hex)$_2$ (HexNAc)$_2$ (NeuAc)$_2$ + (Man)$_3$(GlcNAc)$_2$	14.07	2442.964	2442.997	yes	yes
	(Hex)$_3$ (HexNAc)$_3$ (NeuAc)$_1$ + (Man)3(GlcNAc)$_2$		2517.001	2517.035	yes	no
22	(Hex)$_3$ (HexNAc)$_3$ (NeuAc)$_1$ + (Man)$_3$(GlcNAc)$_2$	14.86	2517.001	2517.036	yes	no
	(Hex)$_6$ + (Man)$_3$(GlcNAc)$_2$		2102.826	2102.867	yes	yes
	(Hex)$_2$ (HexNAc)$_2$ (NeuAc)$_2$ + (Man)$_3$(GlcNAc)$_2$		2442.964	2443.007	no	no
23	(Hex)$_3$ (HexNAc)$_3$ (NeuAc)$_2$ + (Man)$_3$(GlcNAc)$_2$	15.41	2808.097	2808.142	yes	yes
24	(Hex)$_3$ (HexNAc)$_3$ (NeuAc)$_2$ + (Man)$_3$(GlcNAc)$_2$	16.11	2808.097	2808.133	yes	yes
	(Hex)$_7$ + (Man)$_3$(GlcNAc)$_2$		2264.879	2264.919	no	no
25	(Hex)$_4$ (HexNAc)$_4$ (Deoxyhexose)$_2$ + (Man)$_3$(GlcNAc)$_2$	16.66	2883.154	2883.203	no	no
	(Hex)3 (HexNAc)$_3$ (Deoxyhexose)$_1$ (NeuAc)$_2$ + (Man)$_3$(GlcNAc)$_2$		2954.155	2954.199	no	no
	(Hex)$_3$ (HexNAc)$_3$ (NeuAc)$_3$ + (Man)$_3$(GlcNAc)$_2$		3099.192	3099.237	yes	yes
26	(Hex)$_3$ (HexNAc)$_3$ (NeuAc)$_2$ + (Man)$_3$(GlcNAc)$_2$	16.87	2808.097	2808.143	yes	yes
27	(Hex)$_3$ (HexNAc)$_3$ (NeuAc)$_3$ + (Man)$_3$(GlcNAc)$_2$	17.36	3099.192	3099.247	yes	yes

28	$(Hex)_3$ $(HexNAc)_3$ $(NeuAc)_3$ + $(Man)_3(GlcNAc)_2$	18.06	3099.192	3099.251	yes	yes
29	$(Hex)_3$ $(HexNAc)_3$ $(NeuAc)_4$ + $(Man)_3(GlcNAc)_2$	18.44	3390.287	3390.340	yes	yes
30	$(Hex)_3$ $(HexNAc)_3$ $(NeuAc)_3$ + $(Man)_3(GlcNAc)_2$	18.79	3099.192	3099.244	yes	yes
31	$(Hex)_3$ $(HexNAc)_3$ $(NeuAc)_4$ + $(Man)_3(GlcNAc)_2$	19.1	3390.287	3390.352	yes	yes

Supplementary Table 7. Wilcoxon rank sum p-values to assess the effect of potential covariates on principal components after probe filtering and normalization. Covariates contributing significant effects [Wilcoxon p-values less than the calculated Bonferroni limit (0.05/40 = 0.0013)] are highlighted in red.

	PC1	PC2	PC3	PC4	PC5	PC6	PC7	PC8	PC9	PC10
**Sentrix ID**	0.340	0.002	0.329	0.086	0.559	0.323	0.432	0.472	0.804	0.374
**Sentrix Position**	0.806	0.998	0.482	0.757	0.362	0.063	0.523	0.822	0.553	0.355
**Sample Well**	0.457	0.457	0.457	0.457	0.457	0.457	0.457	0.457	0.457	0.457
**Biological Replicate**	0.123	0.000	0.796	0.123	0.684	0.579	0.684	0.123	0.796	0.393
**Technical Replicate**	0.340	0.002	0.329	0.086	0.559	0.323	0.432	0.472	0.804	0.374
**Group**	0.006	0.474	0.047	0.070	0.834	0.546	0.614	0.190	0.632	0.765
**Group and Biological replicates**	0.047	0.037	0.183	0.218	0.948	0.475	0.350	0.061	0.931	0.655

Supplementary Table 8. Wilcoxon rank sum p-values to assess the effect of potential covariates on principal components after probe filtering, normalization, and SVA correction.

	PC1	PC2	PC3	PC4	PC5	PC6	PC7	PC8	PC9	PC10
**Sentrix ID**	0.807	0.424	0.886	0.081	0.925	0.934	0.972	0.617	0.300	0.374
**Sentrix Position**	0.903	0.955	0.511	0.095	0.604	0.362	0.642	0.402	0.336	0.168
**Sample Well**	0.457	0.457	0.457	0.457	0.457	0.457	0.457	0.457	0.457	0.457
**Biological Replicate**	0.971	0.912	0.796	0.739	0.739	0.739	0.684	0.739	0.631	1.000
**Technical Replicate**	0.807	0.424	0.886	0.081	0.925	0.934	0.972	0.617	0.300	0.374
**Group**	0.002	0.004	0.712	0.668	0.775	0.998	0.009	0.060	0.958	0.998
**Group and Biological replicates**	0.048	0.083	0.904	0.655	0.109	1.000	0.065	0.266	0.953	1.000

Supplementary references

Bajar, B. T., Wang, E. S., Lam, A. J., Kim, B. B., Jacobs, C. L., Howe, E. S., . . . Chu, J. (2016). Improving brightness and photostability of green and red fluorescent proteins for live cell imaging and FRET reporting. *Sci Rep, 6*, 20889. doi:10.1038/srep20889

Chen, Y. J., Liu, P., Nielsen, A. A., Brophy, J. A., Clancy, K., Peterson, T., & Voigt, C. A. (2013). Characterization of 582 natural and synthetic terminators and quantification of their design constraints. *Nat Methods, 10*(7), 659-664. doi:10.1038/nmeth.2515

Cochran, A. G., Skelton, N. J., & Starovasnik, M. A. (2001). Tryptophan zippers: stable, monomeric beta -hairpins. *Proc Natl Acad Sci U S A, 98*(10), 5578-5583. doi:10.1073/pnas.091100898

Cong, L., Ran, F. A., Cox, D., Lin, S., Barretto, R., Habib, N., . . . Zhang, F. (2013). Multiplex genome engineering using CRISPR/Cas systems. *Science, 339*(6121), 819-823. doi:10.1126/science.1231143

Dean, D. A. (1997). Import of plasmid DNA into the nucleus is sequence specific. *Exp Cell Res, 230*(2), 293-302. doi:10.1006/excr.1996.3427

Vojta, A., Dobrinic, P., Tadic, V., Bockor, L., Korac, P., Julg, B., . . . Zoldos, V. (2016). Repurposing the CRISPR-Cas9 system for targeted DNA methylation. *Nucleic Acids Res, 44*(12), 5615-5628. doi:10.1093/nar/gkw159
