# Supplementary material for: Antagonistic and synergistic epigenetic modulation using orthologous CRISPR/dCas9-based modular system
Source: Nucleic Acids Res. 2019 Aug 14;47(18):9637–57. doi: 10.1093/nar/gkz709 (PMC6765142; doi:10.1093/nar/gkz709)
Supplement: gkz709_Supplemental_Files [file gkz709_supplemental_files.zip › SUPP_plasmid_maps.zip!/plasmid_maps/EpiToolbox_plasmids.pdf]

Plasmid	Description	Resistance	Ends	Order	Restriction	Selection
pBackBone-BZ	Backbone for assembly, ends B to Z	Ampicillin	B-Z (out)	0 (backbone for assembly)	BsaI (inverted)	blue-white
pSg-Sp	sgRNA scaffold for SpCas9, U6 promoter	Kanamycin	B-A	1	BsaI, BbsI (sgRNA)	red-white for sgRNA
pSg-Sa	sgRNA scaffold for SaCas9, U6 promoter	Kanamycin	B-A	1	BsaI, BbsI (sgRNA)	red-white for sgRNA
pPro-CBh	CBh promoter	Kanamycin	A-I	2	BsaI	none
pFD-DNMT3A	DNMT3A catalytic domain, CpG methylase	Kanamycin	I-II	3	BsaI	none
pFD-DNMT3A_NCTRL	DNMT3A catalytic domain, inactive	Kanamycin	I-II	3	BsaI	none
pFD-TET1	TET1 catalytic domain, CpG demethylase	Kanamycin	I-II	3	BsaI	none
pFD-TET1_NCTRL	TET1 catalytic domain, inactive	Kanamycin	I-II	3	BsaI	none
pFD-VPR	VPR domain, transcriptional activator	Kanamycin	I-II	3	BsaI	none
pFD-KRAB	KRAB domain, transcriptional repressor	Kanamycin	I-II	3	BsaI	none
pN-Sp_dCas9	Streptococcus pyogenes nuclease-null Cas9	Kanamycin	II-III	4	BsaI	none
pN-Sa_dCas9	Staphylococcus aureus nuclease-null Cas9	Kanamycin	II-III	4	BsaI	none
pM_AR-T2A-HygroR	Hygromycin resistance marker	Kanamycin	III-IV	5	BsaI	none
pM_AR-T2A-PuroR	Puromycin resistance marker	Kanamycin	III-IV	5	BsaI	none
pM_FP-T2A-mClover	GFP variant fluorescence marker	Kanamycin	III-IV	5	BsaI	none
pM_FP-T2A-mRuby	RFP variant fluorescence marker	Kanamycin	III-IV	5	BsaI	none
pM2-FP_T2A-X_HygroR	Hygromycin resistance marker, dual system	Kanamycin	III-X	5a	BsaI	none
pM2-FP_T2A-X_PuroR	Puromycin resistance marker, dual system	Kanamycin	III-X	5a	BsaI	none
pM2-FP_X-P2A_mClover	GFP variant fluorescence marker, dual system	Kanamycin	X-IV	5b	BsaI	none
pM2-FP_X-P2A_mRuby	RFP variant fluorescence marker, dual system	Kanamycin	X-IV	5b	BsaI	none
pTer-Bgh	Bgh transcriptional terminator	Kanamycin	IV-Z	6	BsaI	none
pSg-x1	Multiple sgRNA module, accepts 1 sgRNA	Kanamycin	B-A; B-A (out)	0 (tier 1, multi-sgRNA); 1 (in backbone)	BsaI; Esp3I (inverted)	red-white for modules
pSg-x2	Multiple sgRNA module, accepts 2 sgRNA	Kanamycin	B-A; B-I (out)	0 (tier 1, multi-sgRNA); 1 (in backbone)	BsaI; Esp3I (inverted)	red-white for modules
pSg-x3	Multiple sgRNA module, accepts 3 sgRNA	Kanamycin	B-A; B-II (out)	0 (tier 1, multi-sgRNA); 1 (in backbone)	BsaI; Esp3I (inverted)	red-white for modules
pSg-x4	Multiple sgRNA module, accepts 4 sgRNA	Kanamycin	B-A; B-III (out)	0 (tier 1, multi-sgRNA); 1 (in backbone)	BsaI; Esp3I (inverted)	red-white for modules
pSg-x5	Multiple sgRNA module, accepts 5 sgRNA	Kanamycin	B-A; B-IV (out)	0 (tier 1, multi-sgRNA); 1 (in backbone)	BsaI; Esp3I (inverted)	red-white for modules
pSg-x6	Multiple sgRNA module, accepts 6 sgRNA	Kanamycin	B-A; B-Z (out)	0 (tier 1, multi-sgRNA); 1 (in backbone)	BsaI; Esp3I (inverted)	red-white for modules
pSgM1-G	SpCas9 sgRNA scaffold, U6 promoter, position 1	Spectinomycin	B-A	1 (tier 1, multi-sgRNA assembly)	Esp3I; BbsI (sgRNA)	red-white for sgRNA
pSgM1-A	SaCas9 sgRNA scaffold, U6 promoter, position 1	Spectinomycin	B-A	1 (tier 1, multi-sgRNA assembly)	Esp3I; BbsI (sgRNA)	red-white for sgRNA
pSgM2-G	SpCas9 sgRNA scaffold, U6 promoter, position 2	Spectinomycin	A-I	2 (tier 1, multi-sgRNA assembly)	Esp3I; BbsI (sgRNA)	red-white for sgRNA
pSgM2-A	SaCas9 sgRNA scaffold, U6 promoter, position 2	Spectinomycin	A-I	2 (tier 1, multi-sgRNA assembly)	Esp3I; BbsI (sgRNA)	red-white for sgRNA
pSgM3-G	SpCas9 sgRNA scaffold, U6 promoter, position 3	Spectinomycin	I-II	3 (tier 1, multi-sgRNA assembly)	Esp3I; BbsI (sgRNA)	red-white for sgRNA
pSgM3-A	SaCas9 sgRNA scaffold, U6 promoter, position 3	Spectinomycin	I-II	3 (tier 1, multi-sgRNA assembly)	Esp3I; BbsI (sgRNA)	red-white for sgRNA
pSgM4-G	SpCas9 sgRNA scaffold, U6 promoter, position 4	Spectinomycin	II-III	4 (tier 1, multi-sgRNA assembly)	Esp3I; BbsI (sgRNA)	red-white for sgRNA
pSgM4-A	SaCas9 sgRNA scaffold, U6 promoter, position 4	Spectinomycin	II-III	4 (tier 1, multi-sgRNA assembly)	Esp3I; BbsI (sgRNA)	red-white for sgRNA
pSgM5-G	SpCas9 sgRNA scaffold, U6 promoter, position 5	Spectinomycin	III-IV	5 (tier 1, multi-sgRNA assembly)	Esp3I; BbsI (sgRNA)	red-white for sgRNA
pSgM5-A	SaCas9 sgRNA scaffold, U6 promoter, position 5	Spectinomycin	III-IV	5 (tier 1, multi-sgRNA assembly)	Esp3I; BbsI (sgRNA)	red-white for sgRNA
pSgM6-G	SpCas9 sgRNA scaffold, U6 promoter, position 6	Spectinomycin	IV-Z	6 (tier 1, multi-sgRNA assembly)	Esp3I; BbsI (sgRNA)	red-white for sgRNA
pSgM6-A	SaCas9 sgRNA scaffold, U6 promoter, position 6	Spectinomycin	IV-Z	6 (tier 1, multi-sgRNA assembly)	Esp3I; BbsI (sgRNA)	red-white for sgRNA
pUC19gg	basic plasmid for module cloning, AmpR	Ampicillin	none	none / for expansion at 1-6	none (all removed)	blue-white (from pUC19)
pUK19gg	basic plasmid for module cloning, KanR	Kanamycin	none	none / for expansion at 1-6	none (all removed)	blue-white (from pUC19)
pUS19gg	basic plasmid for module cloning, SpecR	Spectinomycin	none	none / for expansion at 1-6	none (all removed)	blue-white (from pUC19)
pBackBone-secondary-Puro	Backbone for assembly, ends B to Z	Ampicillin	B-Z (out)	0 (backbone for assembly)	BsaI (inverted)	blue-white
pPro-EFS	EFS promoter	Kanamycin	A-I	2	BsaI	none
